# Unstable Tetramic Acid Derivatives from the Deep-Sea-Derived Fungus *Cladosporium sphaerospermum* EIODSF 008

**DOI:** 10.3390/md16110448

**Published:** 2018-11-15

**Authors:** Xiao Liang, Zhong-Hui Huang, Xuan Ma, Shu-Hua Qi

**Affiliations:** 1 CAS Key Laboratory of Tropical Marine Bio-Resources and Ecology, Guangdong Key Laboratory of Marine Materia Medica, RNAM Center for Marine Microbiology, South China Sea Institute of Oceanology, Chinese Academy of Sciences, 164 West Xingang Road, Guangzhou 510301, Guangdong, China; liangxiao213@mails.ucas.ac.cn (X.L.); zhonghui23@hotmail.com (Z.-H.H.); maxuan@scsio.ac.cn (X.M.); 2 University of Chinese Academy of Sciences, Beijing 100049, China

**Keywords:** deep-sea-derived fungus, *Cladosporium sphaerospermum*, tetramic acid, cladosporiumins I-O

## Abstract

Seven new unstable tetramic acid derivatives, cladosporiumins I-O (**1**–**7**), together with the known analogue cladodionen (**8**) were isolated from the extract of the deep-sea-derived fungus *Cladosporium sphaerospermum* EIODSF 008. Their structures were elucidated by spectroscopic analysis, quantum chemical calculations and ECD spectra. Compound **4** was a Mg complex of tetramic acid derivative. In acidic solvent, **4** could change to **1** and **6**, and **7** could change to **5**. In addition, **1**, **5** and **8** existed as two exchangeable isomers, respectively. The structures of cladosporiumins E-H were reassigned as their Na complexes. The antibacterial and cytotoxic activities of **1**–**8** were also evaluated. However, because of their instability, all of the isolated compounds did not show significant antibacterial activity as the preliminary EtOAc extracts of the fungal strain.

## 1. Introduction

**Figure 1:**
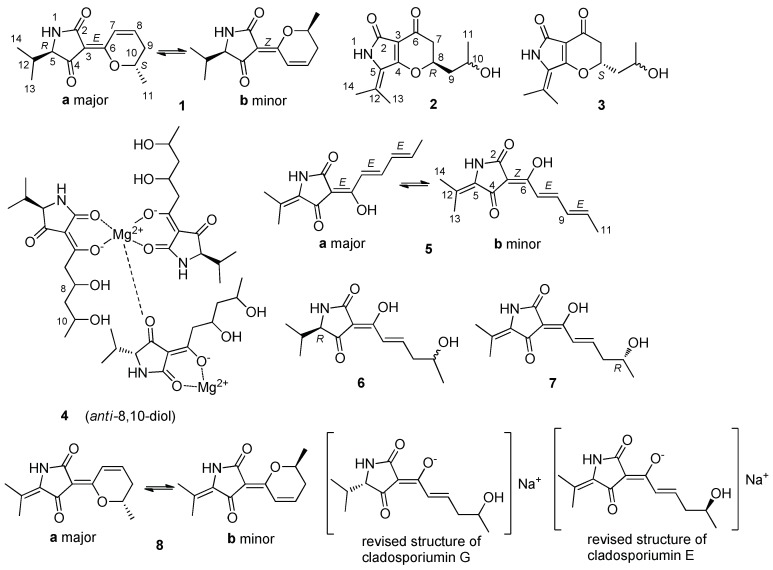
Structures of compounds **1**–**8**.

Tetramic acid refers to the heterocyclic system pyrrolidine-2,4-dione that is a tautomer of 1,5-dihydro-4-hydroxy-2*H*-pyrrol-2-one and the predominant type in solution [1]. Many natural products containing the structure unit have been isolated from marine mollusks, sponges and cyanobacteria, terrestrial and marine microorganisms, which have been found to display diverse bioactivities [2,3]. These kinds of compounds show weak acidic properties and have four tautomer forms in solvent [4,5,6]. Some of them could form stable complexes with transition metal ions [1], such as magnesium and calcium complexes [7,8,9]. The genus *Cladosporium* could produce a series of exchangeable tetramic acid derivatives, such as cladosins [10,11] and cladodionen [12], showing antiviral and cytotoxic activities. In order to explore novel bioactive compounds from marine fungi, we studied the secondary metabolites of the deep-sea-derived fungus *Cladosporium sphaerospermum* EIODSF 008 that showed significant antibacterial activity in preliminary experiments. Finally, seven new unstable tetramic acid derivatives cladosporiumins I-O (1–7) (Figure 1) together with the known analogue cladodionen (8) [12] were obtained from the strain. The antibacterial and cytotoxic activities of these compounds were evaluated. In addition, the structures of cladosporiumins E-H [13] previously reported were reassigned as their Na complexes. Details of the isolation, structure elucidation and bioactivity screening are reported here.

## 2. Result and Discussion

Cladosporiumin I (**1**) was isolated as a pair of exchangeable compounds **1a** and **1b** present in a ratio of about 1:0.7. The HR-ESI-MS exhibited the quasi-molecular ion at *m*/*z* 258.1098 [M + Na]^+^, which suggested that **1a** and **1b** had the same molecular formula of C_13_H_17_NO_3_, requiring six degrees of unsaturation. The ^1^H and ^13^C nuclear magnetic resonance (NMR) spectra (Table 1 and Table 2) showed two sets of signals. Heteronuclear singular quantum correlation (HSQC) signals revealed the presence of three doublet methyl groups, one methylene, five methines including one oxygenated methine and one double bond, one amide carbonyl and one *α*, *β*-unsaturated ketone in each compound. These data showed great similarity to that of **8** that contained two sets of signals for its *E*-Δ^3(6)^ major isomer and *Z*-Δ^3(6)^ minor isomer [12], respectively and the only obvious difference between **1** and **8** was the double bond between C-5 and C-12 reduced into be a single bond in **1**. This was supported by the 2D NMR data. Here, the data of the major compound **1a** was analyzed in detail.

**Figure 2:**
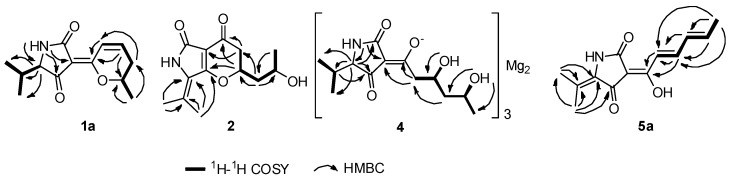
Key ^1^H-^1^H COSY and HMBC correlations of compounds **1a**, **2**, **4**, **5a**.

In the ^1^H-^1^H COSY spectrum (Figure 2), correlations of H-8 with H-7/H-9, H-10 with H-9/H-11 and H-12 with H-13/H-14/H-5 established the fragments of C7-C8-C9-C10-C11 and C(13/14)-C12-C5, which was supported by the key heteronuclear multiple bond correlation (HMBC) signals (Figure 2) from H-5 to C-12/C-13/C-14, H-7/H-8 to C-6, H-8/H-11 to C-10, H-9 to C-7 and H-11 to C-9. In addition, HMBC correlations from NH-1 to C-3/C-4/C-5 confirmed the presence of the pyrrolidine-2, 4-dione ring system. 

**Figure 3:**
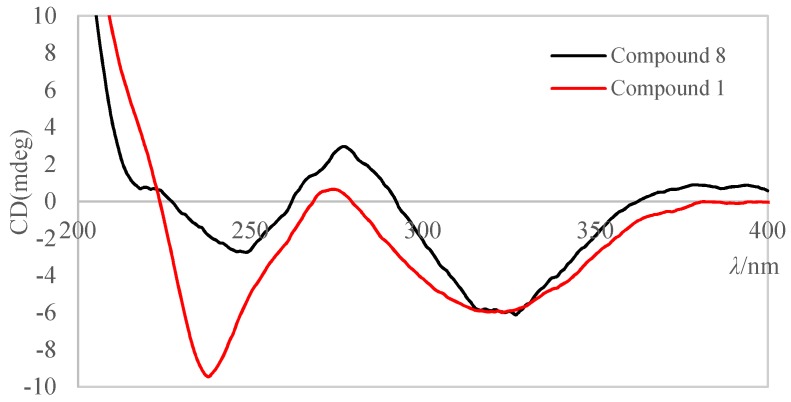
Comparison between the measured ECD spectra of **1** and **8** in CH_3_OH.

The main difference between the two isomers of **1a** and **1b** was the chemical shifts of C-2 (*δ*_C_ 169.6/167.9) and C-4 (*δ*_C_ 196.4/199.6), which indicated that **1a** and **1b** were a pair of rapidly interconverting *Z*/*E* geometric isomers in solution. For compound **8**, *E*-Δ^3(6)^ and *Z*-Δ^3(6)^ configurations were assigned to its major isomer and minor isomer, respectively, by theoretical calculations of energy [12]. A similar method was used to determine the configuration of the double bond between C-3 and C-6 in structures **1a** and **1b**. The results (Tables S1 and S2) indicated that the stable conformers of **1a** had lower free energies than those of **1b** and **1a** possessed higher equilibrium populations than that of **1b**, which confirmed the major isomer **1a** and the minor isomer **1b** had *E*-Δ^3(6)^ and *Z*-Δ^3(6)^ configuration, respectively. In addition, the double bond between C-7 and C-8 was determined as *Z* configuration according to the coupling constant *J*_H-7,H-8_ = 10.0 Hz. So, the planar structure of **1** was assigned as shown. According to literatures, the natural products bripiodionen [14] and vermelhotin [15] also have the homologous skeleton. The absolute configuration of C-5 in **1** was further determined as *R* due to **1** derived from **4** (see details in discussion about **4**). And the absolute configurations of C-10 in **1a** and **1b** were determined as *S*, because the measured ECD spectrum of the mixture **1a** and **1b** (Figure 3) showed greatly similar cotton effects as that of **8** [12]. In this case, the structure of **1** was determined as shown.

Cladosporiumin J (**2**) had the molecular formula of C_13_H_17_NO_4_ based on the HR-ESI-MS (*m*/*z* 274.1053 [M + Na]^+^). The ^1^H and ^13^C NMR data of **2** (Table 1 and Table 2) showed similarity to that of **8**, which indicated that **2** contained a 2, 4-pyrrolidione unit. In the ^1^H-^1^H COSY spectrum, correlations of H-8 with H-7/H-9 and H-10 with H-9/H-11 suggested the presence of the C7-C8-C9-C10-C11 fragment. In addition, the HMBC spectrum showing correlations from H-8 to C-4 and C-6 and from H-7 to C-3 and C-6 suggested that C-4 and C-8 were linked with an oxygen atom to form a *γ*-pyrone fragment. So, the planar structure of **2** was determined as shown, containing the same core as cordylactam [16], pyranonigrins [17,18], phaeosphaeride [19,20] and paraphaeosphaeride [21].

**Figure 4:**
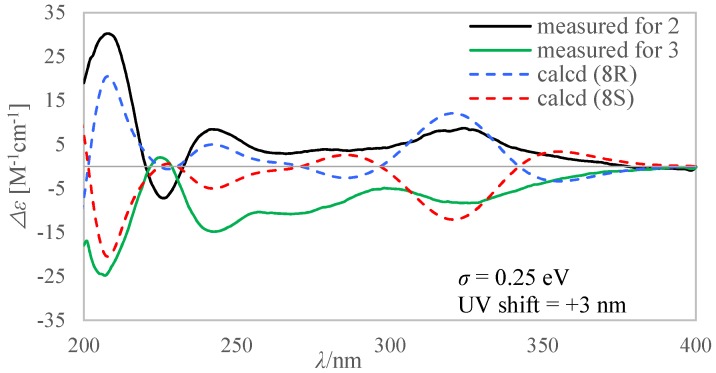
Comparison between calculated and measured ECD spectra of **2** and **3** in CH_3_OH.

Cladosporiumin K (**3**) had the same molecular formula of C_13_H_17_NO_4_ as **2** based on the HR-ESI-MS (*m*/*z* 274.1055 [M + Na]^+^). The ^1^H and ^13^C NMR data of **3** (Table 1 and Table 2) were almost the same as that of **2** and between them only the chemical shifts of C-8/C-10/C-11 had obvious differences of about 0.6~0.9 ppm. 2D NMR data proved that **3** had the same planar structure as **2**. The specific optical rotation values of **2** and **3** were [*α*]${}_{D}^{25}$ +79.0 (*c* 0.100, CH_3_OH) and [*α*]${}_{D}^{25}$ −66.6 (*c* 0.100, CH_3_OH), respectively and their ECD spectra (Figure 4) also showed perfectly opposite cotton effects, which indicated that **2** and **3** were a pair of epimers. The absolute configuration of C-8 was determined by the calculated ECD spectra. As shown in Figure 4, C-8 in 2 and 3 was assigned as *R* and *S* configuration, respectively. We failed to determine the absolute configuration of C-10 by Mosher method because of the instability of their Mosher ester products.

**Figure 5:**
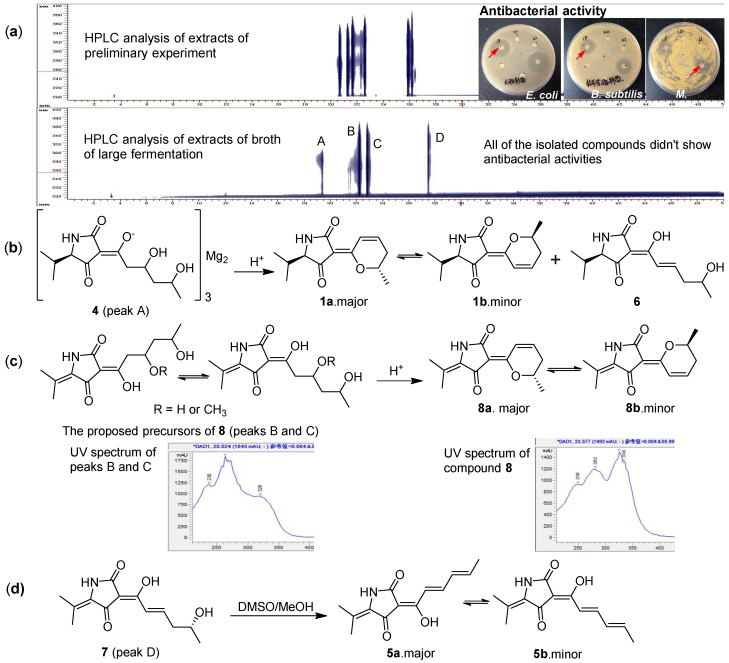
The chemical transformation relationship of compounds **1** and **4**–**8**. (**a**) HPLC analysis of extracts of preliminary experiment and large fermentation; (**b**) Chemical transformation relationship between compound **4** and compounds **1** and **6**; (**c**) Proposed precursors of compound **8**; (**d**) Chemical transformation relationship between compound **7** and compound **5**.

Cladosporiumin L (**4**) had the molecular formula of (C_13_H_20_NO_5_)_3_Mg_2_ as determined by its NMR data, positive HR-ESI-MS giving a strong fragment peak at *m*/*z* 858.3701 (calcd. for C_39_H_60_N_3_O_15_Mg_2_, 858.3720) and ICP-AES analysis (see Supporting Information Figures S51 and S26). Compound **4** was relatively stable in a neutral solvent system, while unstable in acidic solvent. In preliminary experiment, **4** was purified by pre-HPLC on an ODS column eluting with CH_3_CN-H_2_O (containing 0.03% TFA) but when dried, it was found that **4** changed into other three compounds including **1**, **6** and an unknown compound (Figure 5 and Figure S50). The ^1^H and ^13^C NMR spectra of **4** showed similarity to that of **1** and **6** and two sets of ^1^H and ^13^C NMR signals were also observed in **4** with a ratio of about 1:0.5 (**4a**:**4b**). The most obvious differences between **1** and **4** were the presence of two hydroxyl protons (*δ*_H_ 4.30/4.30, 8-OH; *δ*_H_ 4.30/4.34, 10-OH) in **4** and the C-7/C-8 double bond in **1** changed to a single bond in **4** (*δ*_C_ 46.1, C-7; *δ*_C_ 66.2/66.0, C-8). The sequential COSY correlations of H-7/H-8/H-9/H-10/H-11, H-8 with 8-OH and H-10 with 10-OH established the 1,3-dihydroxy-pentanol fragment in the side chain. The HMBC correlations from H-7 to C-3/C-6 suggested the 1,3-dihydroxy-pentanol fragment was connected to the pyrrolidine-2,4-dione unit by the C-3/C-6 double bond. So, the planar structure of **4a** and **4b** in **4** was established as shown with the same molecular formula of C_13_H_21_NO_5_. Previous studies reported that 3-acetyl tetramic acid and 3-acetyl tetronic acid derivatives could form stable complexes with ions of metals [1,2,22,23,24,25]. In here, according to HR-ESI-MS giving a strong fragment peak at *m*/*z* 858.3701 (calcd. for C_39_H_60_N_3_O_15_Mg_2_, 858.3720), we speculated that **4** was a Mg complex. This was supported by ICP-AES analysis showing the content of Mg 4.02 wt% in **4** (Figure S51).

Comparing with cladosporiumin H (C-2, *δ*_C_ 177.1) and other analogues [13,22,26,27], the low-field chemical shifts of C-2 (*δ*_C_ 177.6/177.7) in **4** suggested a *Z*-Δ^3(6)^ configuration in **4**. The ^1^H and ^13^C NMR data of **4a** and **4b** were very similar and the obvious difference between them was the small migration of the chemical shifts of H-5 (*δ*_H_ 3.37/3.42), H-7 (*δ*_H_ 2.81/2.85), H-8 (*δ*_H_ 4.03/4.06), H-9 (*δ*_H_ 1.36/1.41), 10-OH (*δ*_H_ 4.30/4.34) and 1-NH (*δ*_H_ 7.73/7.80). The overlapped chemical shifts for the geminal proton of H-9 at *δ*_H_ 1.36 and 1.41 in **4a** and **4b**, respectively, indicated that both of them had an *anti* 1,3-diol unit between C-8 and C-10 [11,28], which was supported by the chemical shift of C-8 (*δ*_C_ 66.2/66.0 in DMSO-*d*_6_) matching the typical value in Kishi’s Universal NMR Database for an *anti* 1,3-diol unit (*δ*_C_ 66.6 in DMSO-*d*_6_) [29]. But the absolute configurations of C-8/C-10 in the 1,3-diol unit were not determined. The valine unit was identified as D-Val by Marfey’s method (see Supporting Information Figures S48 and S49), which suggested that C-5 was *R* configuration. Previous studies reported that formation of metal complexes of tetramic acid derivatives (such as tenuazonic acid and melophlins A and C) could obviously affect the chemical shifts of H-5 and *N*-methyl protons or NH [23,25] and the Mg complex of tenuazonic acid (TA-H) was speculated to be a mixture of Mg(TA)_2_ and Mg(TA)^+^ [25]. And the structure of the copper complex of tenuazonic acid was revealed to be Cu_2_(TA)_3_ by X-ray diffraction [30]. Based on the above data and comparison with literatures, it was more reasonable to speculate the structure of **4** with the molecular formula of (C_13_H_2__0_NO_5_)_3_Mg_2_ as shown rather than a mixture of (C_13_H_2__0_NO_5_)_2_Mg and (C_13_H_2__0_NO_5_)Mg with the ratio of 1:1 based on the ratio of the two sets of signals observed in the ^1^H NMR spectrum. Of course, it will be better if we can prove the speculation by X-ray diffraction.

Some tetramic acid magnesium complexes were also isolated from marine organisms, such as magnesidin [7,31], geodin A [9] and ancorinoside A Mg salt [8]. It was reported that the salts could be converted to its conjugate acid by using acidic solvent for isolation [7,8]. But some of the conjugate acids derived from the salts (e.g., geodin A) were unstable and prone to oxidation [9]. Compound **4** also exhibited the similar properties.

Cladosporiumin M (**5**) had the molecular formula of C_13_H_15_NO_3_ based on the HR-ESI-MS (*m*/*z* 234.1135 [M + H]^+^). The ^1^H and ^13^C NMR data of **5** (Table 1 and Table 2) also showed two sets of signals with a ratio of about 1.0:0.6 for **5a**:**5b**. The 1D NMR data of **5** were similar to that of cladosporiumin E [13] and the only obvious difference between them was the additional presence of one double bond (**5a**: *δ*_C_ 131.2, 142.7; **5b**: *δ*_C_ 131.2, 142.4) and the disappearance of one methene and one oxygenated methine. The geometric isomers of **5a** (major) and **5b** (minor) were assigned as *E*-Δ^3(6)^ and *Z*-Δ^3(6)^ form by comparing the chemical shifts of C-2 (**5a**: *δ*_C_ 166.0, **5b**: *δ*_C_ 172.1) and C-4 (**5a**: *δ*_C_ 188.6, **5b**: *δ*_C_ 182.4), respectively. A larger coupling constant *J*_H-7,H-8_ = 15.5 Hz and *J*_H-9,H-10_ = 14.5 Hz suggested *E* configuration of Δ^7(8)^ and Δ^9(10)^.

Cladosporiumin N (**6**) had the molecular formulas of C_13_H_19_NO_4_ as determined by HR-ESI-MS (*m*/*z* 276.1205 [M + Na]^+^). Analysis of the 1D and 2D NMR data (Table 3) suggested that **6** had the same planar structure as cladosporiumin G [13]. The carbon signals of C-2/C-3/C-4/C-5 were very weak in the ^13^C NMR spectrum of **6**, which was different from cladosporiumin G. And the ^1^H and ^13^C NMR data of C-2/C-3/C-4/C-5 and specially C-6/C-7/C-8 were obviously different between **6** and cladosporiumin G. Because **4** could change into **6**, the Val residue in **6** was also deduced to be *D*-form, which suggested that C-5 in **6** was *R* configuration. The absolute configuration of C-5 in cladosporiumin G was determined as *S* by ECD calculation [13]. So, it seemed that **6** was a configurational isomer of cladosporiumin G at C-5 and/or C-10. We have also tried to determine the absolute configurations of C-10 by modified Mosher’s method, however the experiments failed as that of cladosporiumins A–C and cladosporiumin G [13].

Cladosporiumin O (**7**) had the molecular formulas of C_13_H_17_NO_4_ as determined by HR-ESI-MS (*m*/*z* 274.1051 [M + Na]^+^). Analysis of the 1D and 2D NMR data (Table 3) suggested that **7** had the same planar structure as cladosporiumin E [13]. The carbon signals of C-3/C-5/C-7/C-8/C-12 were very weak or broad in the ^13^C NMR spectrum of **7**, moreover, the signals of C-2/C-4/C-6 were neither observed in the ^13^C NMR nor HMBC spectrum of **7**, which was different from cladosporiumin E. In addition, the ^1^H and ^13^C NMR data of C-3/C-5 and specially C-7/C-8 were also obviously different between **7** and cladosporiumin E. However, the ^13^C NMR data of C-6/C-7/C-8 in **6** and **7** were close to that of cladosin C [11]. Comparison of the specific optical rotations of **7** ([*α*]${}_{D}^{25}$ −10.0 (*c* 0.123, CH_3_OH)), cladosin C ([*α*]${}_{D}^{25}$ +10.5 (*c* 0.10, CH_3_OH)) and cladosporiumin E ([*α*]${}_{D}^{25}$ + 2.7 (*c* 0.10, CH_3_OH)) suggested that the configuration of C-10 in **7** was *R* instead of *S* as that in cladosin C and cladosporiumin E, which indicated that **7** was an enantiomer of cladosporiumin E at C-10. Although the carbon signals of C-2/C-4/C-6 did not appear in the ^13^C NMR spectrum of **7**, it was observed that **7** could dehydrate and precipitate solid in solvent (DMSO or CH_3_OH), slowly and spontaneously, to form **5** (Figure 5), implying **7** had the same tetramic acid skeleton as **5**.

The HR-ESI-MS data of cladosporiumin E [13] suggested that the structure of cladosporiumin E should be reassigned as its sodium salt, because the HRESIMS of cladosporiumin E (see Figure S52) showed fragment peaks at *m*/*z* 274.1051 [M + H]^+^ and 296.0873 [M + Na]^+^ which were mistaken for [M + Na]^+^ and [M − H + 2Na]^+^ in the literature [13], respectively. Similarly, the structure of cladosporiumin G also should be reassigned as its sodium salt, because the HRESIMS of cladosporiumin G (see Figure S53) showed fragment peaks at *m*/*z* 276.1205 [M + H]^+^ and 298.1031 [M + Na]^+^ which were mistaken for [M + Na]^+^ and [M − H + 2Na]^+^ in the literature [13], respectively. After the correction, it was more reasonable to explain the obvious differences of ^1^H and ^13^C NMR data of C-2/C-3/C-4/C-5 and specially C-6/C-7/C-8 between **7** and cladosporiumin E and between **6** and cladosporiumin G. Except that, cladosporiumins F and H^13^ also should be reassigned as their complexes of sodium based on their HRESIMS (see Figures S54 and S55) and NMR data [13]. Several synthetic tetramic acid metal complexes have been previously reported [22,23,25], such as the complexes of the natural melophlins A and C with Mg (II), Zn (II), Ga (III), La (III) and Ru (II) [23] and the complexes of tenuazonic acid with Cu (II), Fe (III), Ni (II) and Mg (II) [25]. Comparing with the NMR data of the free tenuazonic acid and melophlins A and C, in their metal complexes the chemical shifts of H-5 and *N*-methyl protons shifted to upfield obviously [23,25] and the chemical shifts of C-2/C-6 (numbered as C-1′ in the literature) shifted to downfield significantly [23]. The chemical shifts of H-5 and C-2/C-6 in compound **6** and cladosporiumin G displayed the similar rules. Cladosin C^11^ was an analogue of **6** and **7**, which was not a metal complex. The chemical shifts of C-6/C-7/C-8 in **6** and **7** were similar to that of cladosin C but quite different from that of cladosporiumins G and E, which indicated that the formation of sodium complexes led to the downfield migration of the chemical shifts of C-6 and C-7 and the upfield migration of the chemical shift of C-8. However, no reasonable explanation for the phenomena that the formation of metal complex can obviously affect the chemical shifts of H-5, C-2, C-4, C-6, C-5, C-7 and C-8 was known.

In the primary HPLC analysis spectrum of the broth (Figure 5), the two peaks B and C (*t*_R_ = 22.0 min and 23.0 min, respectively) were changed into other two compounds (namely, **8a** and **8b**) when the fraction was separated by ODS column eluting with acidic solvent. So the two principal compounds did not been obtained, however, according to the similar phenomenon observed between **4** and **1**, we speculated the structures of the two principal compounds as shown in Figure 5.

Compounds **1**–**5** and **8** were evaluated for their cytotoxicity against human cancer cell lines HL-60, HepG2 and MCF-7 and compounds **1**–**8** were evaluated for their antibacterial activity against *Escherichia coli*, *Bacillus subtilis* and *Micrococcus luteus*. Only **8** showed cytotoxicity towards HL-60 cell line with IC_50_ value of 28.6 μM, which was close to the reported data [12]. The other compounds did not show any obvious cytotoxicity. In the primary experiment, the EtOAc extracts of *Cladosporium* sp. EIODSF 008 exhibited significant antibacterial activities against *E. coli*, *B. subtilis* and *M. luteus* at 100 μg/disc with disc diffusion method (Figure 5 and Figure S56), however, all of the isolated compounds did not show antibacterial activity against the above tested bacteria at 50 μg/disc. The reason may be the instability of these compounds, suggesting the antibacterial compounds probably have changed during the courses of extraction and purification.

## 3. Experimental Section

### 3.1. General Experimental Procedures

Optical rotations were measured with a MCP 500 polarimeter (Anton Paar). UV and IR spectra were recorded using a UV-2600 UV-vis spectrophotometer (Shimadzu) and an IR Affinity-1 Fourier transform infrared spectrophotometer (Shimadzu). ECD spectra were measured with a Chirascan circular dichroism spectrometer (Applied Photophysics Ltd., Surrey, UK). ^1^H, ^13^C NMR and 2D NMR spectra were acquired with a Bruker AV-500 MHz NMR spectrometer (Bruker, Billerica, MA, USA) with TMS as reference. ESIMS and HRESIMS spectroscopic data were acquired with an amaZon SL ion trap mass spectrometer and MaXis quadrupole-time-of-flight mass spectrometer (Bruker), respectively. Semi-preparative reversed-phase (SP-RP) HPLC was performed on a Shimadzu LC-20A preparative liquid chromatography system with an YMC-Pack ODS column, 250 × 20 mm, S-5 μm, 12 nm. RP-MPLC (reversed-phase-medium pressure preparative liquid chromatography) was carried out using the CHEETAH MP200 system (Agela Technologies, Wilmington, DE, USA) and Claricep Flash columns filled with ODS (40–63 μm, YMC). Sephadex LH-20 (GE Healthcare, Chicago, IL, USA) was used for column chromatographic column (CC). Silica gel (200–300 mesh) for CC and GF254 for TLC were obtained from Yantai Jiangyou Silica Gel Development Co. Ltd. (Yantai, China). Optima 8300 ICP-AES (PerkinElmer, Waltham, MA, USA) was used for assaying magnesium content.

### 3.2. Fungal Materials

The fungus *C. sphaerospermum* EIODSF 008 was isolated from a deep sea sediment collected from East Indian Ocean (10°00′ N and 84°33′ E, 4571 m depth) possessing a GenBank accession number of KJ173531. The strain was identified by ITS rDNA sequence and deposited in the RNAM Center, South China Sea Institute of Oceanology, Chinese Academy of Science [32].

### 3.3. Fermentation and Extraction

The fungus *C. sphaerospermum* EIODSF 008 was cultured on potato dextrose agar (PDA) plates at 28 °C for 5 days. Then the spores were collected and suspended in sterile water, after which the suspension was transferred into 120 × 0.5 L Erlenmeyer flasks each containing 200 mL liquid medium (2% glucose, 20% potato, 3% sea salt). The flasks were incubated at 28 °C at 200 rmp on a rotary shaker. After 6 days, the fungal mycelia and broth were separated with cheesecloths. The mycelia were extracted with acetone. The acetone extract was concentrated under reduced pressure to give aqueous phase which was extracted with ethyl acetate to yield a crude extract (40 g). The fermentation broth was mixed with XAD-16 reins (20 g/L) and stirred for an hour. After that, resins were separated with the residual liquid and washed with running water to remove culture medium component. Then the resins were washed with ethanol (6 L) which was further concentrated to give a crude extract (8 g).

### 3.4. Isolation and Purification

The combined extract (48 g) was subjected to a silica gel column using a stepped gradient elution of CHCl_3_/CH_3_OH to give six sub-fractions (Fr.1-Fr.6). Fr.4 (8 g) was separated by ODS column eluting with CH_3_OH/H_2_O/TFA (from 20:80:0.03 to 100:0:0.03) to yield fifteen sub-fractions (Fr.4-6-1-Fr.4-6-15). Fr.4-6-4 and Fr.4-6-5 were purified by preparative TLC, applying CHCl_3_/CH_3_OH (5:1) as developing solvent to afford **3** (19.7 mg, *R_f_* = 0.5) and **2** (32.0 mg, *R_f_* = 0.5), respectively. Fr.4-6-11 was isolated by preparative TLC (CHCl_3_:CH_3_OH = 25:4) and further purified by Sephadex LH-20 eluding with CH_3_OH to give **7** (37 mg, *R_f_* = 0.3). The DMSO or CH_3_OH solution of **7** spontaneously precipitated to a yellow powder, which was filtered to give **5** (10 mg). Fr.4-7 was purified by Sephadex LH-20 eluding with CH_3_OH to yield **4** (34.0 mg). Compound **4** was unstable in an acidic solvent and changed into a mixture of three compounds and then the mixture was further purified by preparative TLC (CHCl_3_:CH_3_COCH_3_:HCOOH = 4:1:0.1) to obtain **6** (26.7 mg, *R_f_* = 0.6) and **1** (101.0 mg, *R_f_* = 0.8). Fr.4-9 was filter and washed by methanol to give a yellow powder (about 1 g) that contained two peaks (peaks B and C in Figure 5). We attempted to separate them by preparative HPLC eluding with CH_3_CN/H_2_O/TFA (22:78:0.03) to get the major component **8a** (*t*_R_ = 37.0 min) and the minor one **8b** (*t*_R_ = 43.0 min). But after the isolation, the two compounds could interchange. Actually, the phenomenon also happened in compound **1** (**1a** and **1b**).

**Cladosporiumin I (1):** Yellow oil; [*α*]${}_{D}^{25}$ −95 (*c* 0.135, CH_3_OH); UV (CH_3_OH) *λ*_max_ (log *ε*) 203 (3.78), 292 (3.69), 325 (3.75) nm; ECD (CH_3_OH) *λ*_max_ (∆*ε*) 238 (−33.65), 274 (+2.35), 323 (−21.34) nm; ^1^H and ^13^C NMR data see Table 1 and Table 2; HRESIMS *m*/*z* 258.1098 [M + Na]^+^ (calcd. for C_13_H_17_NO_3_Na, 258.1101).

**Cladosporiumin J (2):** White powder; [*α*]${}_{D}^{25}$ +79 (*c* 0.100, CH_3_OH); UV (CH_3_OH) *λ*_max_ (log *ε*) 203 (3.76), 310 (3.58) nm; ECD (CH_3_OH) *λ*_max_ (∆*ε*) 208 (+30.22), 226 (−7.21), 242 (+8.43), 324 (+8.71) nm; IR (film) ν_max_ 3354, 1705, 1634, 1614, 1557, 1456, 1373, 1360, 1099, 1024 cm^−1^; ^1^H and ^13^C NMR data see Table 1 and Table 2; HRESIMS *m*/*z* 274.1053 [M + Na]^+^ (calcd. for C_13_H_17_NO_4_Na, 274.1050). 

**Cladosporiumin K (3):** White powder; [*α*]${}_{D}^{25}$ −67 (*c* 0.100, CH_3_OH); UV (CH_3_OH) *λ*_max_ (log *ε*) 202 (3.74), 311 (3.35) nm; ECD (CH_3_OH) *λ*_max_ (*∆ε*) 207 (−24.81), 225 (+2.03), 242 (−14.81), 326 (−8.32) nm; IR (film) ν_max_ 3265, 1709, 1651, 1609, 1556, 1456, 1373, 1362, 1099, 1024 cm^−1^; ^1^H and ^13^C NMR data see Table 1 and Table 2; HRESIMS *m*/*z* 274.1055 [M + Na]^+^ (calcd. for C_13_H_17_NO_4_Na, 274.1050).

**Cladosporiumin L (4):** Pale yellow solid; [*α*]${}_{D}^{25}$ −58 (*c* 0.198, CH_3_OH); UV (CH_3_OH) *λ*_max_ (log *ε*) 202 (4.06), 239 (4.01), 282 (4.23) nm; IR (film) ν_max_ 3308, 1668, 1607, 1485, 1418, 1202, 1184, 1138, 1024 cm^−1^; ^1^H and ^13^C NMR data see Table 1 and Table 2; HRESIMS *m*/*z* 858.3701 [M]^+^ (calcd. for C_39_H_60_N_3_O_15_Mg_2_, 858.3720).

**Cladosporiumin M (5):** Yellow powder; UV (CH_3_OH) *λ*_max_ (log *ε*) 256 (4.71), 286 (4.84), 353 (4.98) nm; IR (film) ν_max_ 3175, 1674, 1609, 1580, 1367, 1256, 1219, 1169, 1132, 1090, 1063, 1007, 959, 889, 762 cm^−1^; ^1^H and ^13^C NMR data see Table 1 and Table 2; HRESIMS *m*/*z* 234.1135 [M + H]^+^ (calcd. for C_13_H_16_NO_3_, 234.1125).

**Cladosporiumin N (6):** Pale yellow oil; [*α*]${}_{D}^{25}$ −75 (*c* 0.113, CH_3_OH); UV (CH_3_OH) *λ*_max_ (log *ε*) 202 (3.77), 231 (3.64), 324 (3.85) nm; ^1^H and ^13^C NMR data see Table 3; HRESIMS *m*/*z* 276.1205 [M + Na]^+^ (calcd. for C_13_H_19_NO_4_Na, 276.1206).

**Cladosporiumin O (7):** Yellow oil; [*α*]${}_{D}^{25}$ −10 (*c* 0.123, CH_3_OH); UV (CH_3_OH) *λ*_max_ (log *ε*) 240 (3.81), 280 (3.98), 333 (3.84) nm; ^1^H and ^13^C NMR data see Table 3; HRESIMS *m*/*z* 274.1051 [M + Na]^+^ (calcd. for C_13_H_17_NO_4_Na, 274.1050).

### 3.5. Analysis of Magnesium Content in ***4***

Compound **4** (1.67 mg) was acid-digested with 300 μL concentrated nitric acid at 120 °C for 3 h, until the acid solvent stopped emitting brown gas and turned into transparent solution. The liquid was made up to 10 mL with deionized water, then the magnesium concentration was analyzed by ICP-AES (inductively coupled plasma atomic emission spectroscopy). Blank control (without sample) was treated in the same way. The concentration of Mg in **4** and blank control was determined as 6.79 and 0.073 mg/L, respectively, which equaled to **4** contain 4.02 wt% Mg. 

### 3.6. Determination of Absolute Configuration of the Valine Unit in ***4***

Compound **4** (0.48 mg) was dissolved in 1 mL of 6 N HCl and heated at 115 °C for 21 h. The hydrolysate was extracted with ethyl acetate to remove organic substance and obtain aqueous phase. Then it was dried and dissolved in 100 μL water. 20 μL of 1 M NaHCO_3_ and 100 μL of 2 mg/mL FDAA acetone solution was added and the mixture was incubated at 40 °C for 1 h. The reaction was quenched by the addition of 20 μL of 2 N HCl, then the dried mixture was dissolved in methanol for HPLC analyzing. The standard D/L-Val were also derivative with FDAA with the same way. All of the samples were analyzed with HPLC under the following conditions: YMC-Pack ODS-A column, 250 × 4.6 mm, S-5 μm, 12 nm; mobile phase: CH_3_CN/H_2_O (0.03% TFA in H_2_O), linear gradients started with 15% CH_3_CN and finished with 45% CH_3_CN in 45 min; flow rate was 1 mL/min, with UV detection at an absorbance of 340 nm [33]. Retention times for the FDAA derivate of standards were 28.0 min for L-Val and 34.8 min for D-Val. An obvious peak at 34.9 min was observed in the FDAA derivate of hydrolysate of **4** suggesting the Val was the D-form.

### 3.7. Computational Methods to Calculate the ECD Spectra of 2 and 3 and the Equilibrium Populations of ***1a*** and ***1b***

Spartan 14 program (Wavefunction Inc., Irvine, CA, USA) was used for calculating molecular Merck force field (MMFF). Gaussian 16A program package was used for density functional theory (DFT) and time-dependent density functional theory (TDDFT) calculations. The conformational search was performed by a MMFF model, then the conformers with lower energy (<5 kcal/mol) were subjected to geometry optimization with the DFT method at the B3LYP/6-31G(d) level. Vibrational frequency calculations were done at the same level to evaluate their relative free energies (Δ*G*) at 298.15 K. To obtain the energies of these low energy conformers in methanol, the geometry optimized conformers were further calculated at the M06-2X/def2-TZVP level and the solvent (methanol) effects were taken into consideration by using solvation model based on density (SMD). The optimized conformers with the Boltzmann distribution more than 1% population were subjected to ECD calculation, which were performed by TDDFT methodology at the PBE1PBE/6-311g(d) level. Thirty-two excited states were considered in these calculations. The ECD spectra were generated by the software SpecDis using a Gaussian band shape with 0.25–0.35 eV exponential half-width from dipole-length dipolar and rotational strengths. Equilibrium population of each conformer at 298.15 K was calculated from its relative free energies using Boltzmann statistics. Theoretical spectra of compound **2** and **3** were obtained after correction of the Boltzmann distribution of each low-energy conformer in MeOH solution according to the free energy.

## 4. Conclusions

In conclusion, seven new unstable tetramic acid derivatives cladosporiumins I-O (**1**–**7**) together with a known analogue cladodionen (**8**) were isolated from a liquid cultural product of the deep-sea-derived fungus *C. sphaerospermum* EIODSF 008. Besides, four previously reported compounds cladosporiumins E-H were reassigned as their sodium complexes. This kind of metabolites produced by the fungus are unstable in the acidic solvent. Their structure transformations mainly included dehydration, forming or decomposing metal complexes and spontaneous *Z*/*E* interchange of the double bonds. Unfortunately, we did not obtain the compounds exhibiting the same significant antibacterial activities as the EtOAc extracts of the fungal strain obtained in the preliminary experiment. It was speculated the bioactive compounds have changed during the courses of extraction and purification on account of their instability.

## Figures and Tables

**Table 1:** ^1^H (500 MHz) nuclear magnetic resonance (NMR) Data for **1**–**5** (*δ* ppm).

	*δ*_H_ (*J* in Hz)							
Compound	1 ^a^		2 ^a^	3 ^a^	4 ^a^		5 ^b^	
Position	a	b			a	b	a	b
1-NH	8.15 (s)	7.97 (s)	9.54 (s)	9.54 (s)	7.73 (s)	7.80 (s)	8.12 (s)	8.64 (s)
5	3.52 (d, 3.0)	3.58 (d, 3.0)			3.37 (s)	3.42 (s)		
7	7.75 (dd, 10.0, 2.0)	7.56 (dd, 10.0, 2.0)	2.44 (dd, 16.5, 3.5) 2.59 (dd, 16.5, 12.5)	2.47 (dd, 17.0, 3.5) 2.61 (dd, 16.5, 12.5)	2.81 (d, 6.5)	2.85 (d, 6.5)	7.15 (d, 15.5)	7.23 (d, 15.5)
8	6.92 (m)	6.92 (m)	4.95 (m)	4.88 (m)	4.03 (m)	4.06 (m)	7.50 (dd, 15.0, 10.5)	7.45 (dd, 15.0, 10.0)
9	2.34 (m) 2.54 (m)	2.34 (m) 2.57 (m)	1.63 (m) 1.91 (m)	1.76 (m) 2.01 (m)	1.36 (m)	1.41 (m)	6.36 (dd, 15.0, 10.0)	6.36 (dd, 15.0, 10.0)
10	4.39 (m)	4.39 (m)	3.88 (m)	3.82 (m)	3.80 (br s)	3.80 (br s)	6.29 (dq, 14.5, 7.5)	6.29 (dq, 14.5, 7.5)
11	1.37 (d, 6.5)	1.37 (d, 6.5)	1.12 (d, 6.5)	1.13 (d, 6.5)	1.03 (m)	1.03 (m)	1.91 (d, 6.5)	1.92 (d, 6.0)
12	1.98 (m)	1.98 (m)			1.99 (m)	1.99 (m)		
13	0.91 (d, 7.0)	0.91 (d, 7.0)	2.11 (s)	2.11 (s)	0.92 (m)	0.92 (m)	2.28 (s)	2.28 (s)
14	0.69 (d, 7.0)	0.69 (d, 7.0)	1.91 (s)	1.91 (s)	0.68 (m)	0.68 (m)	1.90 (s)	1.88 (s)
10-OH			4.73 (d, 5.0)	4.69 (br s)	4.30 (br s)	4.34 (br s)		
8-OH					4.30 (br s)	4.30 (br s)		
6-OH							N.D. ^c^	N.D. ^c^

^a^ Chemical shifts were recorded in DMSO-*d*_6_. ^b^ Chemical shifts were recorded in CDCl_3_. ^c^ N.D.: Not detected.

**Table 2:** ^13^C NMR (125 MHz) Data for **1**–**5** (*δ* ppm).

	*δ*_C_, Type							
Compound	1 ^a^		2 ^a^	3 ^a^	4 ^a^		5 ^b^	
Position	a	b			a	b	a	b
2	169.6, C	167.9, C	163.5, C	163.4, C	177.6, C	177.7, C	166.0, C	172.1, C
3	102.9, C	103.0, C	105.6, C	105.6, C	102.1, C	102.1, C	101.6, C	103.5, C
4	196.4, C	199.6, C	173.0, C	173.1, C	195.5, C	195.5, C	188.6, C	182.4, C
5	64.4, CH	65.0, CH	127.7, C	127.8, C	65.3, CH	65.4, CH	129.7, C	128.4, C
6	165.1, C	166.7, C	185.9, C	185.8, C	192.7, C	192.7, C	177.5, C	174.2, C
7	119.5, CH	119.8, CH	41.6, CH_2_	41.1, CH_2_	46.1, CH_2_	46.1, CH_2_	119.7, CH	119.4, CH
8	143.2, CH	143.7, CH	80.5, CH	81.1, CH	66.2, CH	66.0, CH	145.6, CH	145.3, CH
9	30.4, CH_2_	30.3, CH_2_	43.1, CH_2_	42.9, CH_2_	46.6, CH_2_	46.7, CH_2_	131.2, CH	131.2, CH
10	73.3, CH	73.2, CH	61.8, CH	62.4, CH	63.4, CH	63.3, CH	142.7, CH	142.4, CH
11	20.0, CH_3_	19.0, CH_3_	24.2, CH_3_	23.3, CH_3_	24.9, CH_3_	25.0, CH_3_	19.1, CH_3_	19.1, CH_3_
12	29.8, CH	30.0, CH	125.7, C	125.6, C	29.9, CH	29.9, CH	125.7, C	124.7, C
13	19.0, CH_3_	20.0, CH_3_	19.7, CH_3_	19.6, CH_3_	20.0, CH_3_	20.0, CH_3_	19.0, CH_3_	18.4, CH_3_
14	15.8, CH_3_	15.6, CH_3_	21.6, CH_3_	21.6, CH_3_	15.9, CH_3_	15.9, CH_3_	21.1, CH_3_	21.1, CH_3_

^a^ Chemical shifts were recorded in DMSO-*d*_6_. ^b^ Chemical shifts were recorded in CDCl_3_.

**Table 3:** ^1^H (500 MHz) and ^13^C NMR (125 MHz) Data for **6** and **7** (in DMSO-*d*_6_, *δ* ppm).

Compound	6		Cladosporiumin G		7		Cladosporiumin E	
Position	*δ*_C_, Type	*δ*_H_ (*J* in Hz)	*δ*_C_	*δ*_H_ (*J* in Hz)	*δ*_C_, Type	*δ*_H_ (*J* in Hz)	*δ*_C_	*δ*_H_ (*J* in Hz)
1-NH		8.89 (br s)		6.68 (s)		10.06 (br s)		8.34 (s)
2	175.6, C		177.2		N.D. ^a^		172.5	
3	100.0, C		102.2		102.1, C		102.7	
4	195.4, C		196.5		N.D. ^a^		184.4	
5	66.9, CH	3.77 (br s)	64.4	3.24 (d, 1.7)	129.9, C		131.7	
6	173.2, C		181.7		N.D. ^a^		181.8	
7	122.8, CH	7.04 (d, 16.0)	132.4	7.57 (d, 15.5)	123.6, CH	7.19 (d, 15.5)	132.5	7.59 (d, 15.5)
8	147.6, CH	7.12 (m)	137.0	6.61 (m)	147.3, CH	7.12 (m)	137.1	6.61 (m)
9	42.9, CH_2_	2.36 (m)	42.5	2.18 (m) 2.25 (m)	43.0, CH_2_	2.36 (m)	42.5	2.18 (m) 2.26 (m)
10	65.7, CH	3.81 (m)	66.3	3.69 (m)	65.7, CH	3.88 (m)	66.3	3.70 (m)
11	23.9, CH_3_	1.09 (d, 6.0)	23.7	1.05 (d, 6.1)	23.9, CH_3_	1.09 (d, 6.0)	23.7	1.05 (d, 6.1)
12	30.2, CH	2.06 (m)	29.8	1.98 (m)	124.4, C		113.0	
13	19.5, CH_3_	0.96 (d, 7.0)	20.3	0.92 (d, 7.0)	18.8, CH_3_	2.16 (s)	18.2	2.14 (s)
14	16.2, CH_3_	0.74 (d, 6.5)	15.9	0.66 (d, 6.7)	21.5, CH_3_	1.82 (s)	21.3	1.68 (s)
10-OH		N.D. ^a^		4.62 (s)		N.D. ^a^		4.59 (s)
6-OH		N.D. ^a^		N.D. ^a^		N.D. ^a^		

^a^ N.D.: Not detected.

## Supplementary Materials

The following are available online at http://www.mdpi.com/1660-3397/16/11/448/s1, Figure S1–S47: 1D/2D NMR, HRESIMS and IR spectra of compounds **1**–**8**, Figure S48–S50: HPLC chromatograms of compound **4**, Figure S51: The ICP-AES analysis report of magnesium content in compound **4**, Figure S52–S55: HRESIMS spectrum of cladosporiumin E–H, Figure S56: Pictures of inhibition zones in the disc diffusion test, Table S1–S3: The stable conformers of compounds **1** and **2**.
