# Supplementary material for: Unstable Tetramic Acid Derivatives from the Deep-Sea-Derived Fungus Cladosporium sphaerospermum EIODSF 008
Source: Mar Drugs. 2018 Nov 15;16(11):448. doi: 10.3390/md16110448 (PMC6266709; doi:10.3390/md16110448)
Supplement: Supplementary file 1 [file marinedrugs-16-00448-s001.pdf]

Supporting information

Unstable tetramic acid derivatives from the deep-sea-derived fungus *Cladosporium sphaerospermum* EIODSF 008

Xiao Liang[1, 2], Zhong-Hui Huang[1, 2], Xuan Ma[1], Shu-Hua Qi[1, *]

[1] CAS Key Laboratory of Tropical Marine Bio-resources and Ecology/Guangdong Key Laboratory of Marine Materia Medica/RNAM Center for Marine Microbiology, South China Sea Institute of Oceanology,
Chinese Academy of Sciences, 164 West Xingang Road, Guangzhou 510301, Guangdong, China; liangxiao213@mails.ucas.ac.cn (X.L.); zhonghui23@hotmail.com (Z.-H.H.); maxuan@scsio.ac.cn (X.M.)

[2] University of Chinese Academy of Sciences, Beijing 100049, China

* Correspondence: shuhuaqi@scsio.ac.cn; Tel.: +86-20-8902-2112; Fax: +86-20-8445-8964

List of supporting information

Figure S1. The ^{1}H-NMR spectrum of cladosporiumin I (**1**) in DMSO-d_6 3
Figure S2. The ^{13}C NMR spectrum of cladosporiumin I (**1**) in DMSO-d_6 4
Figure S3. The HSQC spectrum of cladosporiumin I (**1**) in DMSO-d_6 5
Figure S4. The HMBC spectrum of cladosporiumin I (**1**) in DMSO-d_6 6
Figure S5. The ^{1}H-^{1}H COSY spectrum of cladosporiumin I (**1**) in DMSO-d_6 7
Figure S6. The (+)-HRESIMS spectrum of cladosporiumin I (**1**) 8
Figure S7. The ^{1}H-NMR spectrum of cladosporiumin J (**2**) in DMSO-d_6 9
Figure S8. The ^{13}C NMR spectrum of cladosporiumin J (**2**) in DMSO-d_6 10
Figure S9. The HSQC spectrum of cladosporiumin J (**2**) in DMSO-d_6 11
Figure S10. The HMBC spectrum of cladosporiumin J (**2**) in DMSO-d_6 12
Figure S11. The ^{1}H-^{1}H COSY spectrum of cladosporiumin J (**2**) in DMSO-d_6 13
Figure S12. The IR spectrum of cladosporiumin J (**2**) 14
Figure S13. The (+)-HRESIMS spectrum of cladosporiumin J (**2**) 15
Figure S14. The ^{1}H-NMR spectrum of cladosporiumin K (**3**) in DMSO-d_6 16
Figure S15. The ^{13}C NMR spectrum of cladosporiumin K (**3**) in DMSO-d_6 17
Figure S16. The HMBC spectrum of cladosporiumin K (**3**) in DMSO-d_6 18
Figure S17. The ^{1}H-^{1}H COSY spectrum of cladosporiumin K (**3**) in DMSO-d_6 19
Figure S18. The IR spectrum of cladosporiumin K (**3**) 20
Figure S19. The (+)-HRESIMS spectrum of cladosporiumin K (**3**) 21
Figure S20. The ^{1}H-NMR spectrum of cladosporiumin L (**4**) in DMSO-d_6 22
Figure S21. The ^{13}C NMR spectrum of cladosporiumin L (**4**) in DMSO-d_6 23
Figure S22. The HSQC spectrum of cladosporiumin L (**4**) in DMSO-d_6 24
Figure S23. The HMBC spectrum of cladosporiumin L (**4**) in DMSO-d_6 25
Figure S24. The ^{1}H-^{1}H COSY spectrum of cladosporiumin L (**4**) in DMSO-d_6 26

Figure S25. The IR spectrum of cladosporiumin L (**4**) 27
Figure S26. The (+)-HRESIMS spectrum of cladosporiumin L (**4**)........ 28
Figure S27. The ^{1}H-NMR spectrum of cladosporiumin M (**5**) in $CDCl_3$ 29
Figure S28. The ^{13}C NMR spectrum of cladosporiumin M (**5**) in $CDCl_3$........ 30
Figure S29. The HSQC spectrum of cladosporiumin M (**5**) in $CDCl_3$ 31
Figure S30. The HMBC spectrum of cladosporiumin M (**5**) in $CDCl_3$ 32
Figure S31. The ^{1}H-^{1}H COSY spectrum of cladosporiumin M (**5**) in $CDCl_3$........ 33
Figure S32. The IR spectrum of cladosporiumin M (**5**)........ 34
Figure S33. The (+)-HRESIMS spectrum of cladosporiumin M (**5**) 35
Figure S34. The ^{1}H-NMR spectrum of cladosporiumin N (**6**) in DMSO-d_6........ 36
Figure S35. The ^{13}C NMR spectrum of cladosporiumin N (**6**) in DMSO-d_6........ 37
Figure S36. The HSQC spectrum of cladosporiumin N (**6**) in DMSO-d_6........ 38
Figure S37. The HMBC spectrum of cladosporiumin N (**6**) in DMSO-d_6 39
Figure S38. The ^{1}H-^{1}H COSY spectrum of cladosporiumin N (**6**) in DMSO-d_6........ 40
Figure S39. The (+)-HRESIMS spectrum of cladosporiumin N (**6**)........ 41
Figure S40. The ^{1}H-NMR spectrum of cladosporiumin O (**7**) in DMSO-d_6........ 42
Figure S41. The ^{13}C NMR spectrum of cladosporiumin O (**7**) in DMSO-d_6........ 43
Figure S42. The HMBC spectrum of cladosporiumin O (**7**) in DMSO-d_6 44
Figure S43. The (+)-HRESIMS spectrum of cladosporiumin O (**7**)........ 45
Figure S44. The ^{1}H-NMR spectrum of cladodionen (**8**) in DMSO-d_6........ 46
Figure S45. The ^{13}C NMR spectrum of cladodionen (**8**) in DMSO-d_6........ 47
Figure S46. The IR spectrum of cladodionen (**8**)........ 48
Figure S47. The (+)-HRESIMS spectrum of cladodionen (**8**)........ 49
Figure S48. HPLC analysis of FDAA derivates of acidic hydrolysates of cladosporiumin L (**4**) **(**Column: YMC-Pack ODS-A column, 250×4.6 mml.D. , S-5 μm, 12 nm)........ 50
Figure S49. HPLC analysis of FDAA derivates of D-Val and L-Val (Column: YMC-Pack ODS-A column, 250×4.6 mml.D. , S-5 μm, 12 nm)........ 50
Figure S50. HPLC chromatograms of compound cladosporiumin L (**4**) before and after acid treatment 50
Figure S51. The ICP-AES analysis report of magnesium content in compound cladosporiumin L (**4**) 51
Figure S52. The (+)-HRESIMS spectrum of cladosporiumin E........ 52
Figure S53. The (+)-HRESIMS spectrum of cladosporiumin G........ 52
Figure S54. The (+)-HRESIMS spectrum of cladosporiumin F 53
Figure S55. The (+)-HRESIMS spectrum of cladosporiumin H........ 53
Figure S56. Pictures of inhibition zones in the disc diffusion test........ 54
Table S1. Free energies (*G*) and equilibrium populations (P) of stable conformers of **1a** and **1b** with *R* configuration at C-10 in CH_3OH 55
Table S2. Free energies (*G*) and equilibrium populations (P) of stable conformers of **1a** and **1b** with *S* configuration at C-10 in CH_3OH 56
Table S3. Free energies (*G*) and equilibrium populations (P) of stable conformers of **2** with *R* configuration at C-8 in CH_3OH 57

Figure S1. The ^{1}H-NMR spectrum of cladosporiumin I (**1**) in DMSO-d_6

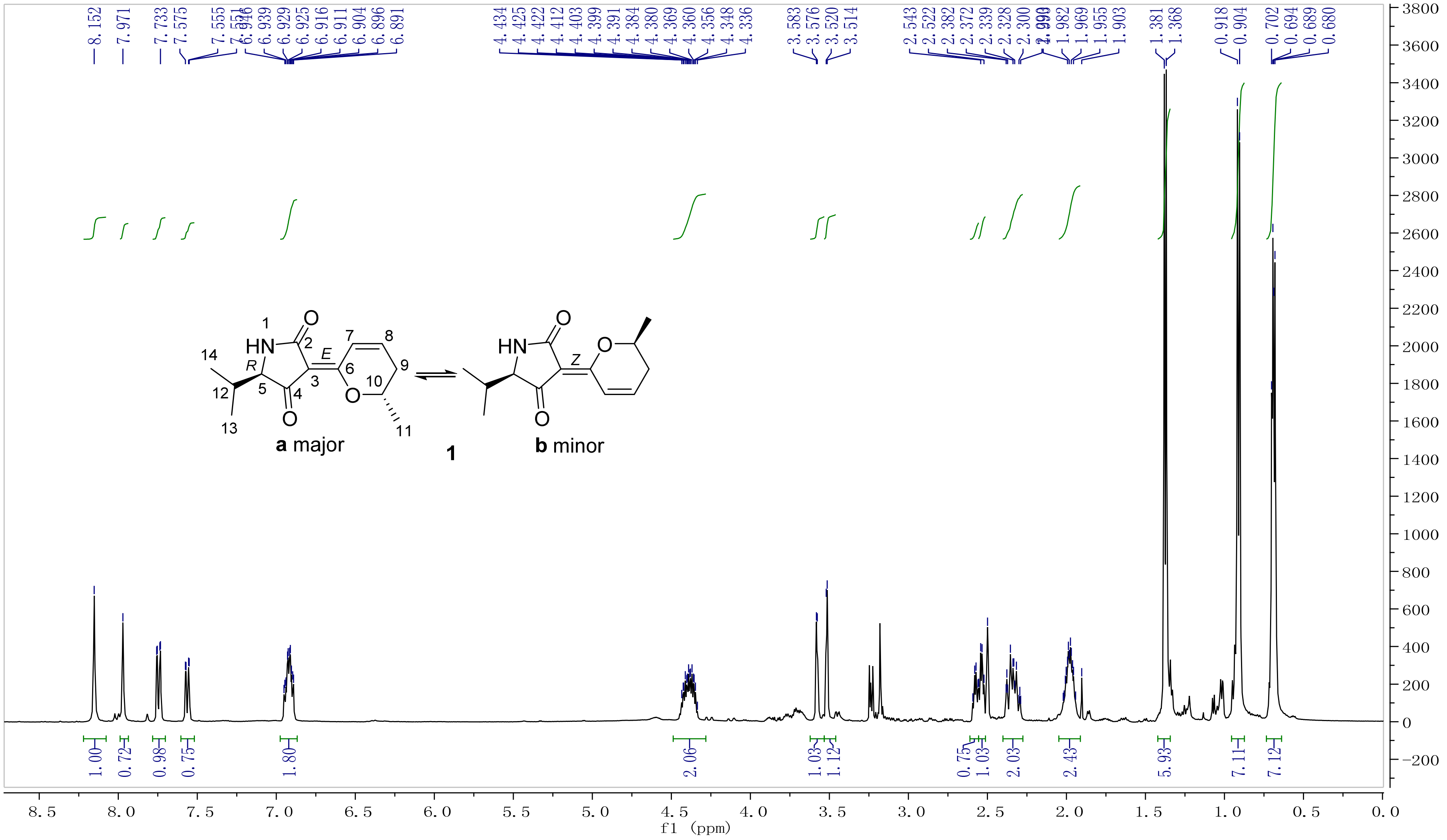

Figure S2. The ^{13}C NMR spectrum of cladosporiumin I (**1**) in DMSO-d_6

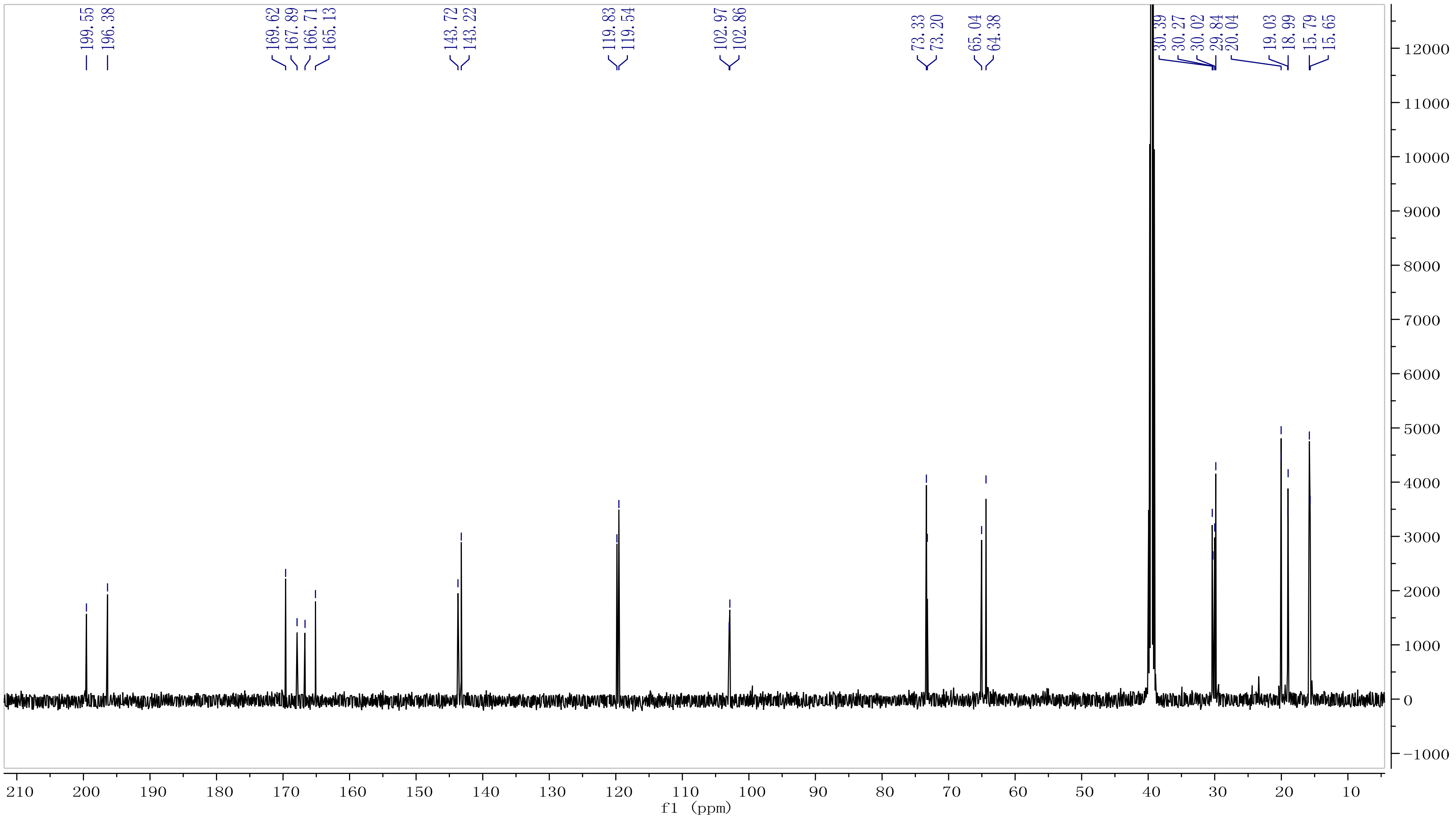

Figure S3. The HSQC spectrum of cladosporiumin I (**1**) in DMSO-d_6

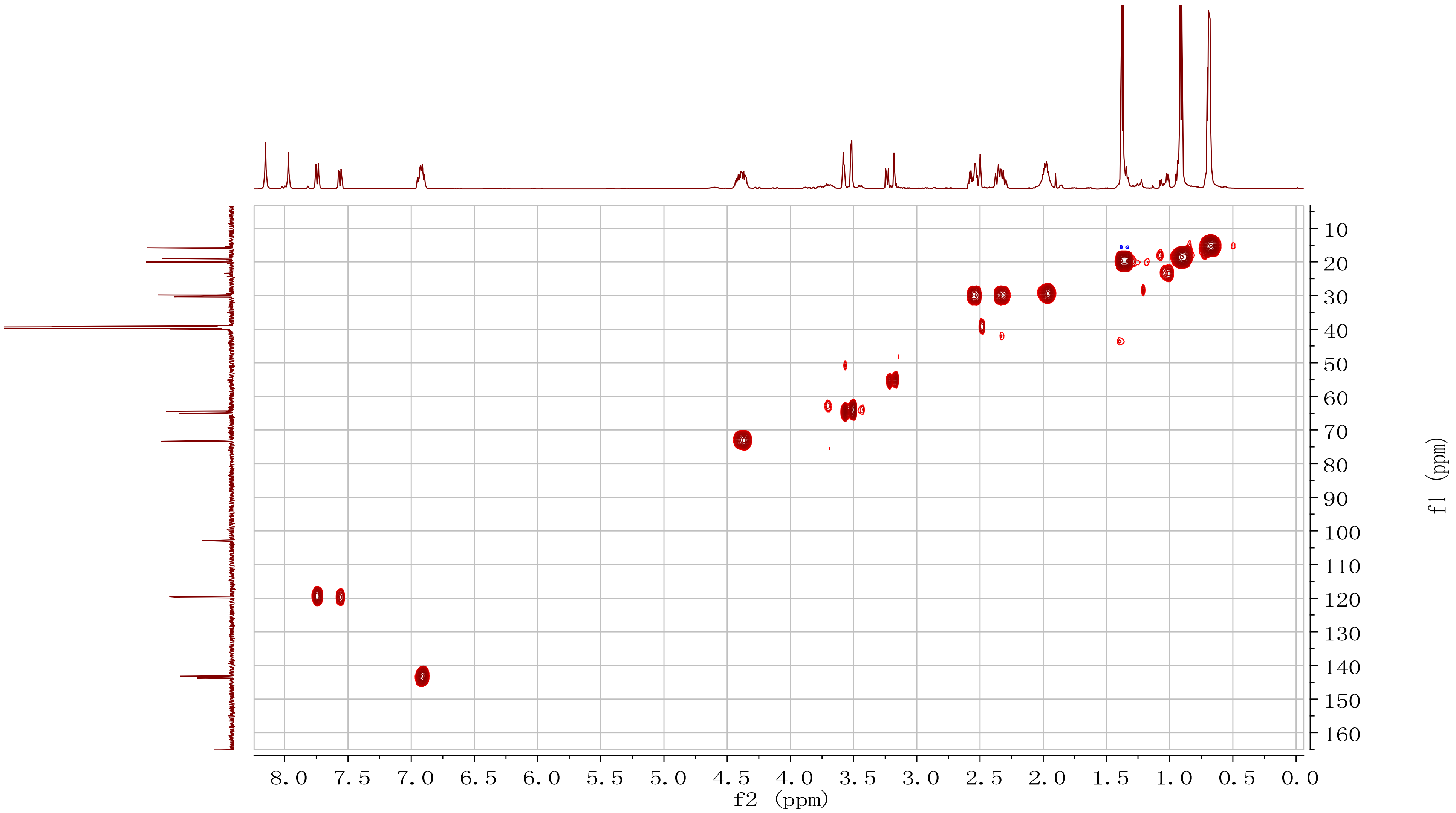

Figure S4. The HMBC spectrum of cladosporiumin I (**1**) in DMSO-d_6

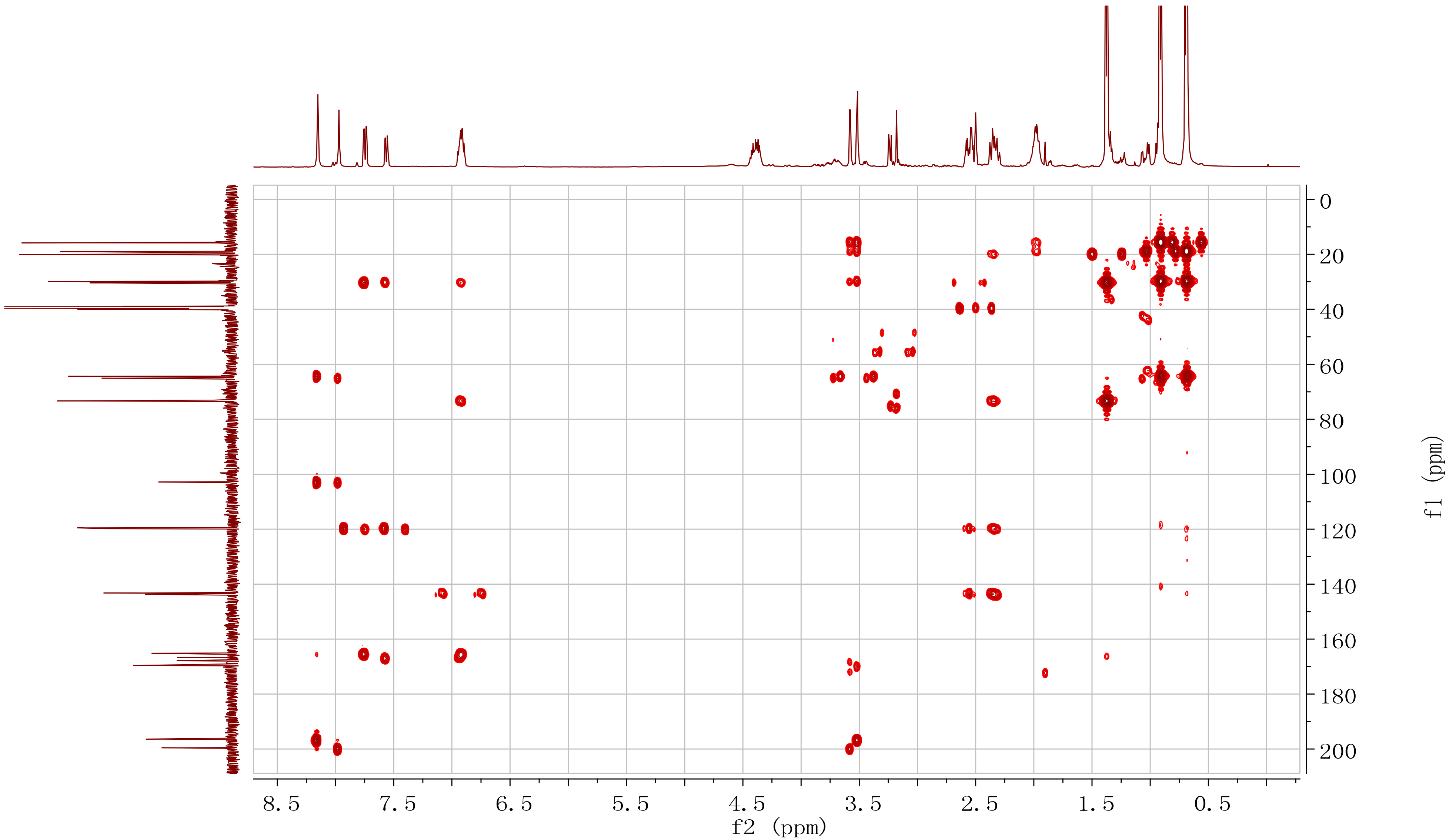

Figure S5. The ^{1}H-^{1}H COSY spectrum of cladosporiumin I (**1**) in DMSO-d_6

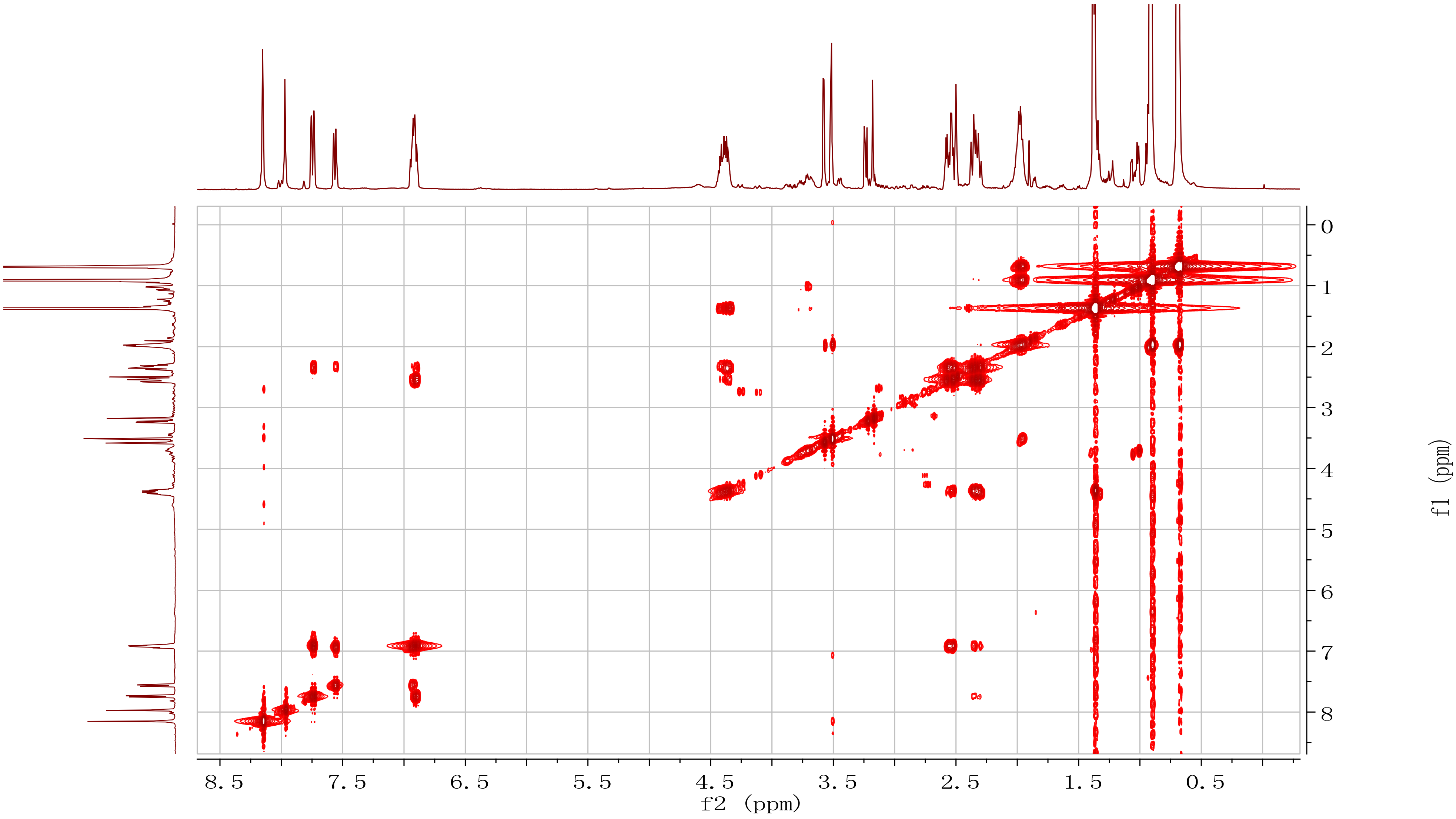

Figure S6. The (+)-HRESIMS spectrum of cladosporiumin I (**1**)

Mass Spectrum SmartFormula Report

Analysis Info

Analysis Name	D:\Data\MS\data\201604\liangxiao_LX-C-6_2_01_1564.d	Acquisition Date	4/1/2016 6:24:20 PM
Method	LC_Direct Infusion_pos_100-1000mz.m	Operator	SCSIO
Sample Name	liangxiao_LX-C-6	Instrument / Ser#	maXis 29
Comment			

Acquisition Parameter

Source Type	ESI	Ion Polarity	Positive	Set Nebulizer	0.4 Bar
Focus	Active	Set Capillary	4500 V	Set Dry Heater	180 °C
Scan Begin	100 m/z	Set End Plate Offset	-500 V	Set Dry Gas	4.0 l/min
Scan End	2000 m/z	Set Collision Cell RF	800.0 Vpp	Set Divert Valve	Waste

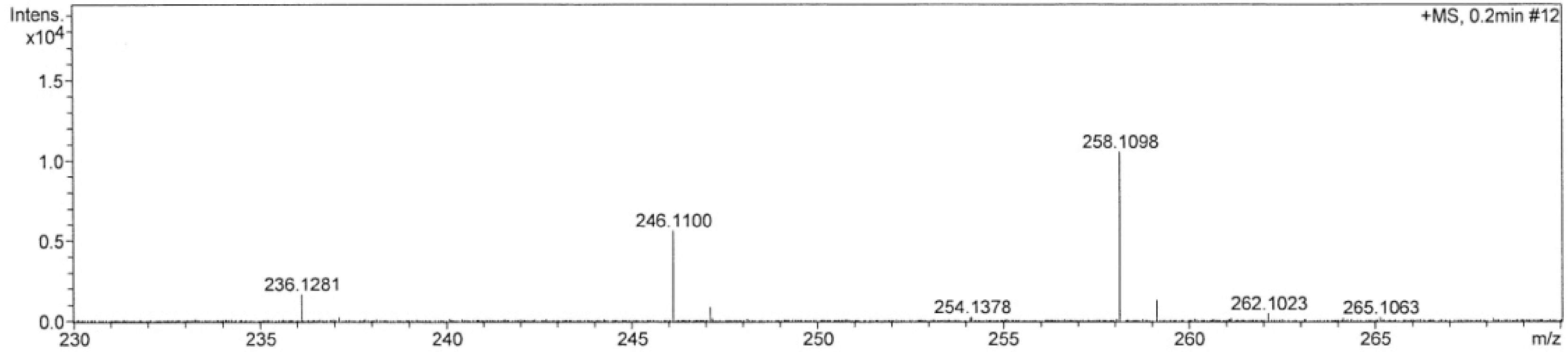

Meas. m/z	#	Formula	Score	m/z	err [ppm]	err [mDa]	mSigma	rdb	e⁻ Conf	N-Rule
236.1281	1	C 13 H 18 N O 3	100.00	236.1281	-0.1	-0.0	28.9	5.5	even	ok
258.1098	1	C 13 H 17 N Na O 3	100.00	258.1101	1.0	0.3	10.2	5.5	even	ok

Figure S7. The ^{1}H-NMR spectrum of cladosporiumin J (**2**) in DMSO-d_6

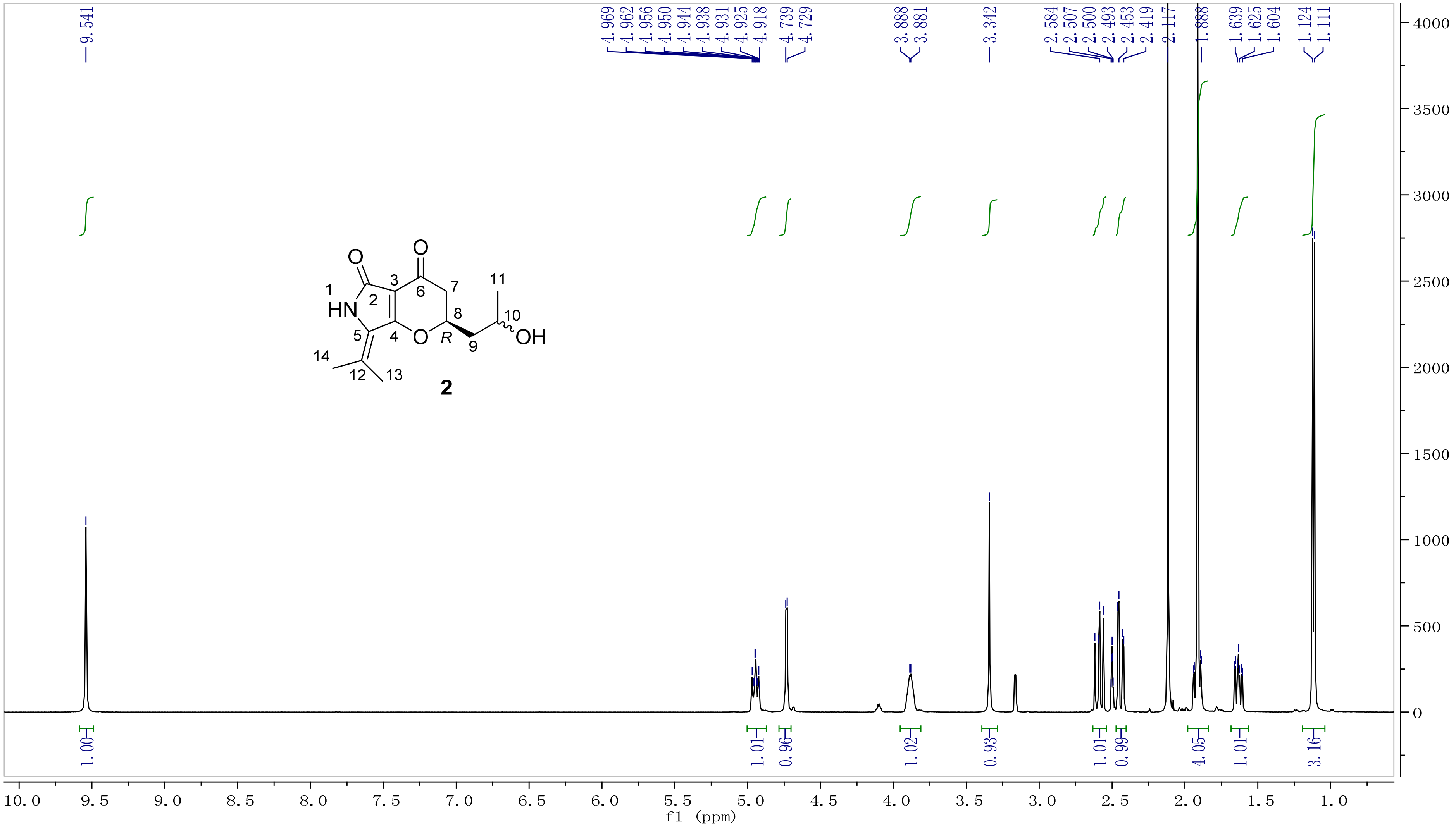

Figure S8. The ^{13}C NMR spectrum of cladosporiumin J (**2**) in DMSO-d_6

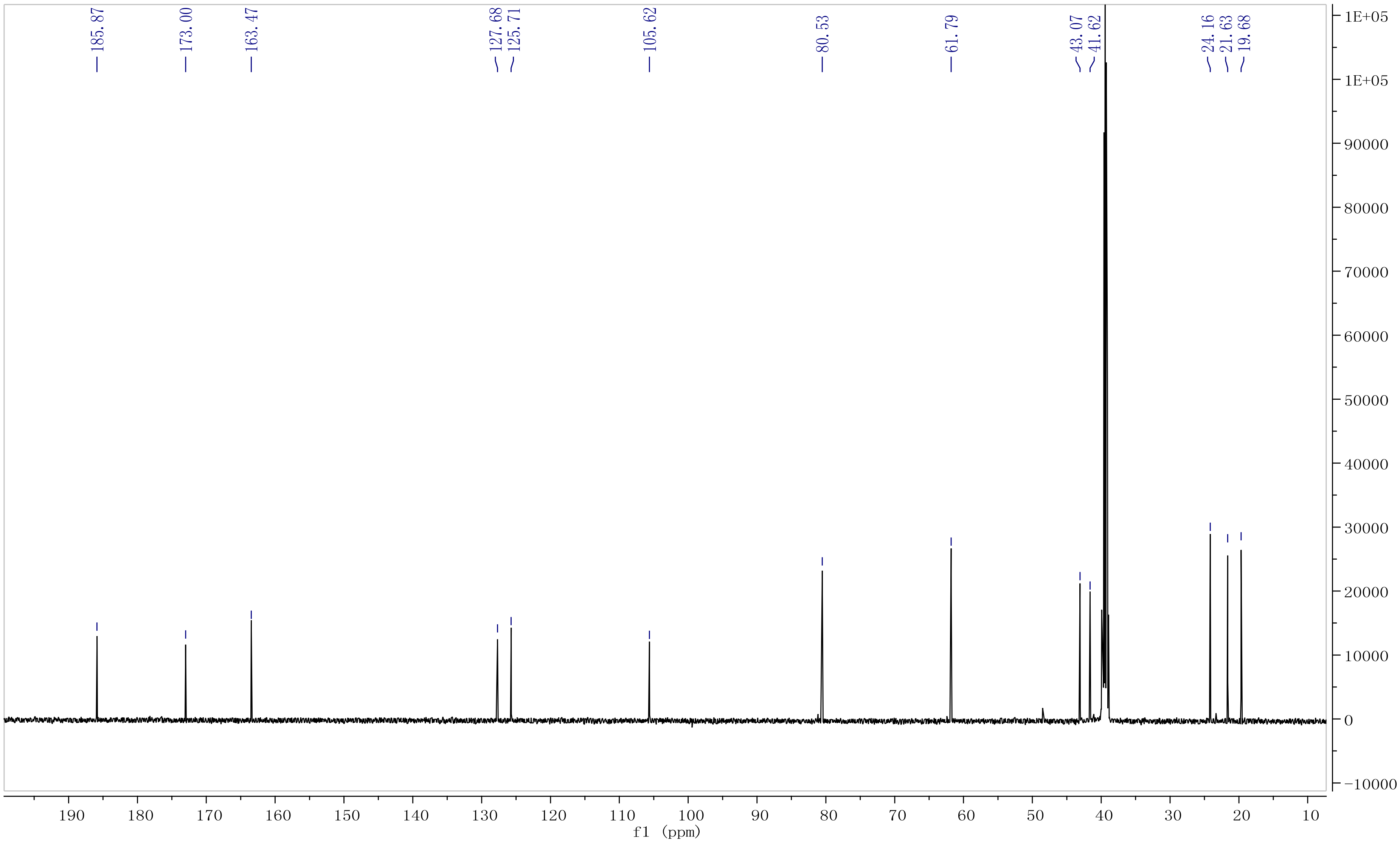

Figure S9. The HSQC spectrum of cladosporiumin J (**2**) in DMSO-d_6

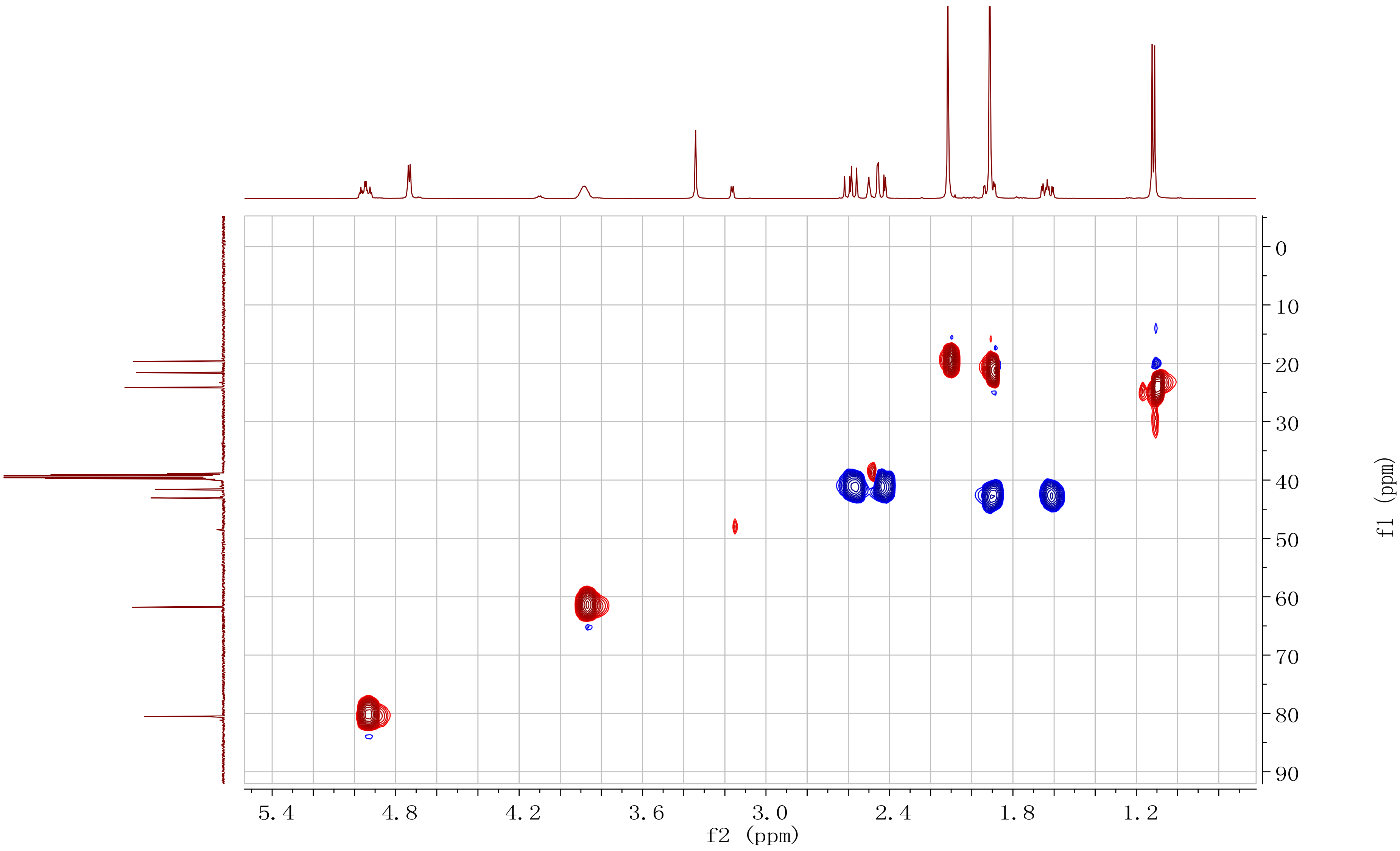

Figure S10. The HMBC spectrum of cladosporiumin J (**2**) in DMSO-d_6

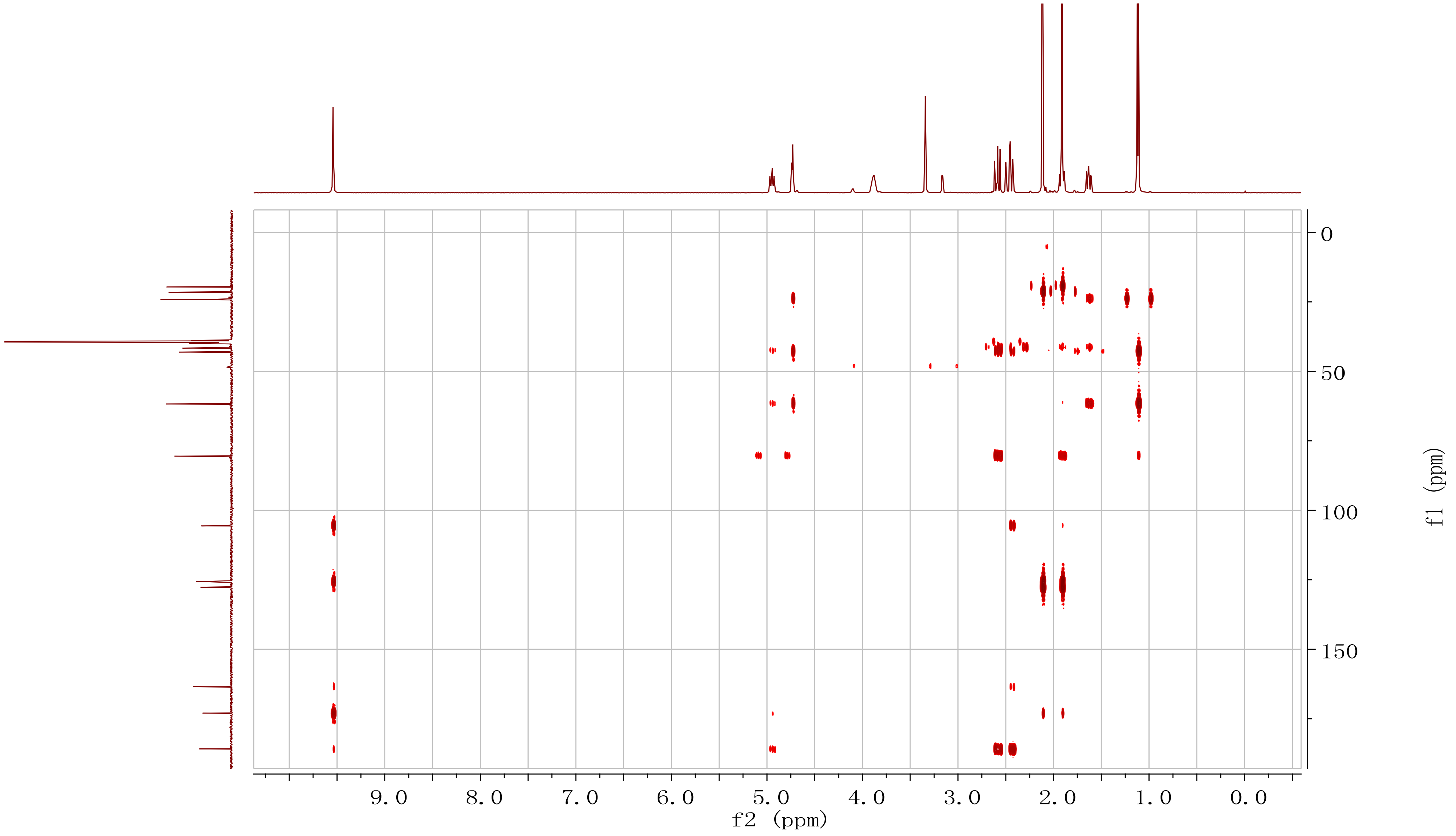

Figure S11. The ^{1}H-^{1}H COSY spectrum of cladosporiumin J (**2**) in DMSO-d_6

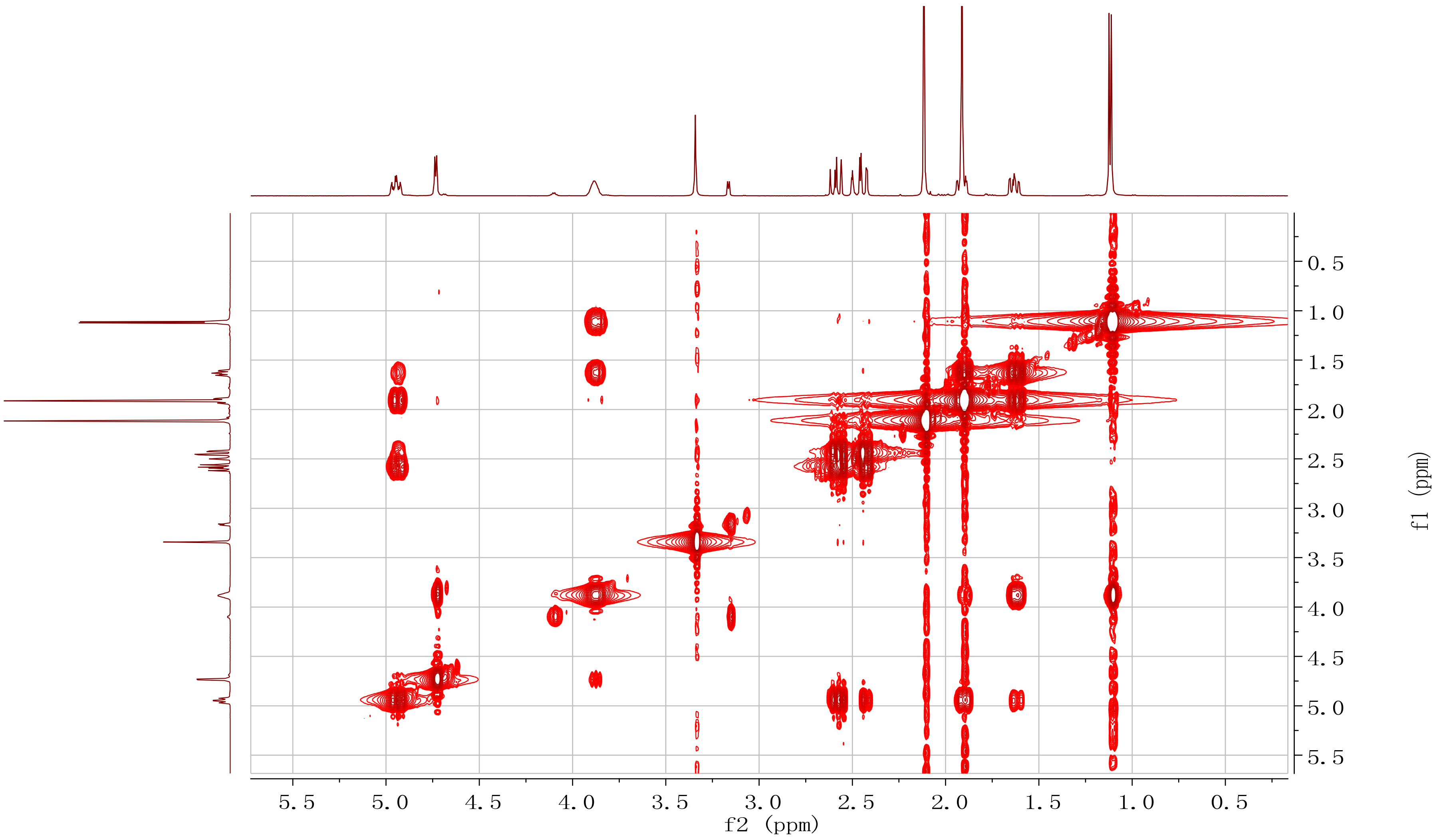

Figure S12. The IR spectrum of cladosporiumin J (**2**)

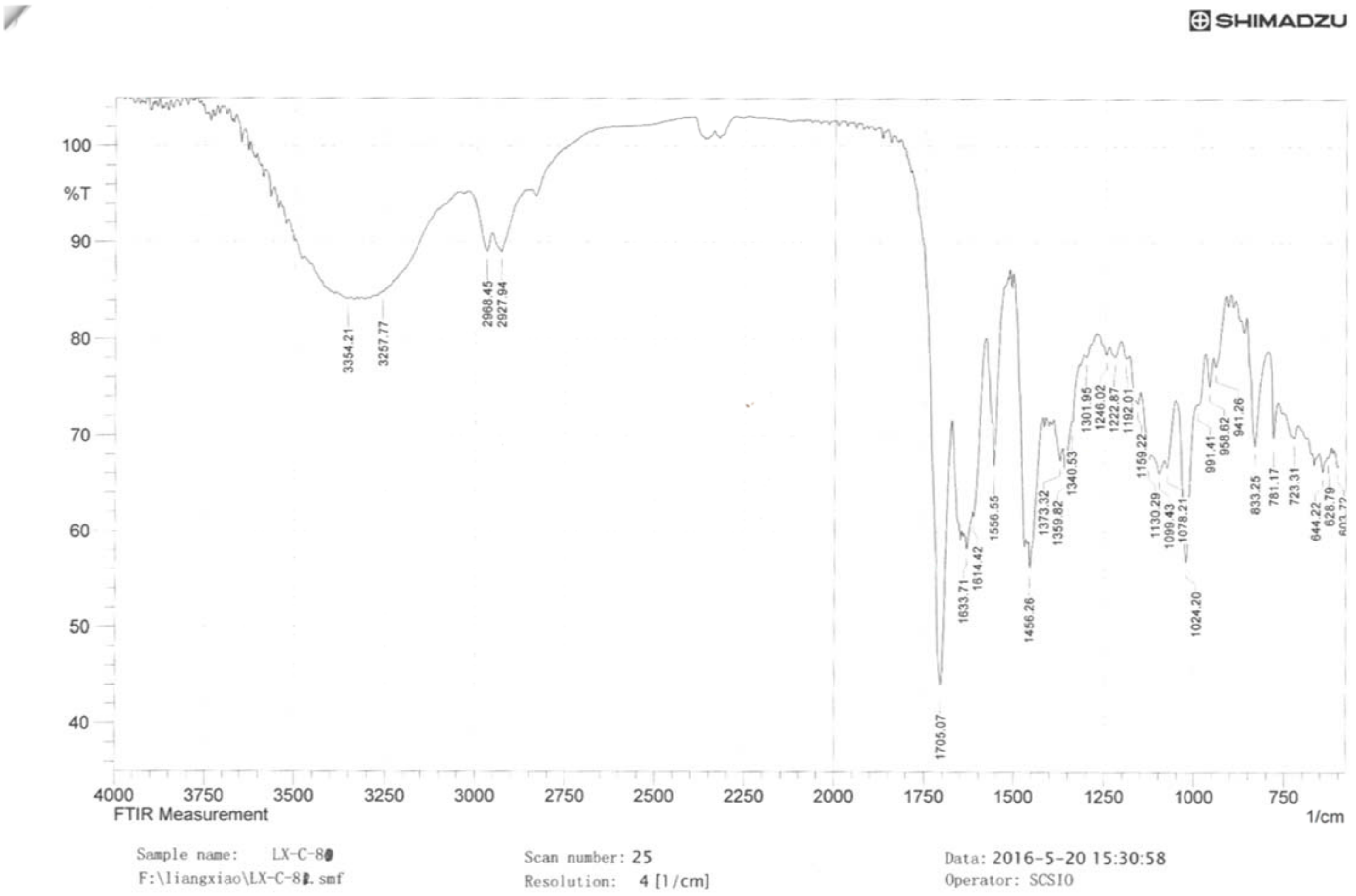

Figure S13. The (+)-HRESIMS spectrum of cladosporiumin J (**2**)

Mass Spectrum SmartFormula Report

Analysis Info

Analysis Name	D:\Data\MS\data\201604\liangxiao_LX-C-8_4_01_1566.d	Acquisition Date	4/1/2016 6:31:09 PM
Method	LC_Direct Infusion_pos_100-1000mz.m	Operator	SCSIO
Sample Name	liangxiao_LX-C-8	Instrument / Ser#	maXis 29
Comment			

Acquisition Parameter

Source Type	ESI	Ion Polarity	Positive	Set Nebulizer	0.4 Bar
Focus	Active	Set Capillary	4500 V	Set Dry Heater	180 °C
Scan Begin	100 m/z	Set End Plate Offset	-500 V	Set Dry Gas	4.0 l/min
Scan End	2000 m/z	Set Collision Cell RF	800.0 Vpp	Set Divert Valve	Waste

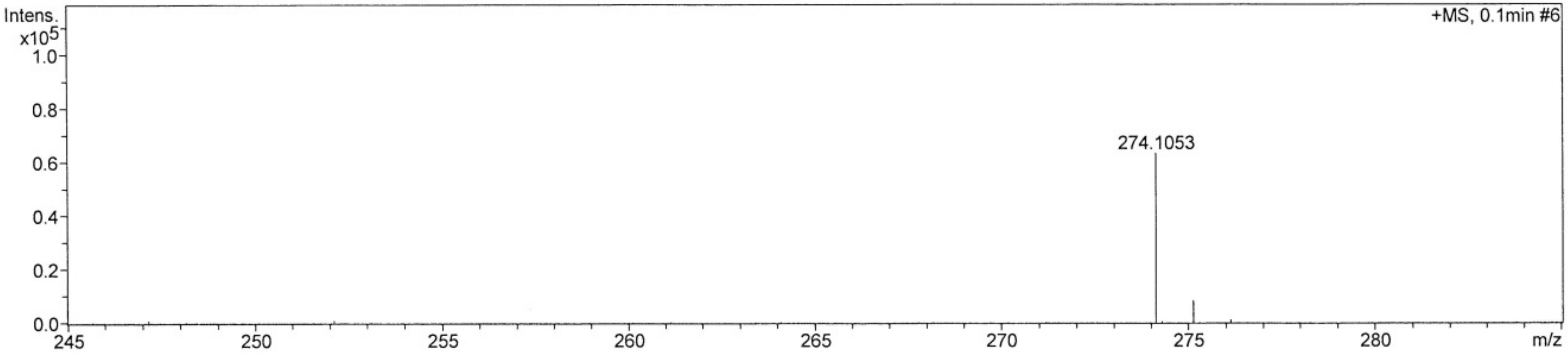

Meas. m/z	#	Formula	Score	m/z	err [ppm]	err [mDa]	mSigma	rdb	e⁻ Conf	N-Rule
252.1230	1	C 13 H 18 N O 4	100.00	252.1230	0.3	0.1	10.8	5.5	even	ok
274.1053	1	C 13 H 17 N Na O 4	100.00	274.1050	-1.2	-0.3	8.4	5.5	even	ok
525.2203	1	C 26 H 34 N 2 Na O 8	100.00	525.2207	0.8	0.4	5.4	10.5	even	ok

Figure S14. The ^{1}H-NMR spectrum of cladosporiumin K (**3**) in DMSO-d_6

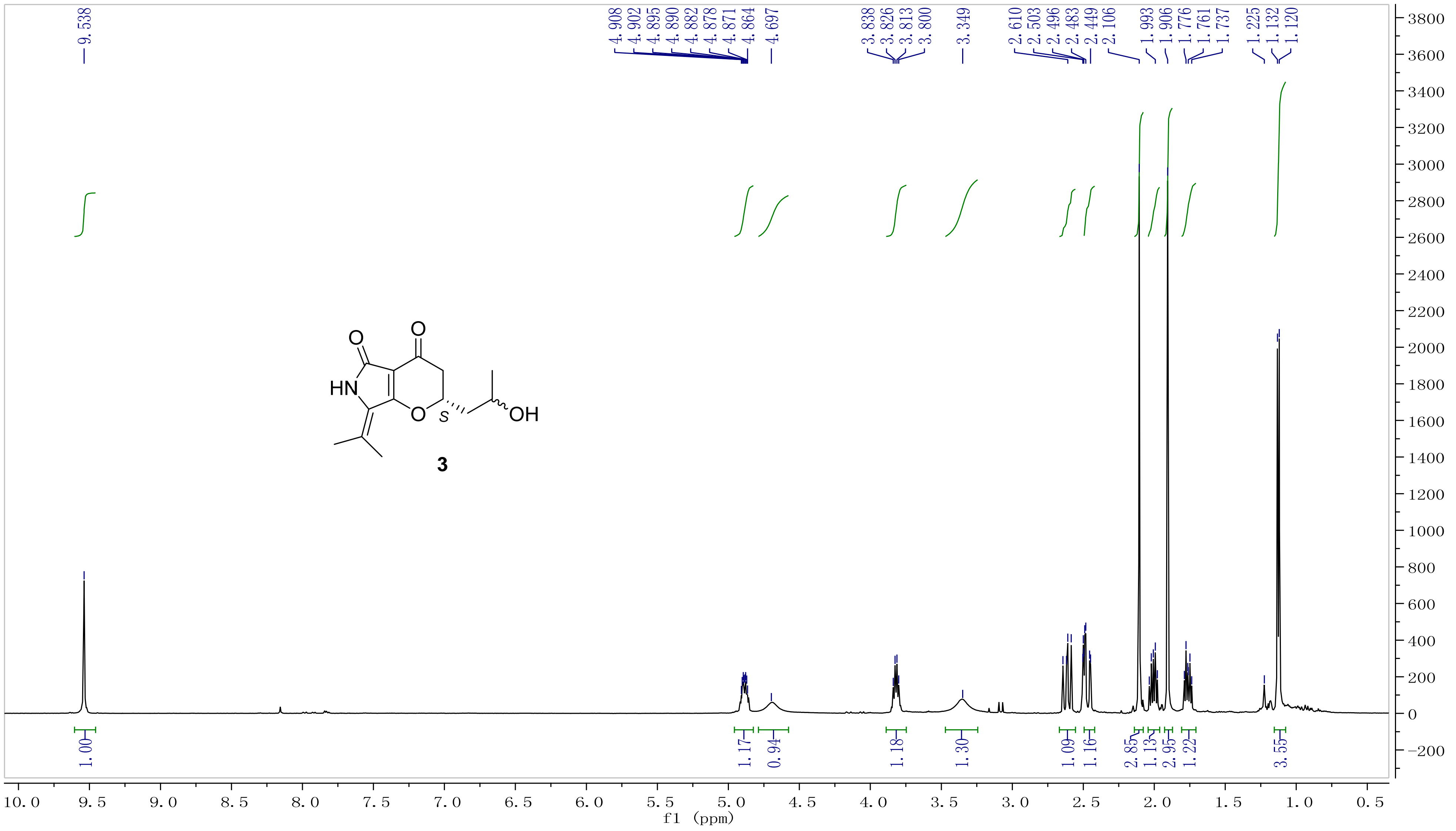

Figure S15. The ^{13}C NMR spectrum of cladosporiumin K (**3**) in DMSO-d_6

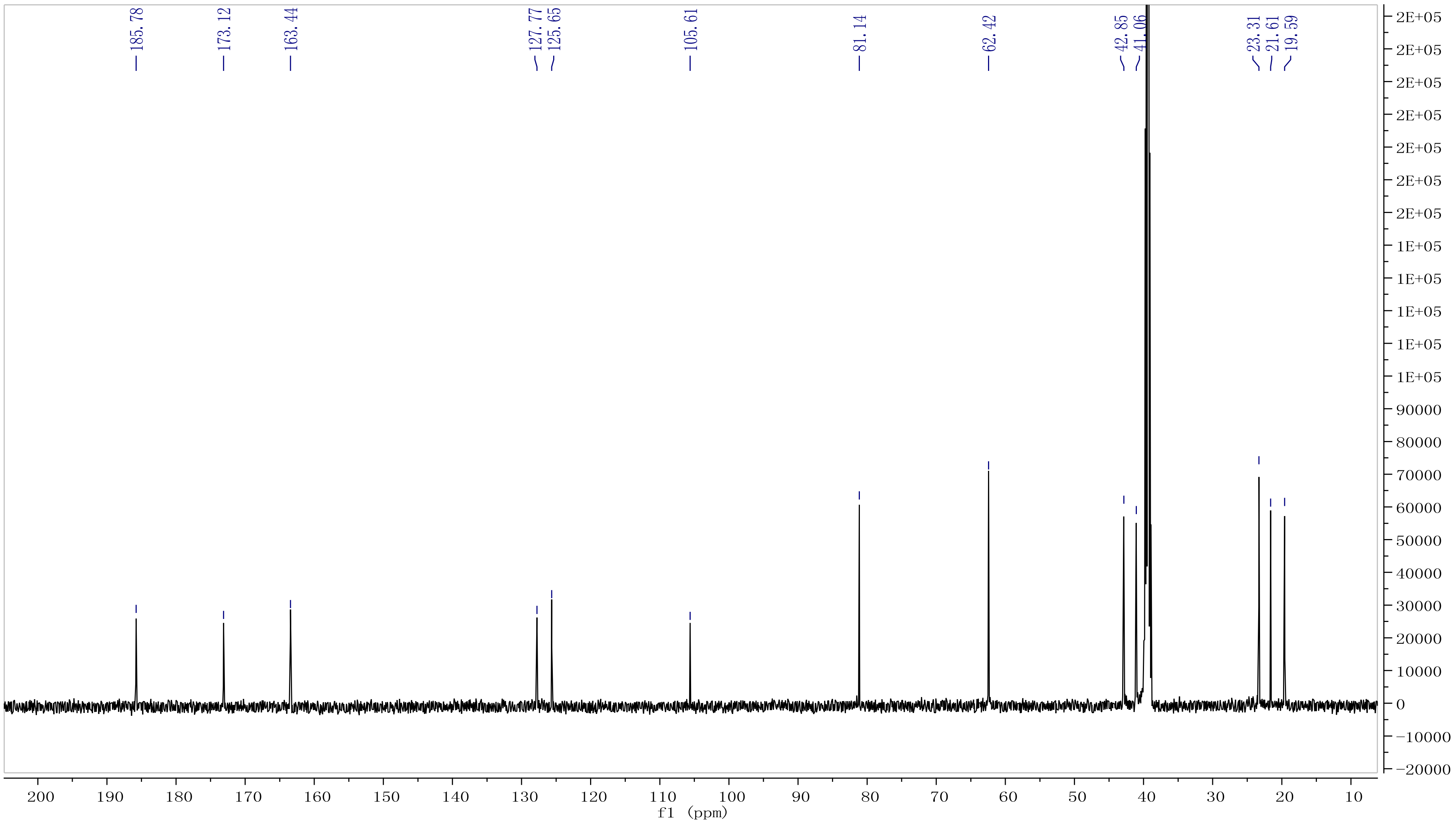

Figure S16. The HMBC spectrum of cladosporiumin K (**3**) in DMSO-d_6

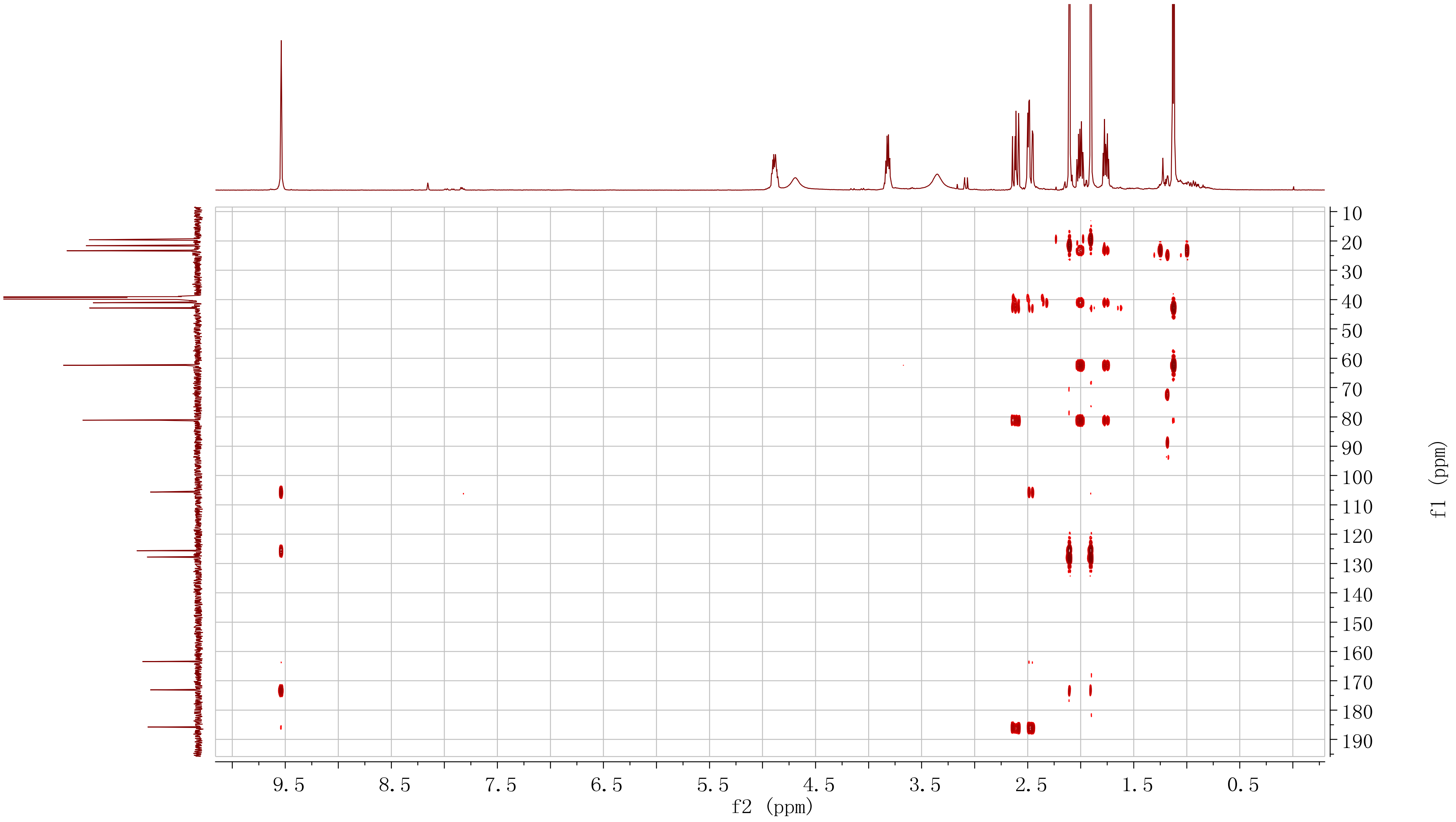

Figure S17. The ^{1}H-^{1}H COSY spectrum of cladosporiumin K (**3**) in DMSO-d_6

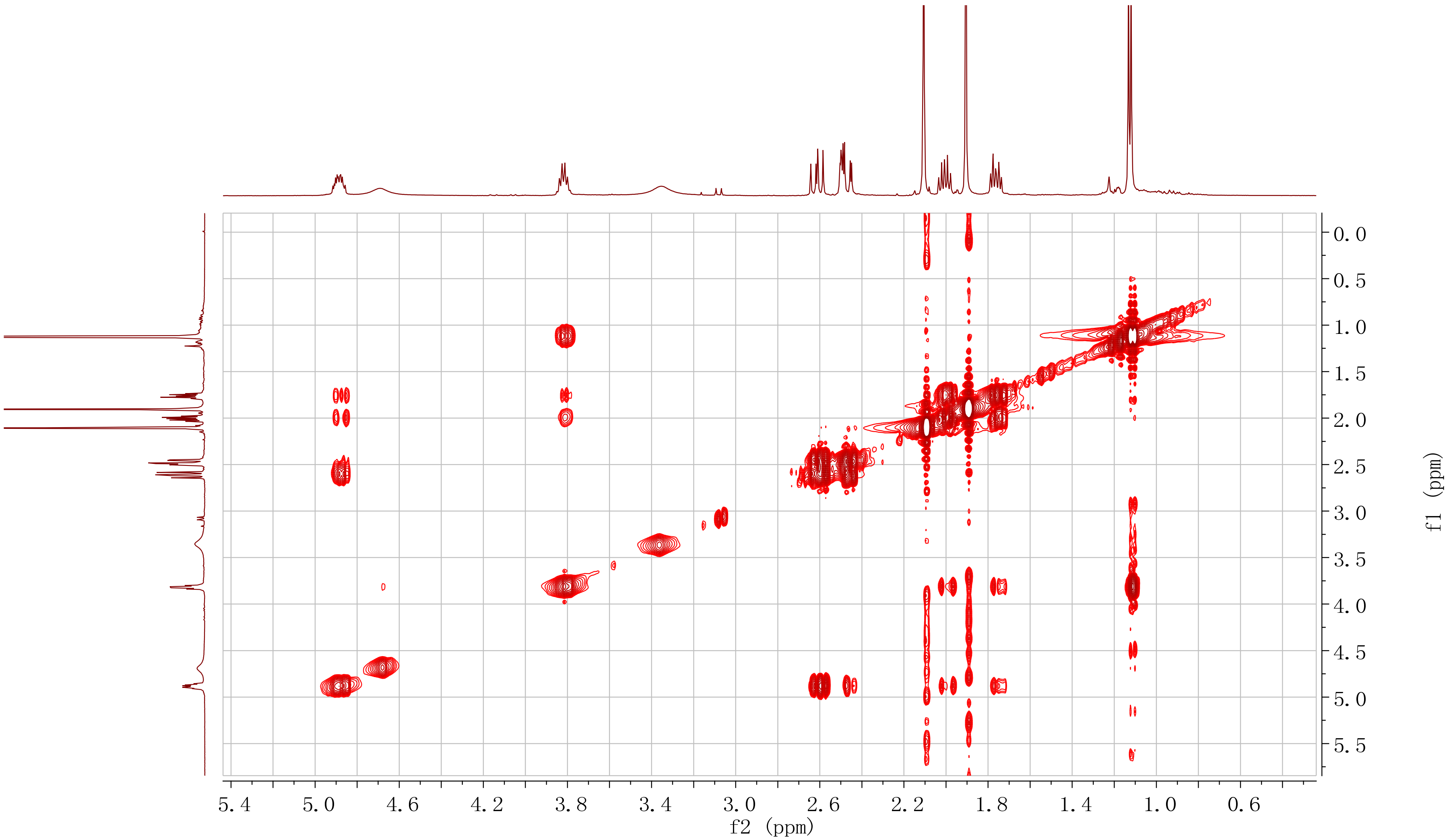

Figure S18. The IR spectrum of cladosporiumin K (**3**)

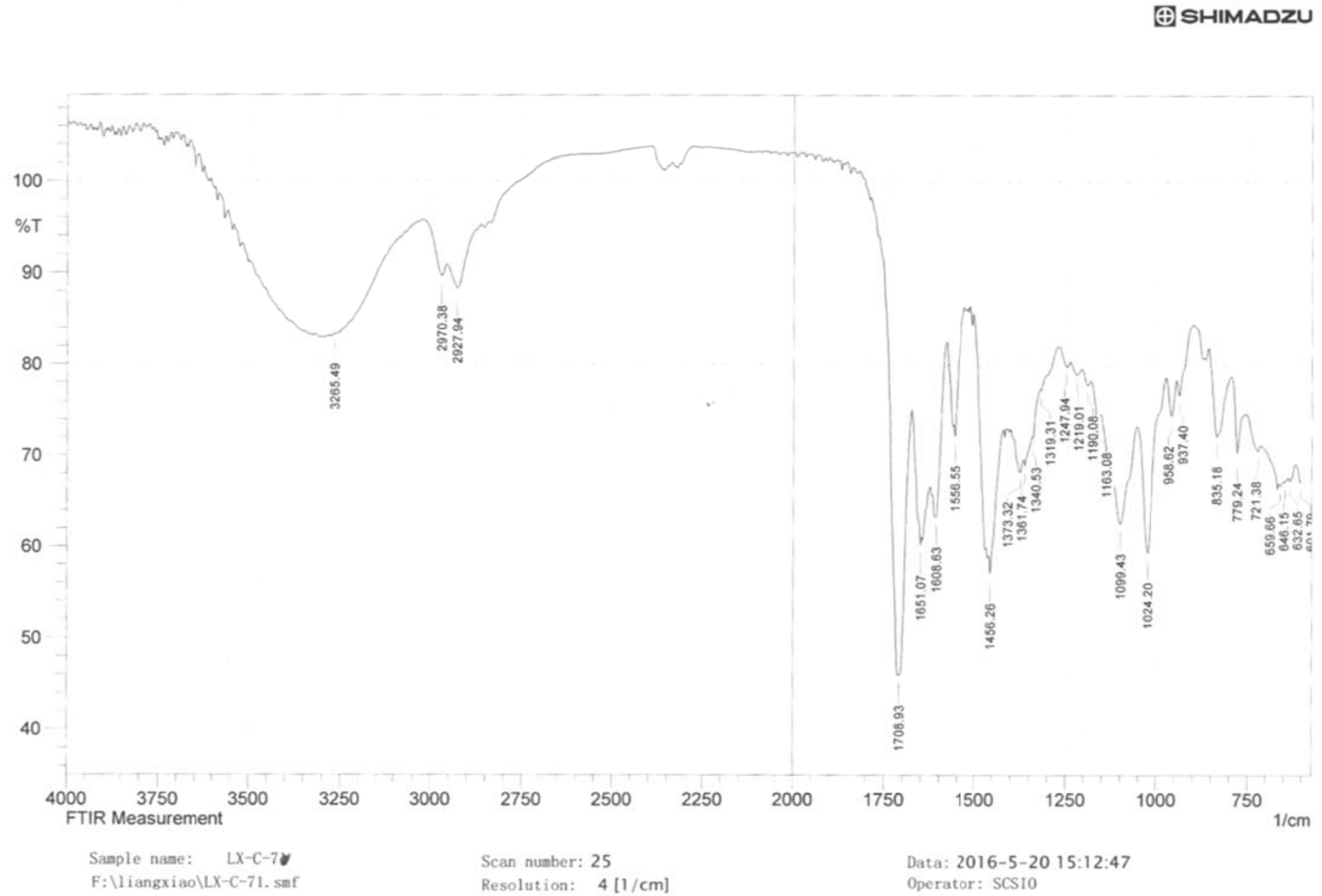

Figure S19. The (+)-HRESIMS spectrum of cladosporiumin K (**3**)

Mass Spectrum SmartFormula Report

Analysis Info

Analysis Name	D:\Data\MS\data\201604\liangxiao_LX-C-7_3_01_1568.d	Acquisition Date	4/1/2016 6:45:29 PM
Method	LC_Direct Infusion_pos_100-1000mz.m	Operator	SCSIO
Sample Name	liangxiao_LX-C-7	Instrument / Ser#	maXis 29
Comment			

Acquisition Parameter

Source Type	ESI	Ion Polarity	Positive	Set Nebulizer	0.4 Bar
Focus	Active	Set Capillary	4500 V	Set Dry Heater	180 °C
Scan Begin	100 m/z	Set End Plate Offset	-500 V	Set Dry Gas	4.0 l/min
Scan End	2000 m/z	Set Collision Cell RF	800.0 Vpp	Set Divert Valve	Waste

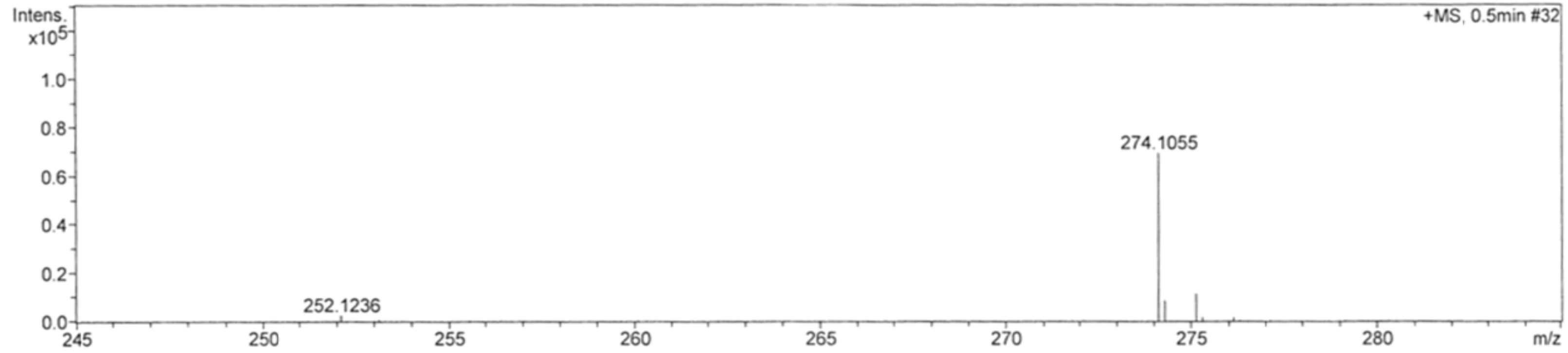

Meas. m/z	#	Formula	Score	m/z	err [ppm]	err [mDa]	mSigma	rdb	e⁻ Conf	N-Rule
252.1236	1	C 13 H 18 N O 4	78.59	252.1230	-2.3	-0.6	37.0	5.5	even	ok
274.1055	1	C 13 H 17 N Na O 4	100.00	274.1050	-2.0	-0.5	11.9	5.5	even	ok
525.2215	1	C 26 H 34 N 2 Na O 8	100.00	525.2207	-1.5	-0.8	7.4	10.5	even	ok

Figure S20. The ^{1}H-NMR spectrum of cladosporiumin L (**4**) in DMSO-d_6

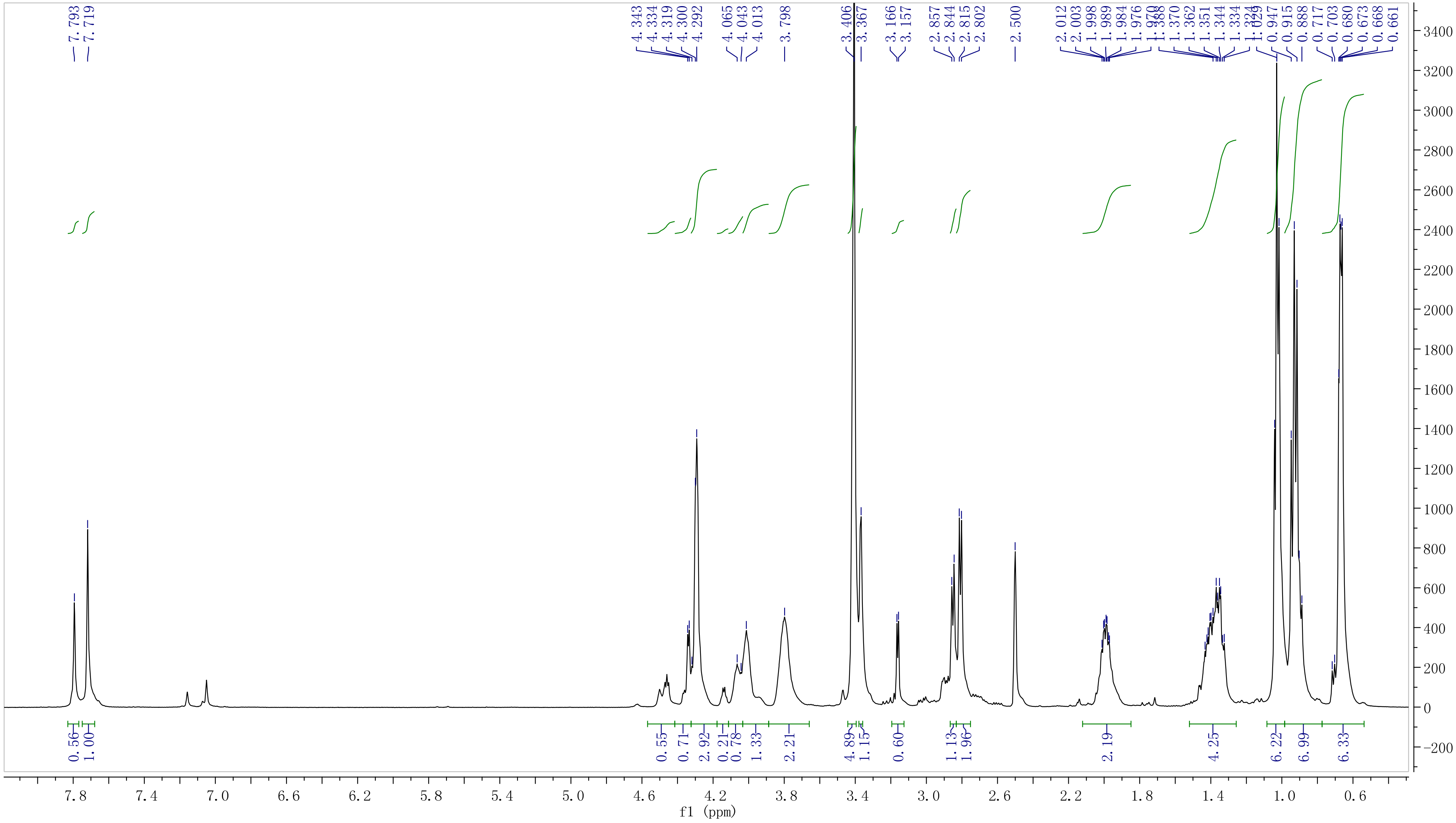

Figure S21. The ^{13}C NMR spectrum of cladosporiumin L (**4**) in DMSO-d_6

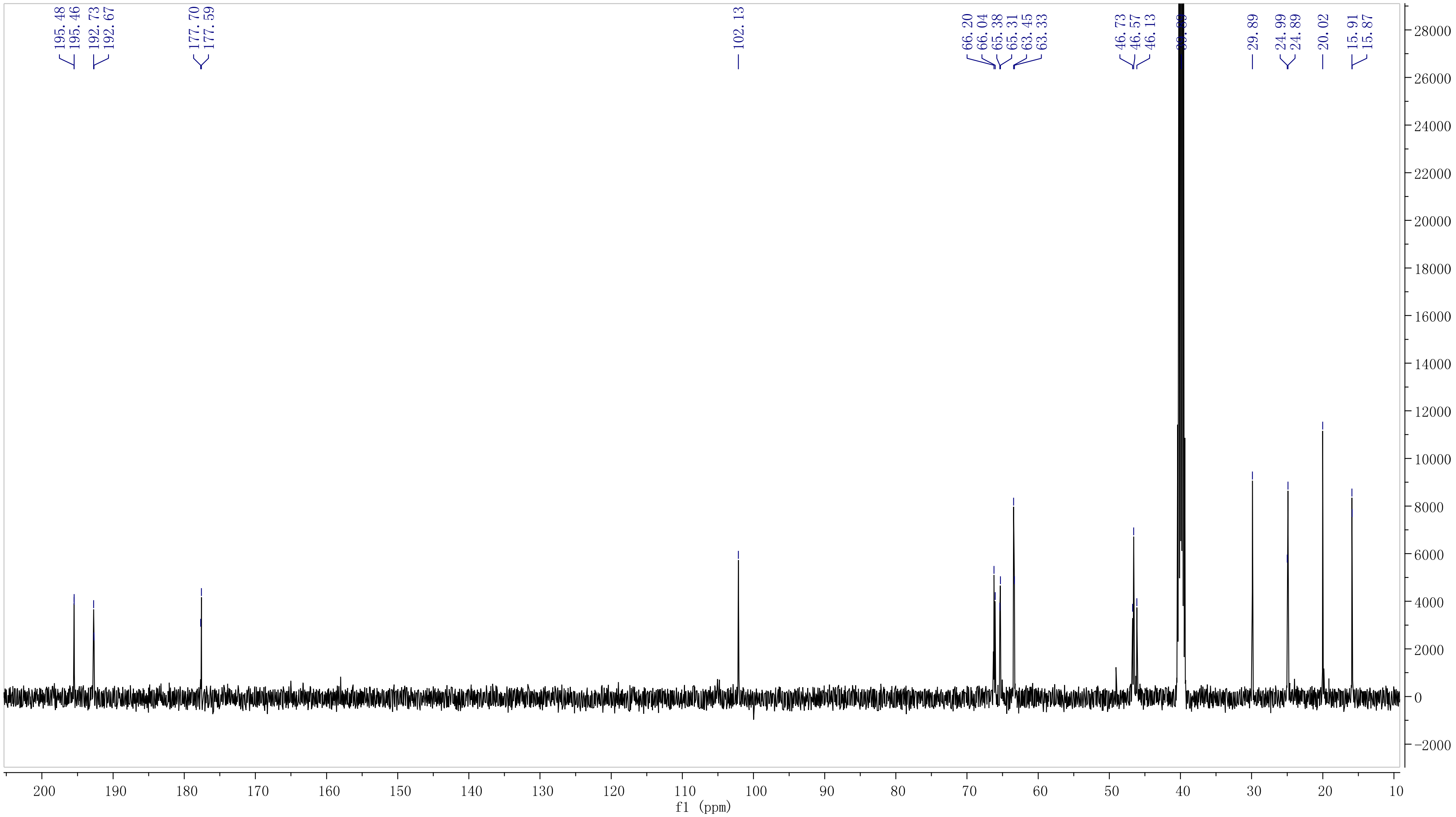

Figure S22. The HSQC spectrum of cladosporiumin L (**4**) in DMSO-d_6

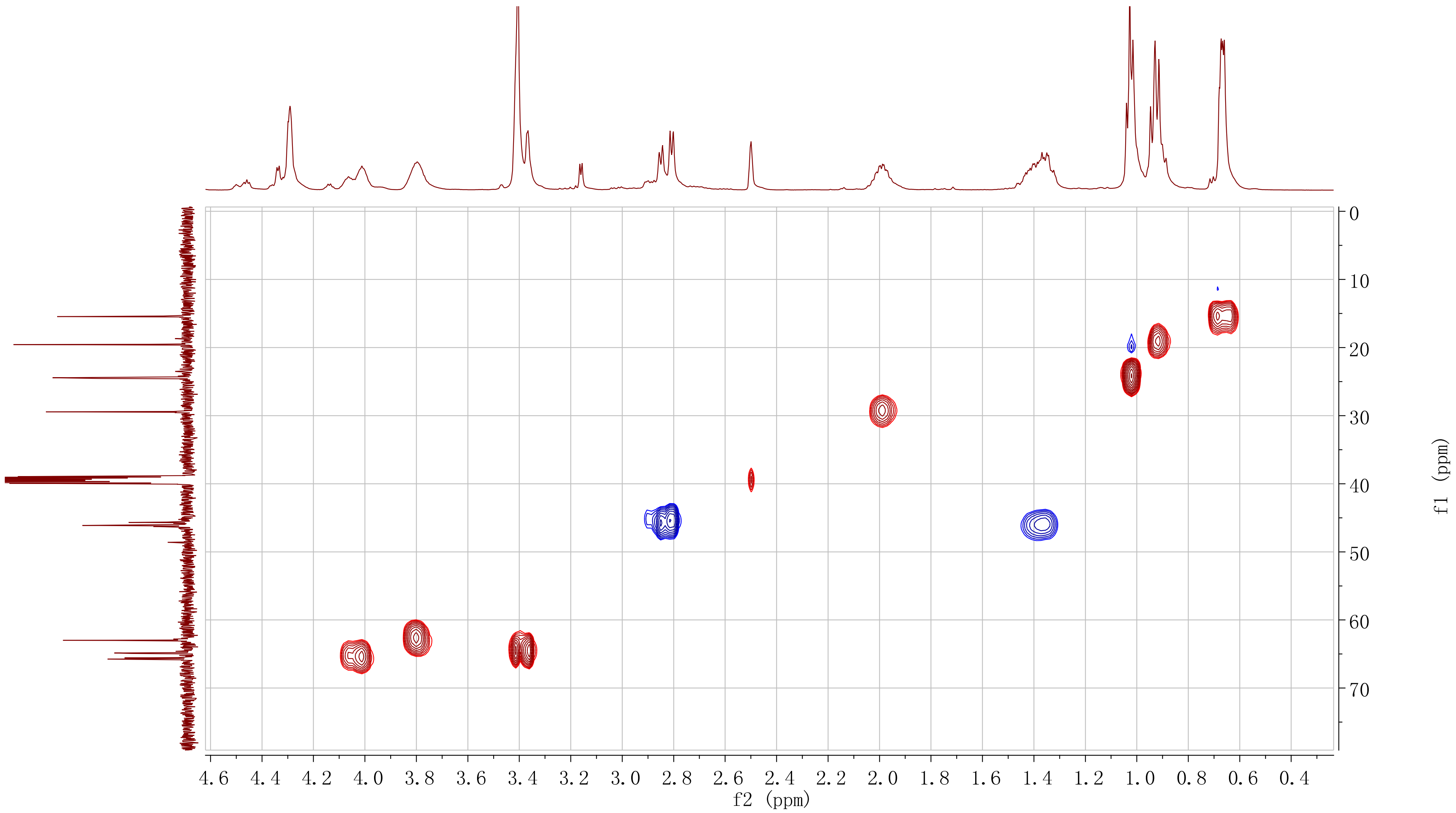

Figure S23. The HMBC spectrum of cladosporiumin L (**4**) in DMSO-d_6

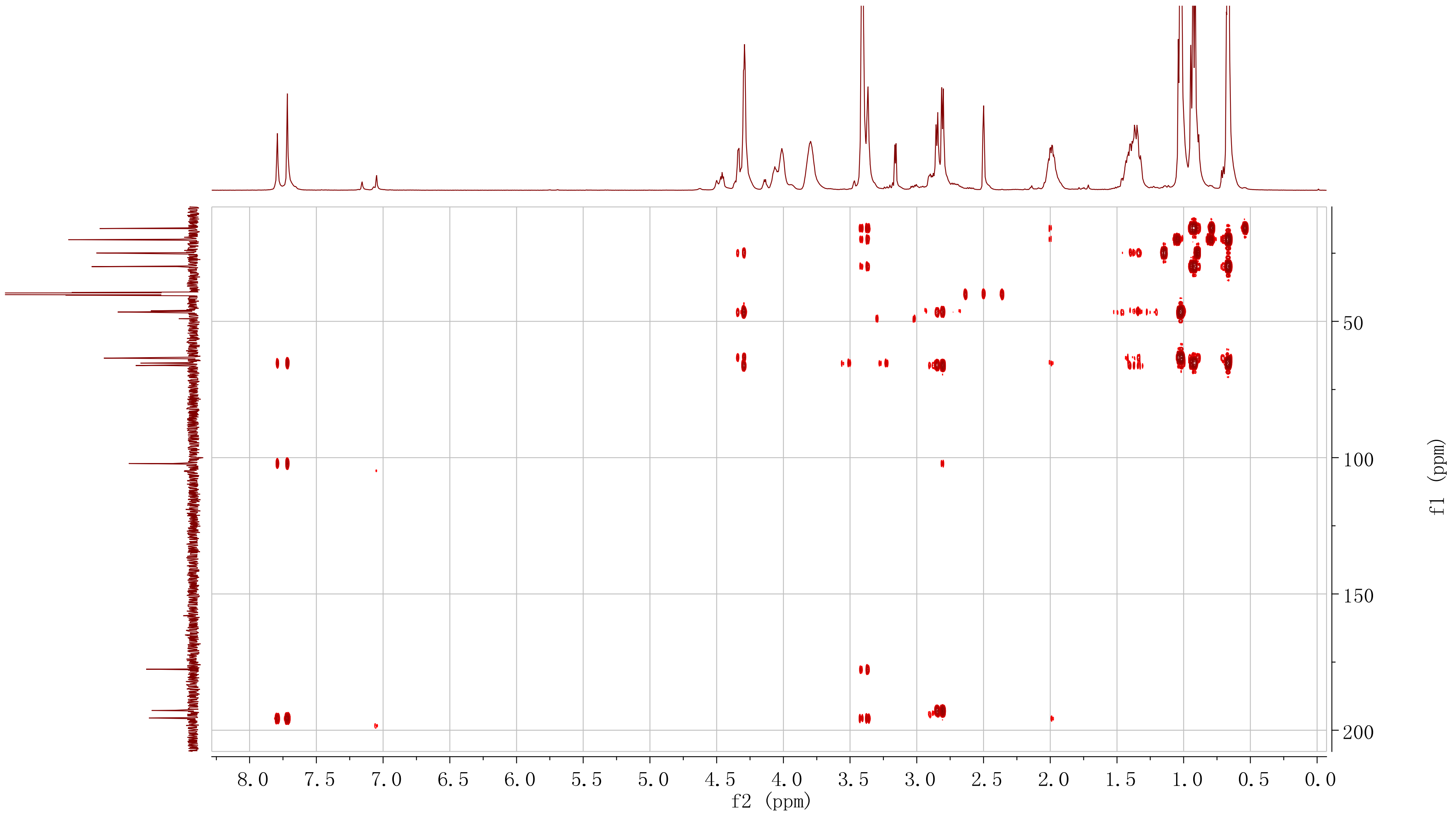

Figure S24. The ^{1}H-^{1}H COSY spectrum of cladosporiumin L (**4**) in DMSO-d_6

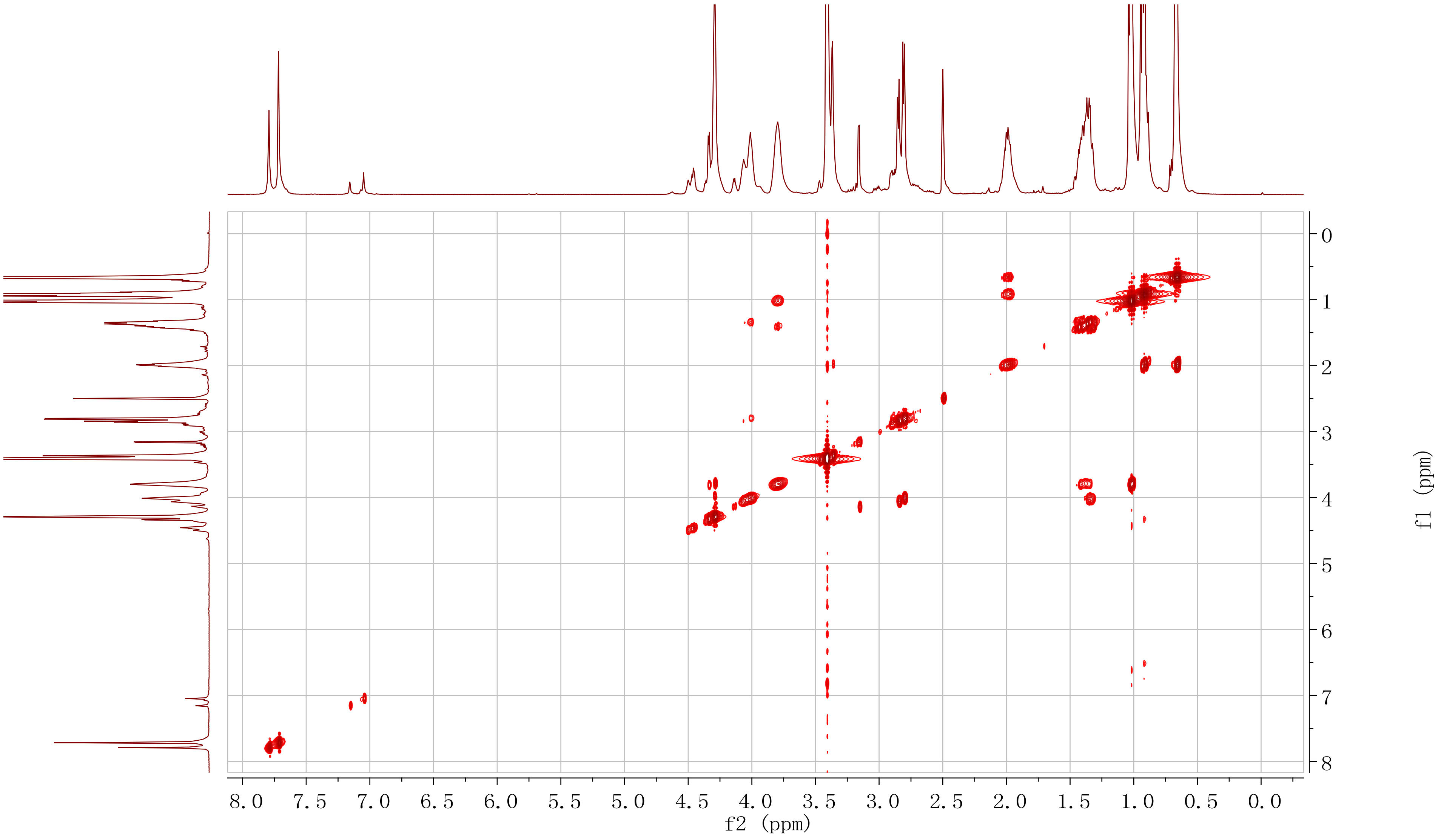

Figure S25. The IR spectrum of cladosporiumin L (**4**)

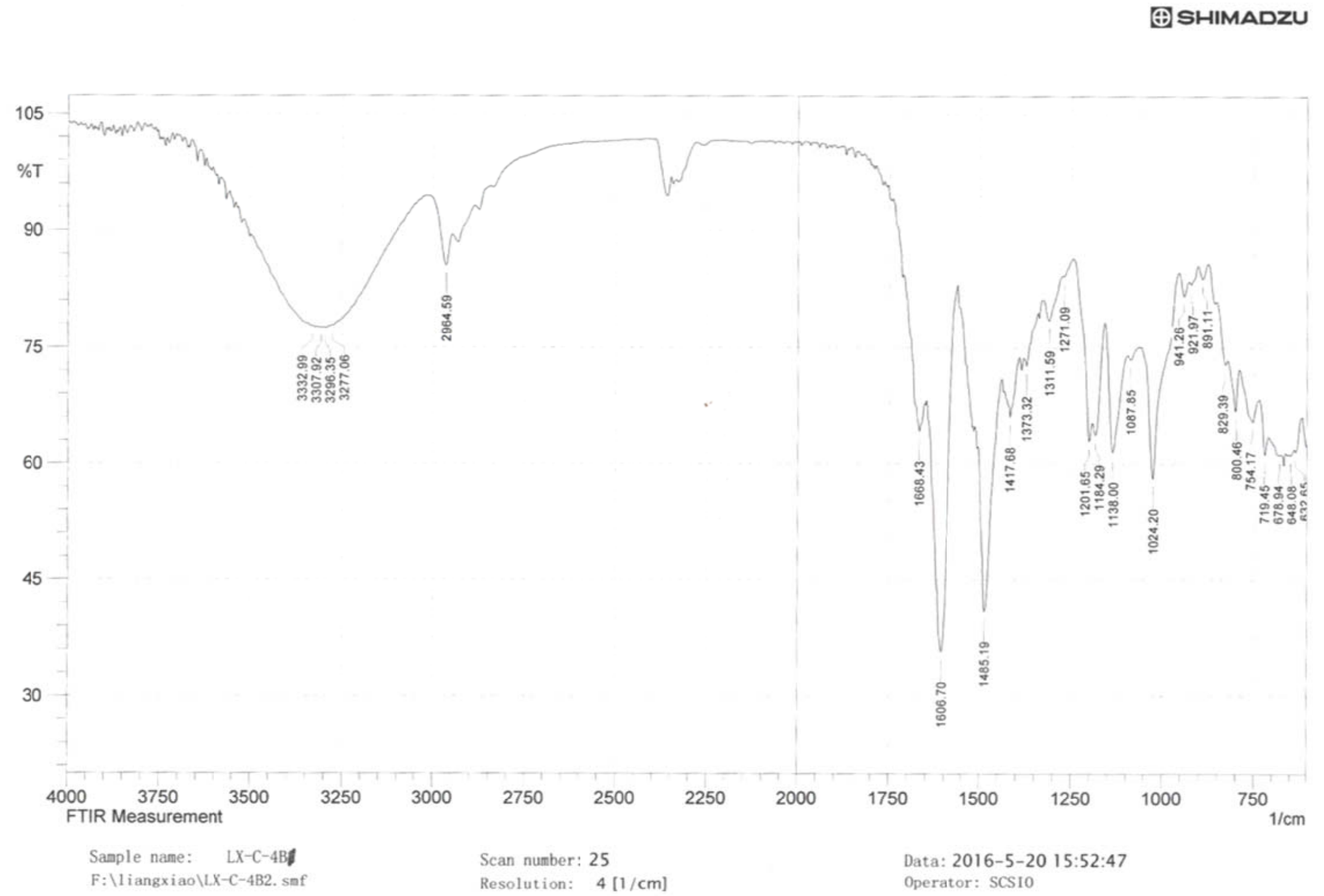

Figure S26. The (+)-HRESIMS spectrum of cladosporiumin L (**4**)

Mass Spectrum SmartFormula Report

Analysis Info

Analysis Name	D:\Data\MS\data\201604\liangxiao_LX-C-4B_1_01_1563.d	Acquisition Date	4/1/2016 6:20:56 PM
Method	LC_Direct Infusion_pos_100-1000mz.m	Operator	SCSIO
Sample Name	liangxiao_LX-C-4B	Instrument / Ser#	maXis 29
Comment			

Acquisition Parameter

Source Type	ESI	Ion Polarity	Positive	Set Nebulizer	0.4 Bar
Focus	Active	Set Capillary	4500 V	Set Dry Heater	180 °C
Scan Begin	100 m/z	Set End Plate Offset	-500 V	Set Dry Gas	4.0 l/min
Scan End	2000 m/z	Set Collision Cell RF	800.0 Vpp	Set Divert Valve	Waste

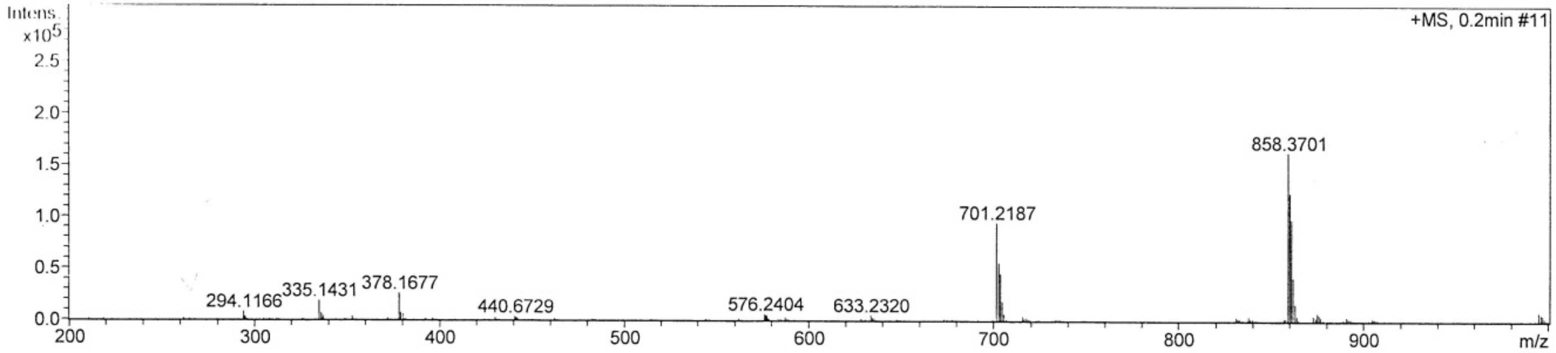

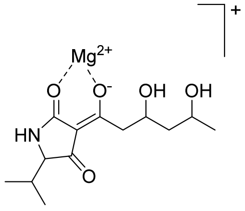

m/z 294.1166

$[M+Mg-H]^+$

Meas. m/z	#	Formula	Score	m/z	err [ppm]	err [mDa]	mSigma	rdb	e⁻ Conf	N-Rule
294.1166	1	C 13 H 20 Mg N O 5	62.93	294.1186	6.8	2.0	92.8	4.5	even	ok
	2	C 9 H 16 Mg N 7 O 3	100.00	294.1160	-2.4	-0.7	100.6	5.5	even	ok
	3	C 8 H 20 Mg N 3 O 7	23.42	294.1146	-6.9	-2.0	112.6	0.5	even	ok
	4	C 11 H 16 Mg 2 N 7	60.37	294.1163	-1.3	-0.4	113.6	7.5	even	ok
	5	C 10 H 20 Mg 2 N 3 O 4	25.85	294.1149	-5.9	-1.7	114.9	2.5	even	ok
	6	C 15 H 20 Mg 2 N O 2	13.46	294.1189	7.8	2.3	119.1	6.5	even	ok
858.3701	1	C 40 H 56 Mg 2 N 7 O 11	20.82	858.3733	3.7	3.2	30.9	16.5	even	ok
	2	C 38 H 44 Mg 2 N 21 O	20.62	858.3733	3.7	3.2	31.8	27.5	even	ok
	3	C 37 H 48 Mg 2 N 17 O 5	56.62	858.3720	2.2	1.9	37.8	22.5	even	ok
	4	C 39 H 60 Mg 2 N 3 O 15	53.34	858.3720	2.2	1.9	39.7	11.5	even	ok
	5	C 36 H 52 Mg 2 N 13 O 9	100.00	858.3706	0.6	0.5	46.5	17.5	even	ok
	6	C 33 H 44 Mg 2 N 23 O 3	74.26	858.3693	-1.0	-0.8	51.4	23.5	even	ok
	7	C 35 H 56 Mg 2 N 9 O 13	69.95	858.3693	-0.9	-0.8	53.4	12.5	even	ok
	8	C 32 H 48 Mg 2 N 19 O 7	23.06	858.3679	-2.5	-2.2	60.1	18.5	even	ok
	9	C 34 H 60 Mg 2 N 5 O 17	21.30	858.3680	-2.5	-2.1	62.6	7.5	even	ok
	10	C 31 H 52 Mg 2 N 15 O 11	5.16	858.3666	-4.1	-3.5	67.2	13.5	even	ok

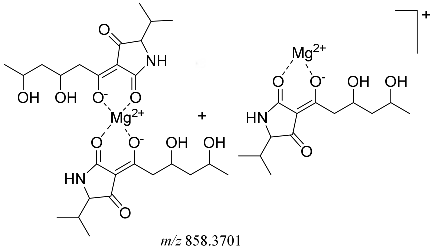

m/z 858.3701

$[3M+2Mg-3H]^+$

Figure S27. The ^{1}H-NMR spectrum of cladosporiumin M (**5**) in $CDCl_3$

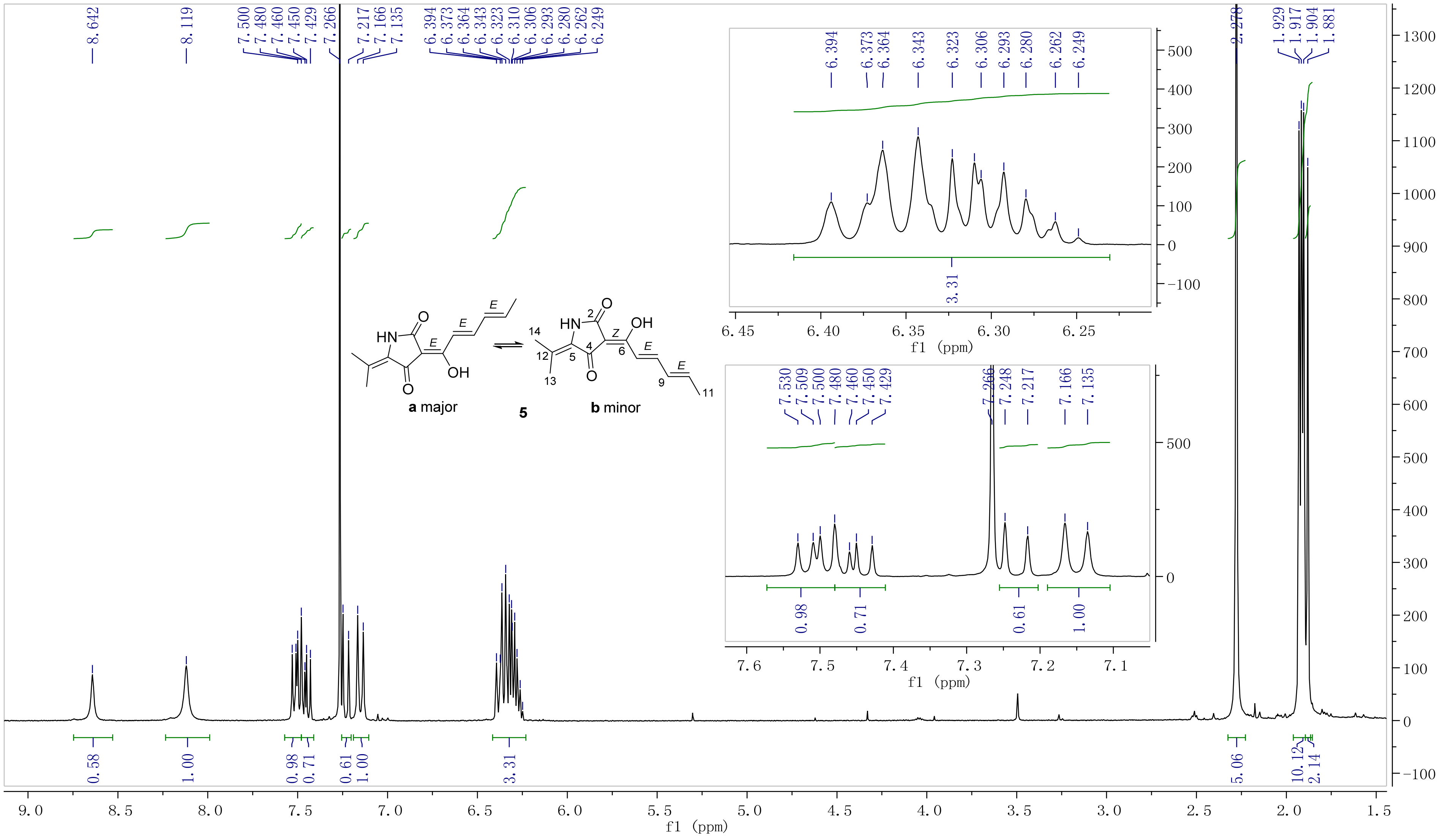

Figure S28. The ^{13}C NMR spectrum of cladosporiumin M (**5**) in $CDCl_3$

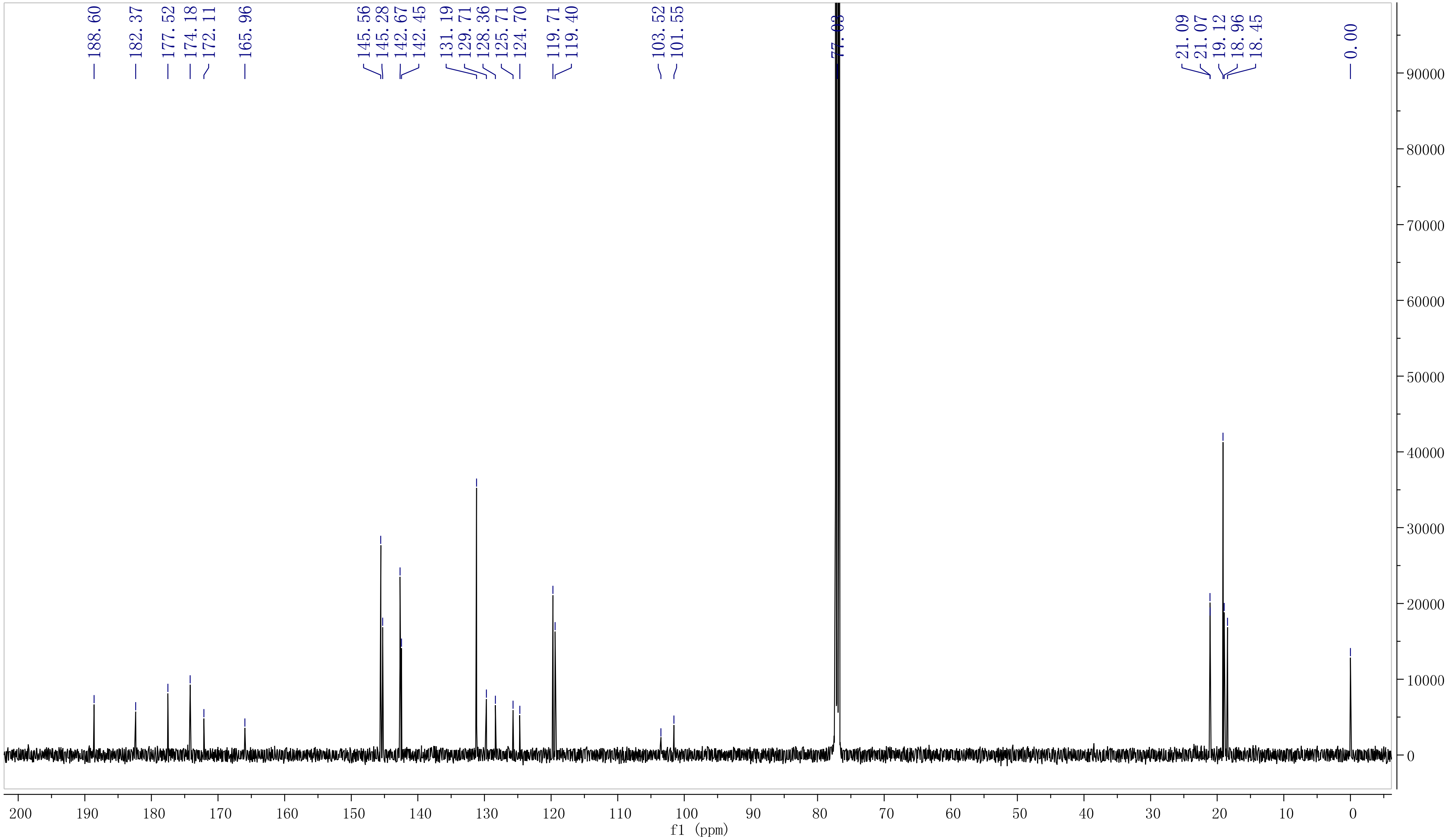

Figure S29. The HSQC spectrum of cladosporiumin M (**5**) in $CDCl_3$

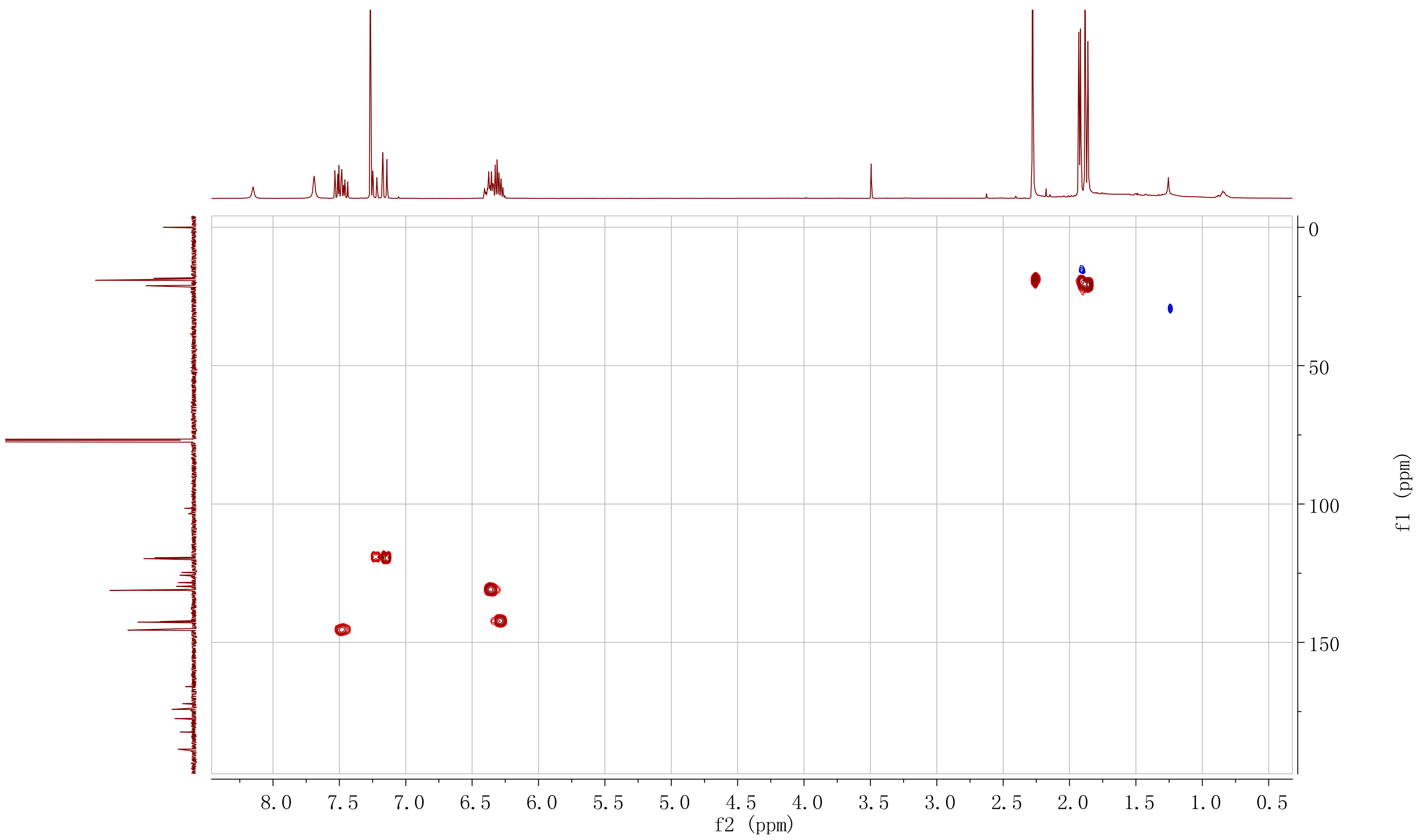

Figure S30. The HMBC spectrum of cladosporiumin M (**5**) in $CDCl_3$

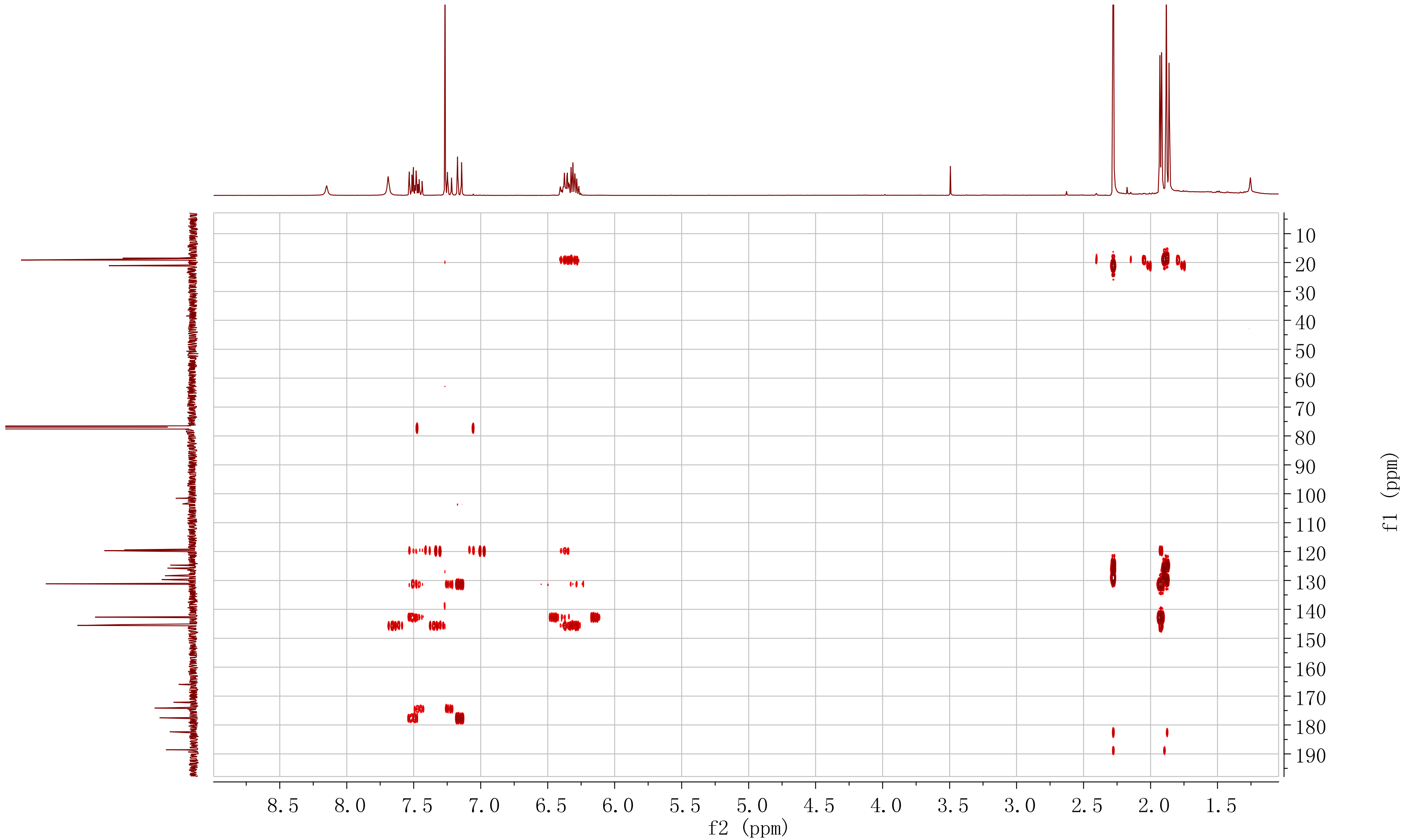

Figure S31. The ^{1}H-^{1}H COSY spectrum of cladosporiumin M (**5**) in $CDCl_3$

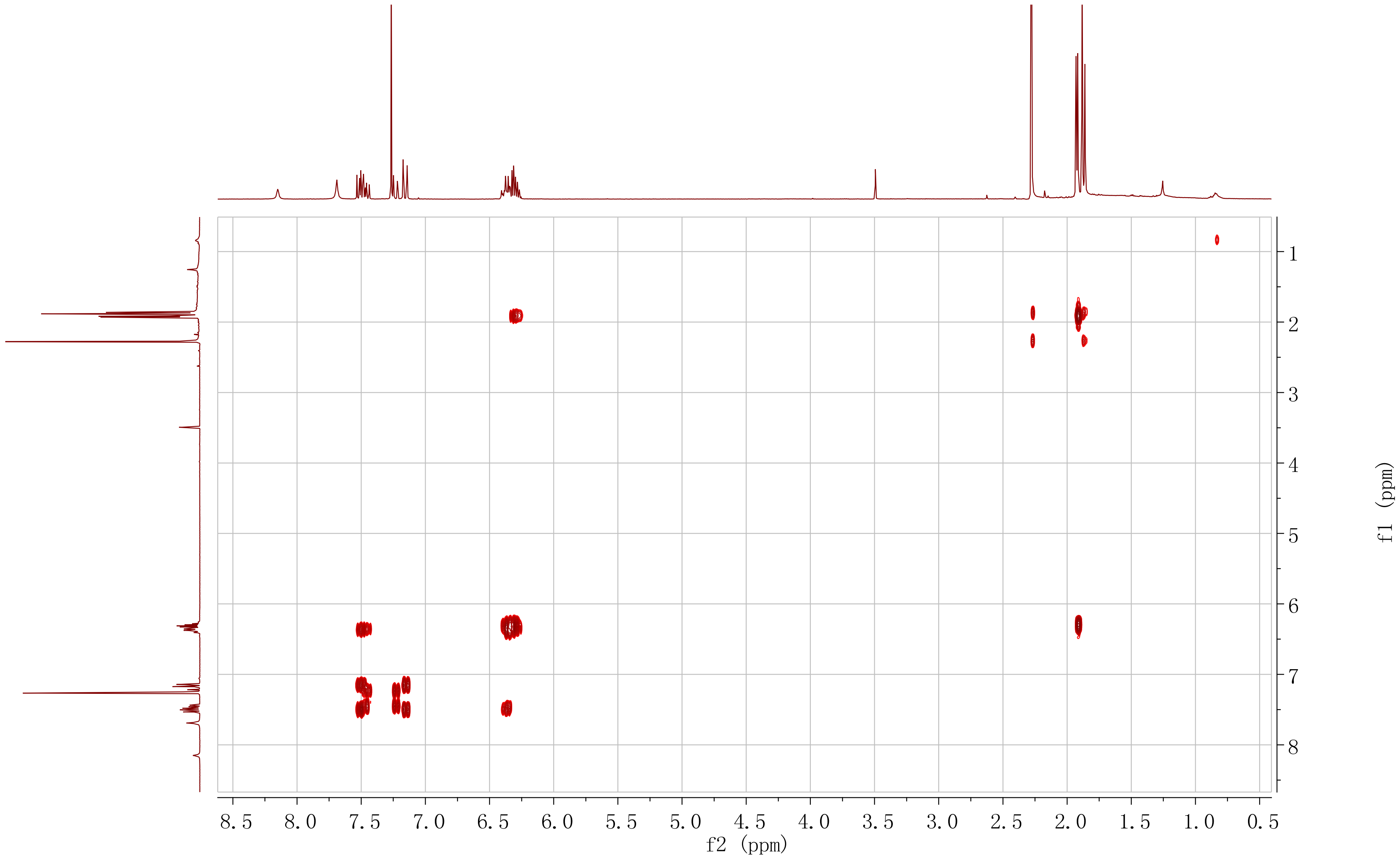

Figure S32. The IR spectrum of cladosporiumin M (**5**)

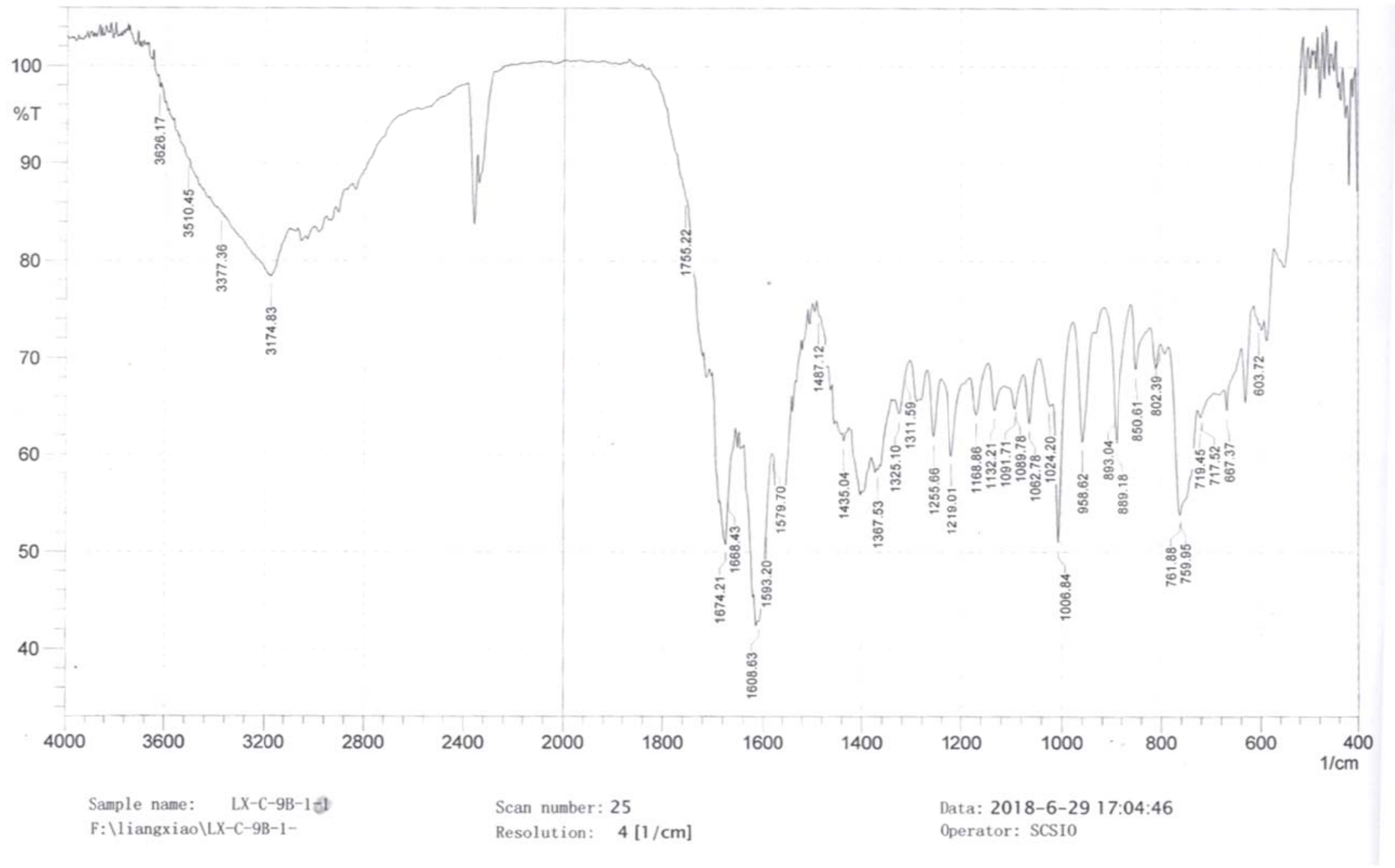

Figure S33. The (+)-HRESIMS spectrum of cladosporiumin M (**5**)

Mass Spectrum SmartFormula Report

Analysis Info

Analysis Name	D:\Data\MS\data\201806\liangxiao_LX-C-9B-1_pos_92_01_4922.d	Acquisition Date	6/9/2018 4:13:30 PM
Method	LC_Direct Infusion_pos_70-500mz.m	Operator	SCSIO
Sample Name	liangxiao_LX-C-9B-1_pos	Instrument	maXis 255552.00029
Comment			

Acquisition Parameter

Source Type	ESI	Ion Polarity	Positive	Set Nebulizer	0.4 Bar
Focus	Active	Set Capillary	4500 V	Set Dry Heater	180 °C
Scan Begin	70 m/z	Set End Plate Offset	-500 V	Set Dry Gas	4.0 l/min
Scan End	1500 m/z	Set Charging Voltage	0 V	Set Divert Valve	Waste
		Set Corona	0 nA	Set APCI Heater	0 °C

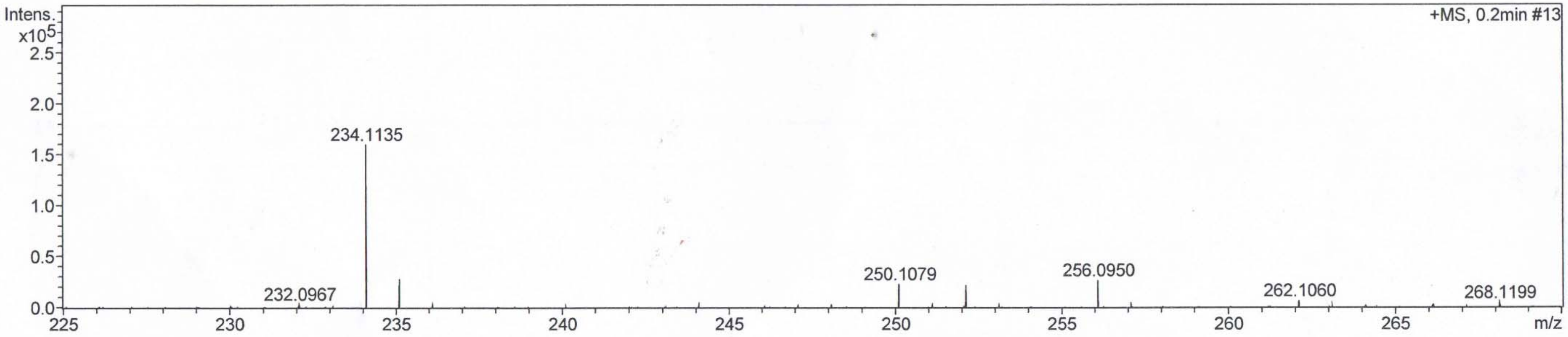

Meas. m/z	#	Ion Formula	Score	m/z	err [ppm]	err [mDa]	mSigma	rdb	e⁻ Conf	N-Rule
234.1135	1	C13H16NO3	100.00	234.1125	4.2	1.0	16.5	6.5	even	ok
256.0950	1	C13H15NNaO3	100.00	256.0944	-2.5	-0.6	23.8	6.5	even	ok
467.2180	1	C26H31N2O6	100.00	467.2177	-0.7	-0.3	31.1	12.5	even	ok
489.1995	1	C26H30N2NaO6	100.00	489.1996	0.3	0.1	26.0	12.5	even	ok

Figure S34. The ^{1}H-NMR spectrum of cladosporiumin N (**6**) in DMSO-d_6

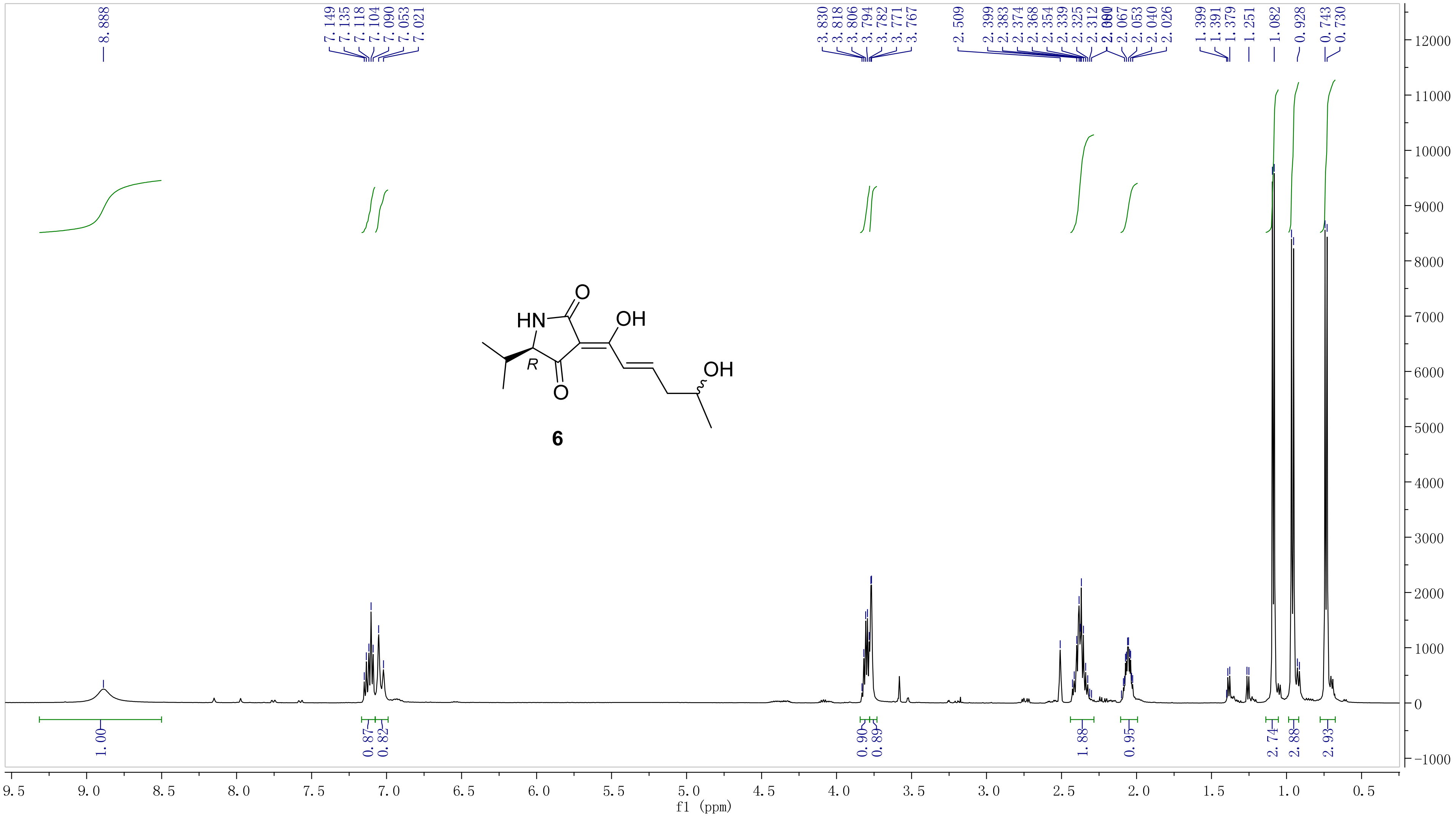

Figure S35. The ^{13}C NMR spectrum of cladosporiumin N (**6**) in DMSO-d_6

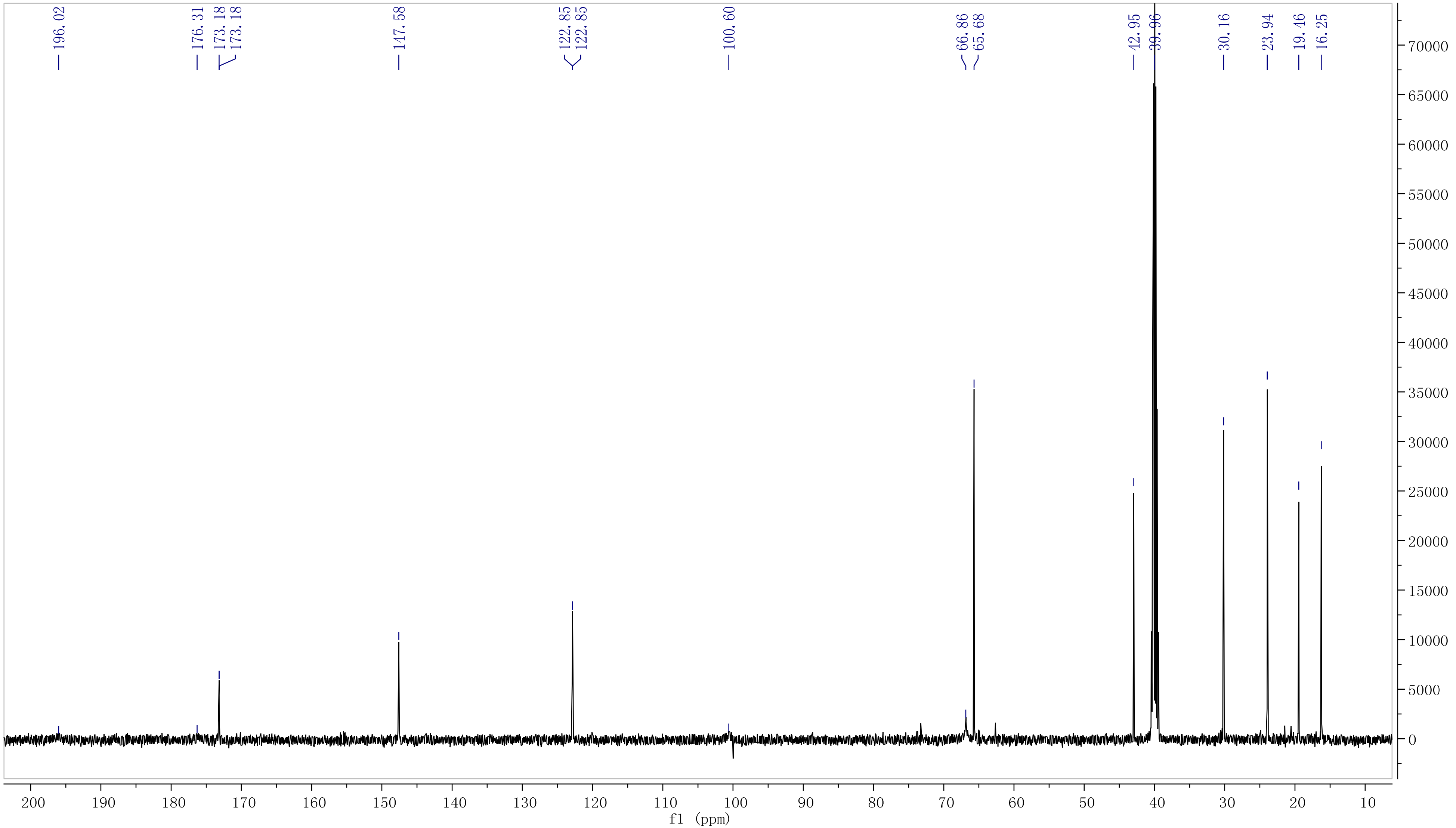

Figure S36. The HSQC spectrum of cladosporiumin N (**6**) in DMSO-d_6

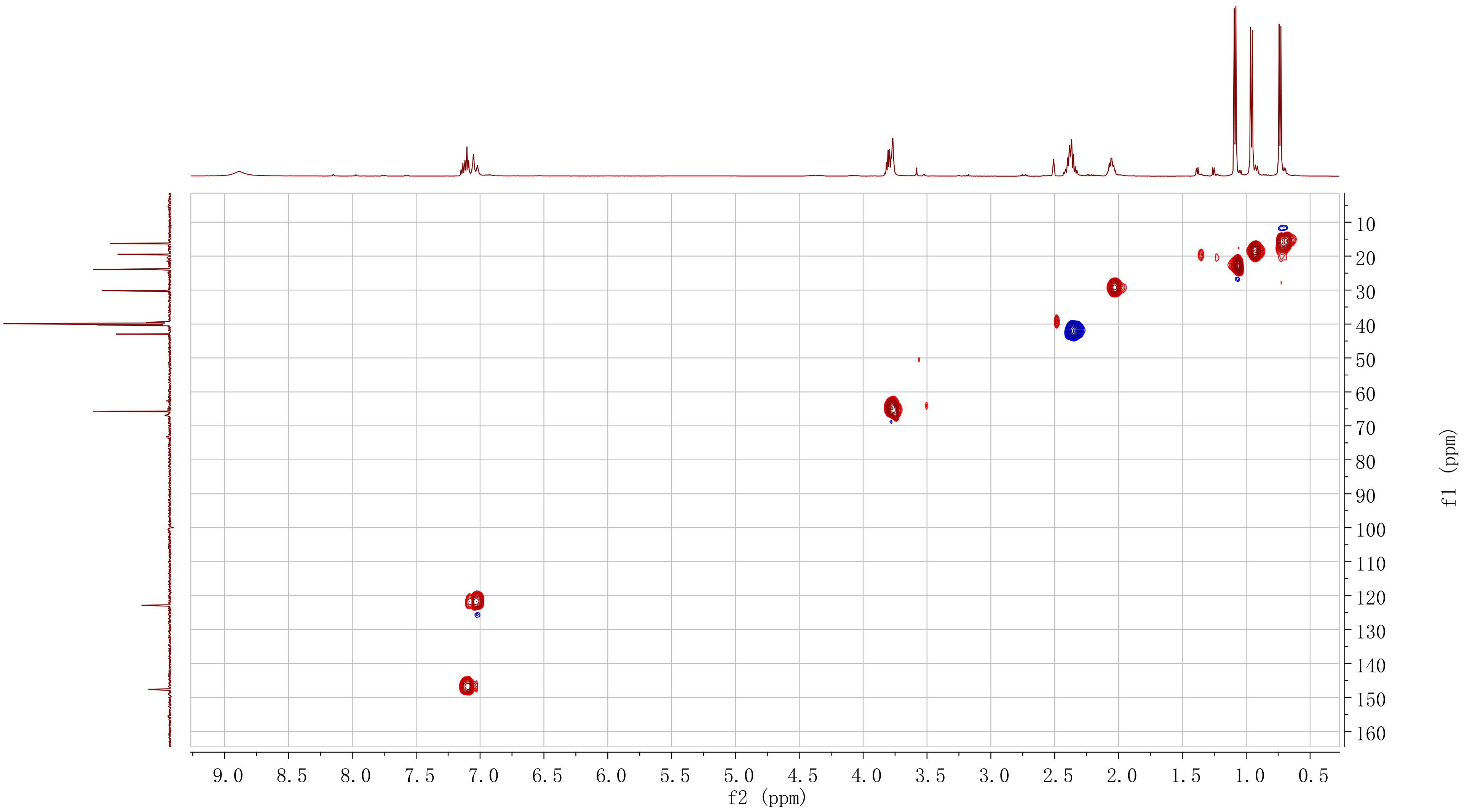

Figure S37. The HMBC spectrum of cladosporiumin N (**6**) in DMSO-d_6

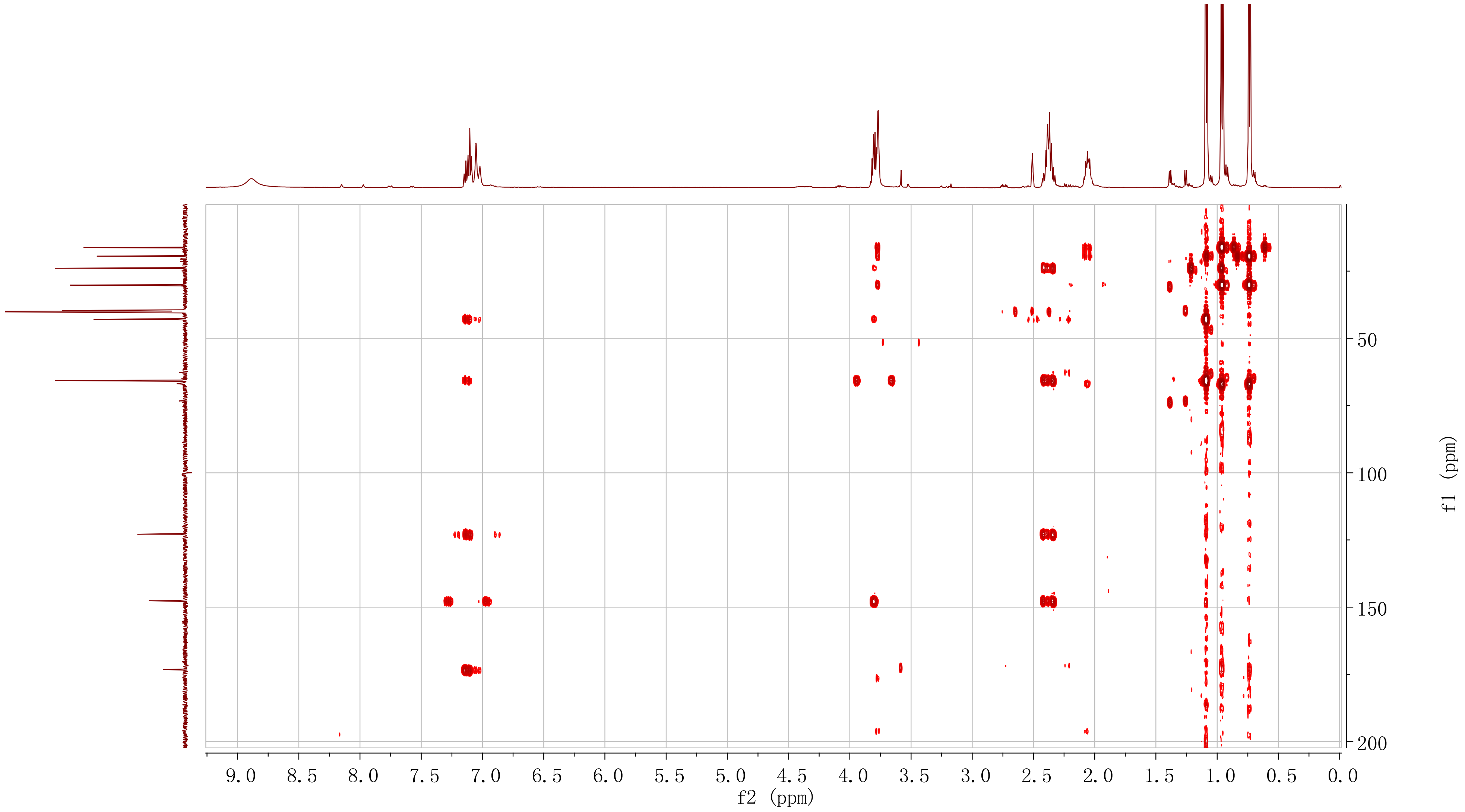

Figure S38. The ^{1}H-^{1}H COSY spectrum of cladosporiumin N (**6**) in DMSO-d_6

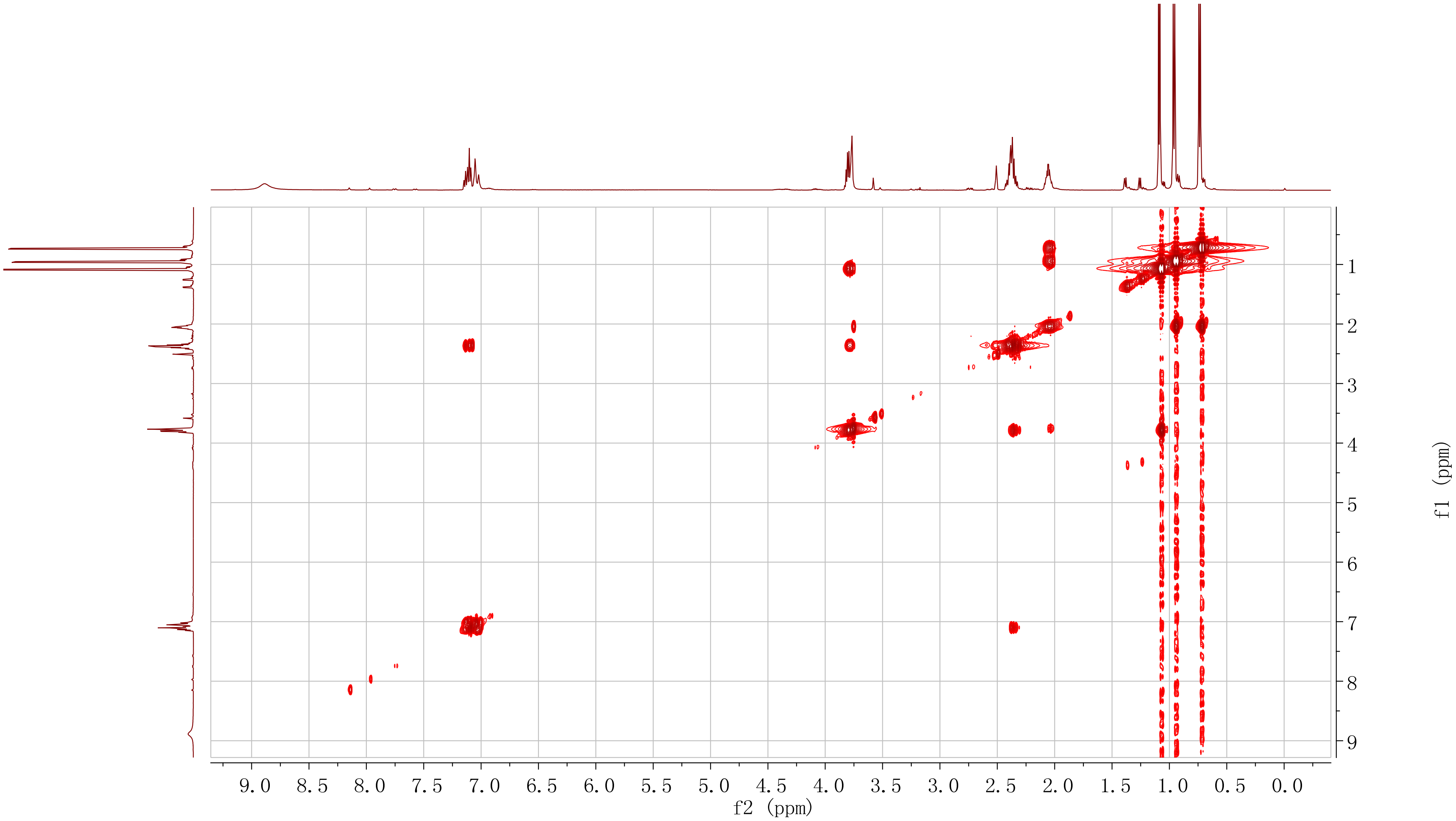

Figure S39. The (+)-HRESIMS spectrum of cladosporiumin N (**6**)

Mass Spectrum SmartFormula Report

Analysis Info

Analysis Name	D:\Data\MS\data\201603\liangxiao_LX-C-5_41_01_1507.d	Acquisition Date	3/22/2016 11:40:18 AM
Method	LC_Direct Infusion_pos_100-1000mz.m	Operator	SCSIO
Sample Name	liangxiao_LX-C-5	Instrument / Ser#	maXis 29
Comment			

Acquisition Parameter

Source Type	ESI	Ion Polarity	Positive	Set Nebulizer	0.4 Bar
Focus	Active	Set Capillary	4500 V	Set Dry Heater	180 °C
Scan Begin	100 m/z	Set End Plate Offset	-500 V	Set Dry Gas	4.0 l/min
Scan End	2000 m/z	Set Collision Cell RF	800.0 Vpp	Set Divert Valve	Waste

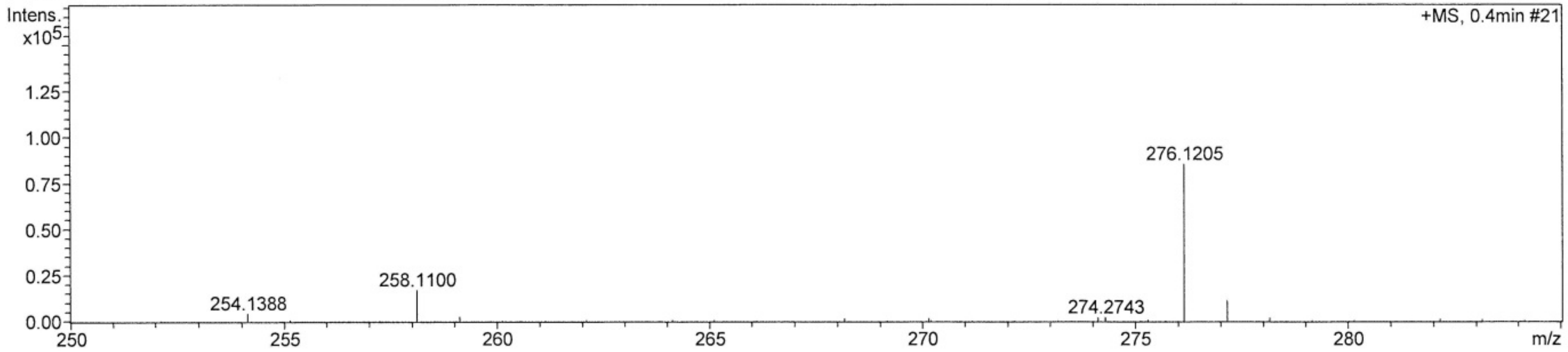

Meas. m/z	#	Formula	Score	m/z	err [ppm]	err [mDa]	mSigma	rdb	e⁻ Conf	N-Rule
254.1388	1	C 13 H 20 N O 4	100.00	254.1387	-0.6	-0.2	6.3	4.5	even	ok
	2	C 9 H 16 N 7 O 2	13.28	254.1360	-11.2	-2.8	17.7	5.5	even	ok
	3	C 8 H 20 N 3 O 6	2.33	254.1347	-16.5	-4.2	30.7	0.5	even	ok
276.1205	1	C 13 H 19 N Na O 4	100.00	276.1206	0.6	0.2	6.7	4.5	even	ok
	2	C 9 H 15 N 7 Na O 2	21.62	276.1179	-9.1	-2.5	7.8	5.5	even	ok
	3	C 8 H 19 N 3 Na O 6	4.32	276.1166	-14.0	-3.9	20.8	0.5	even	ok
529.2512	1	C 26 H 38 N 2 Na O 8	100.00	529.2520	1.5	0.8	46.6	8.5	even	ok
	2	C 20 H 42 Na O 14	2.95	529.2467	-8.6	-4.6	50.4	-0.5	even	ok
	3	C 22 H 34 N 8 Na O 6	42.81	529.2494	-3.6	-1.9	52.0	9.5	even	ok
	4	C 18 H 30 N 14 Na O 4	2.17	529.2467	-8.6	-4.6	59.5	10.5	even	ok
	5	C 32 H 34 N 4 Na O 2	0.18	529.2574	11.6	6.2	62.9	17.5	even	ok

Figure S40. The ^{1}H-NMR spectrum of cladosporiumin O (**7**) in DMSO-d_6

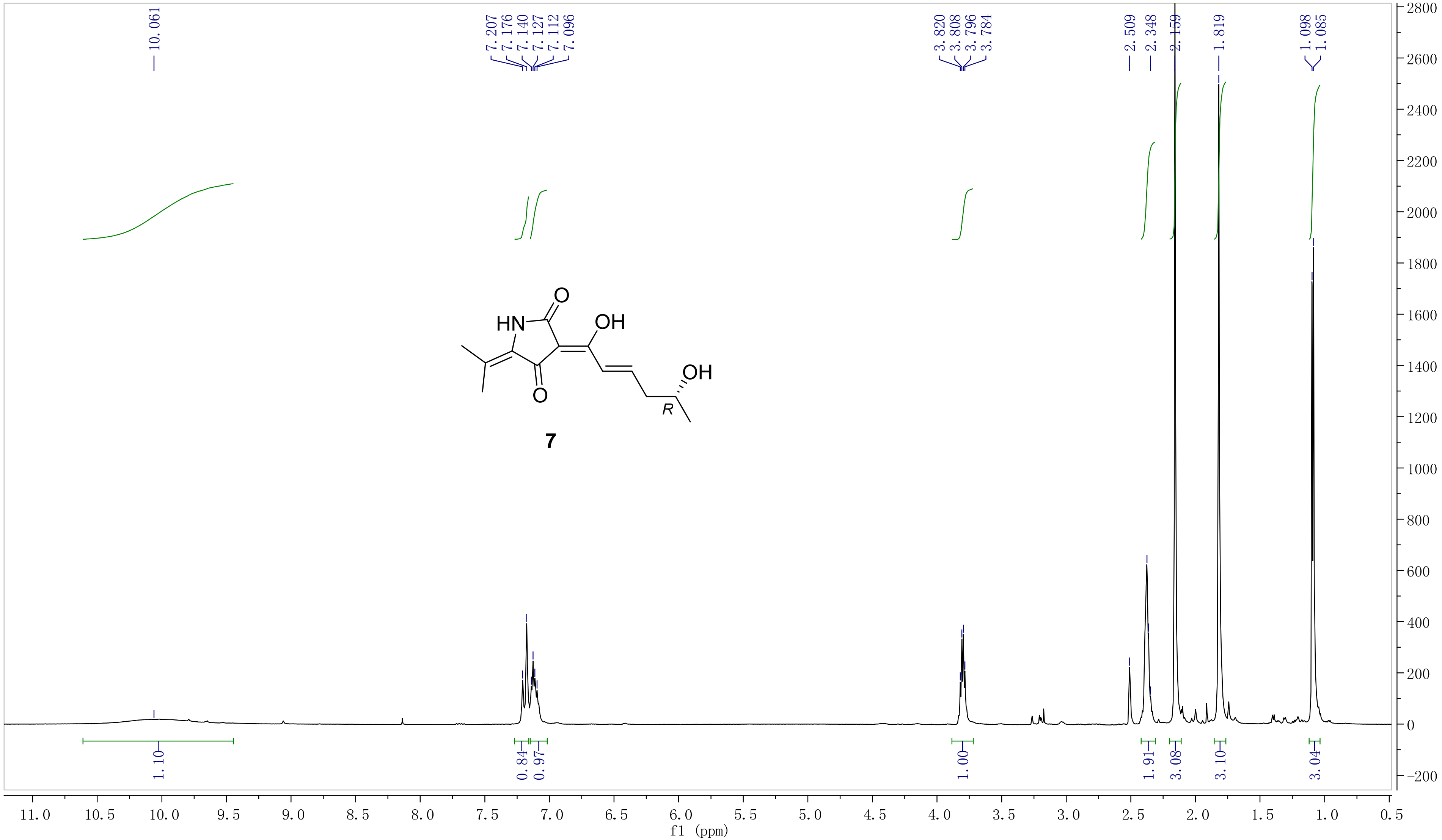

Figure S41. The ^{13}C NMR spectrum of cladosporiumin O (**7**) in DMSO-d_6

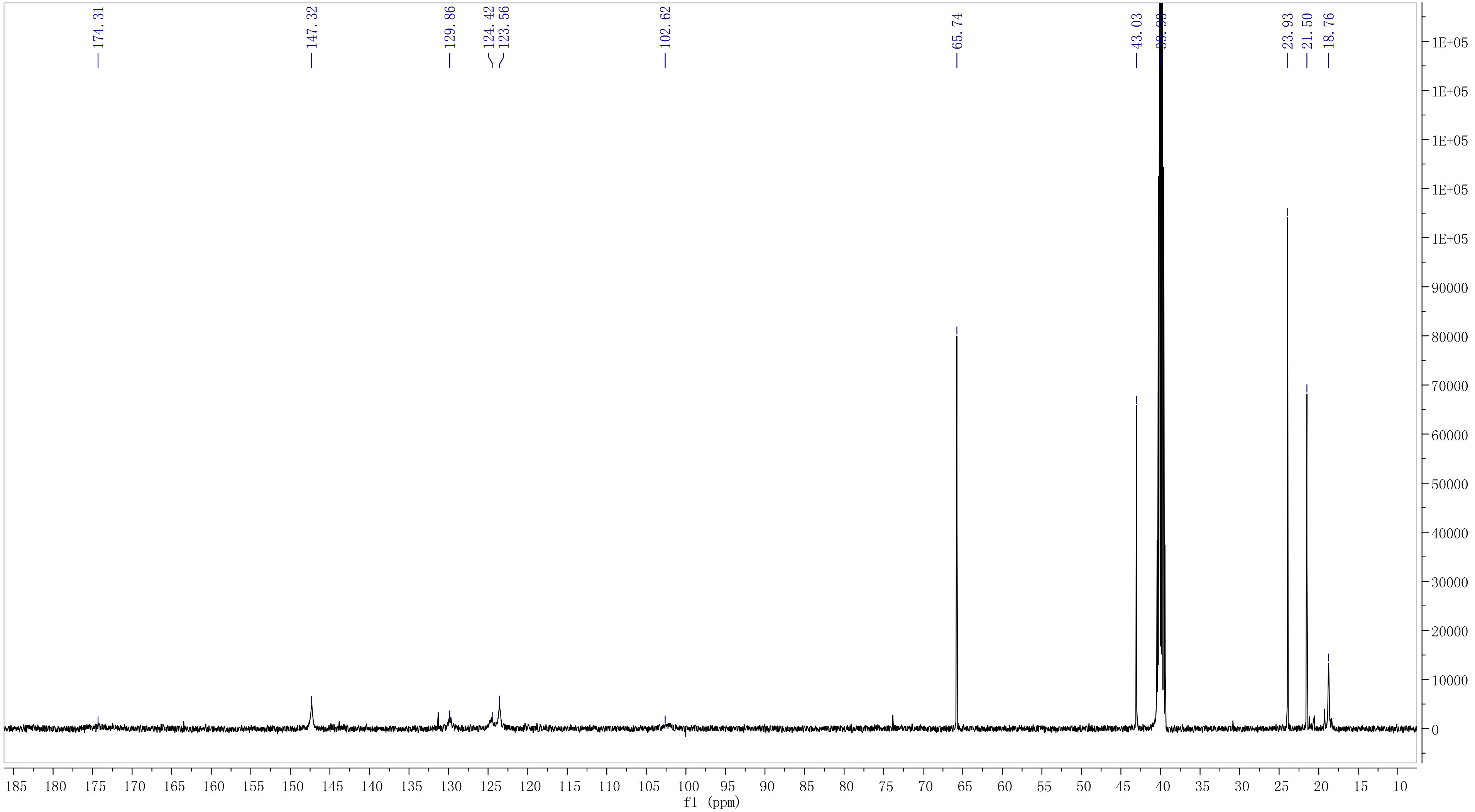

Figure S42. The HMBC spectrum of cladosporiumin O (**7**) in DMSO-d_6

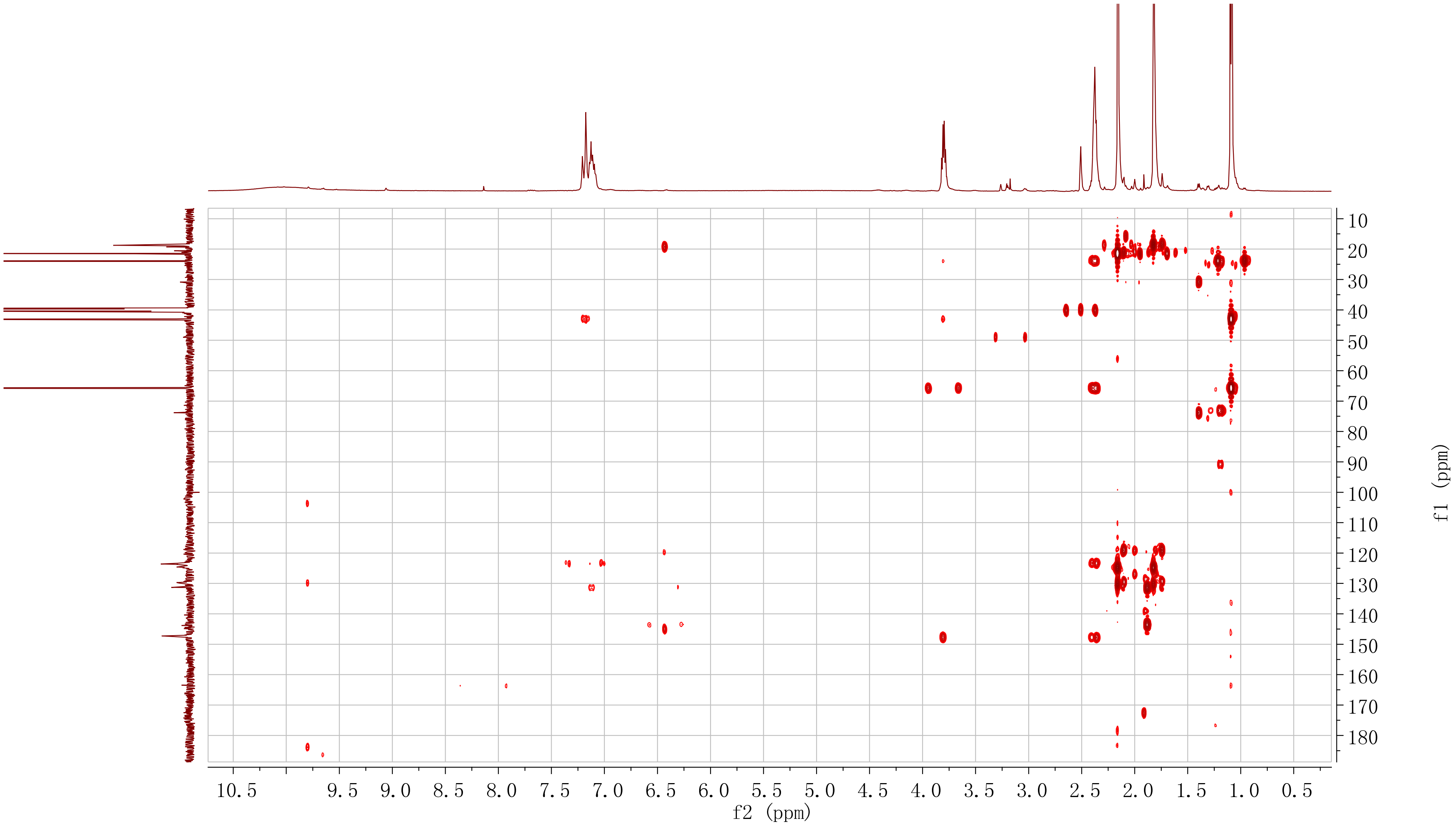

Figure S43. The (+)-HRESIMS spectrum of cladosporiumin O (**7**)

Mass Spectrum SmartFormula Report

Analysis Info

Analysis Name	D:\Data\MS\data\201604\liangxiao_LX-C-9B_pos_23_01_1834.d	Acquisition Date	5/17/2016 5:39:56 PM
Method	LC_Direct Infusion_pos_100-1000mz.m	Operator	SCSIO
Sample Name	liangxiao_LX-C-9B_pos	Instrument / Ser#	maXis 29
Comment			

Acquisition Parameter

Source Type	ESI	Ion Polarity	Positive	Set Nebulizer	0.4 Bar
Focus	Active	Set Capillary	4500 V	Set Dry Heater	180 °C
Scan Begin	100 m/z	Set End Plate Offset	-500 V	Set Dry Gas	4.0 l/min
Scan End	2000 m/z	Set Collision Cell RF	800.0 Vpp	Set Divert Valve	Waste

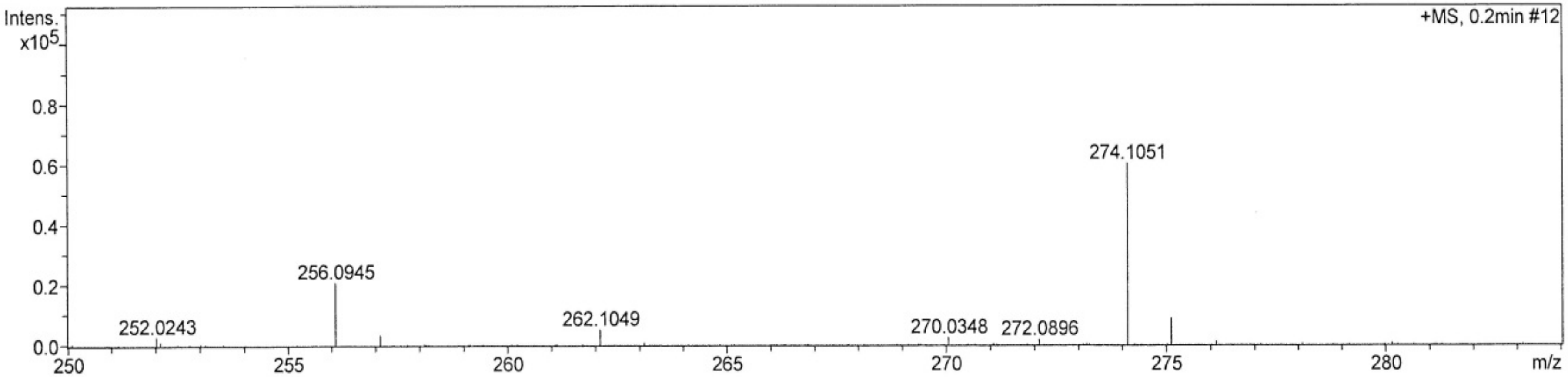

Meas. m/z	#	Formula	Score	m/z	err [ppm]	err [mDa]	mSigma	rdb	e⁻ Conf	N-Rule
252.1233	1	C 13 H 18 N O 4	100.00	252.1230	-0.9	-0.2	39.9	5.5	even	ok
274.1051	1	C 13 H 17 N Na O 4	100.00	274.1050	-0.4	-0.1	1.2	5.5	even	ok
525.2202	1	C 26 H 34 N 2 Na O 8	90.09	525.2207	0.9	0.5	20.5	10.5	even	ok

Figure S44. The ^{1}H-NMR spectrum of cladodionen (**8**) in DMSO-d_6

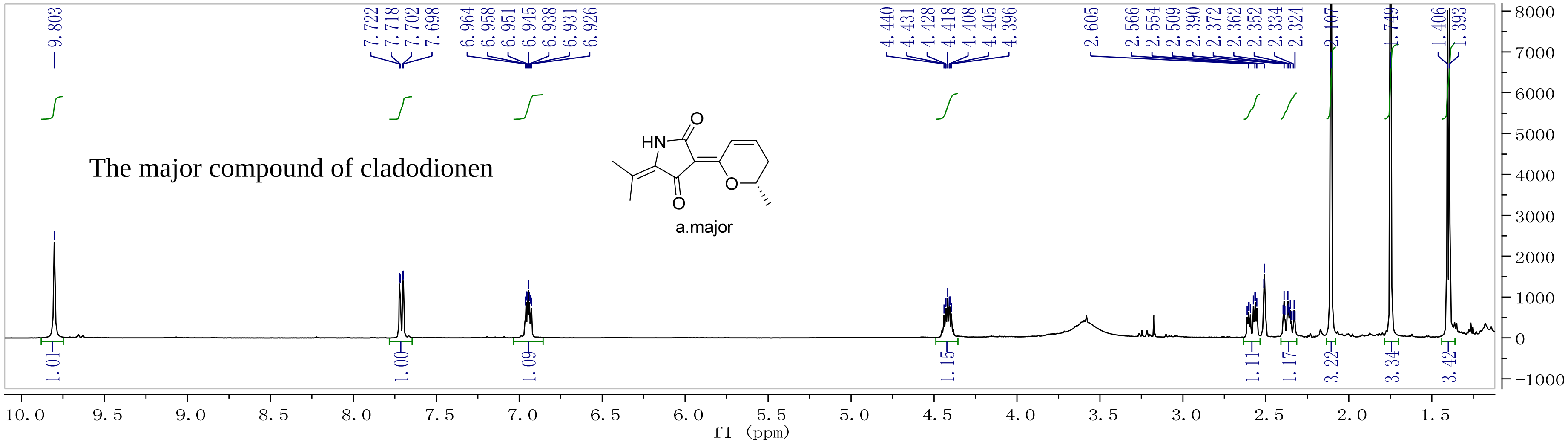

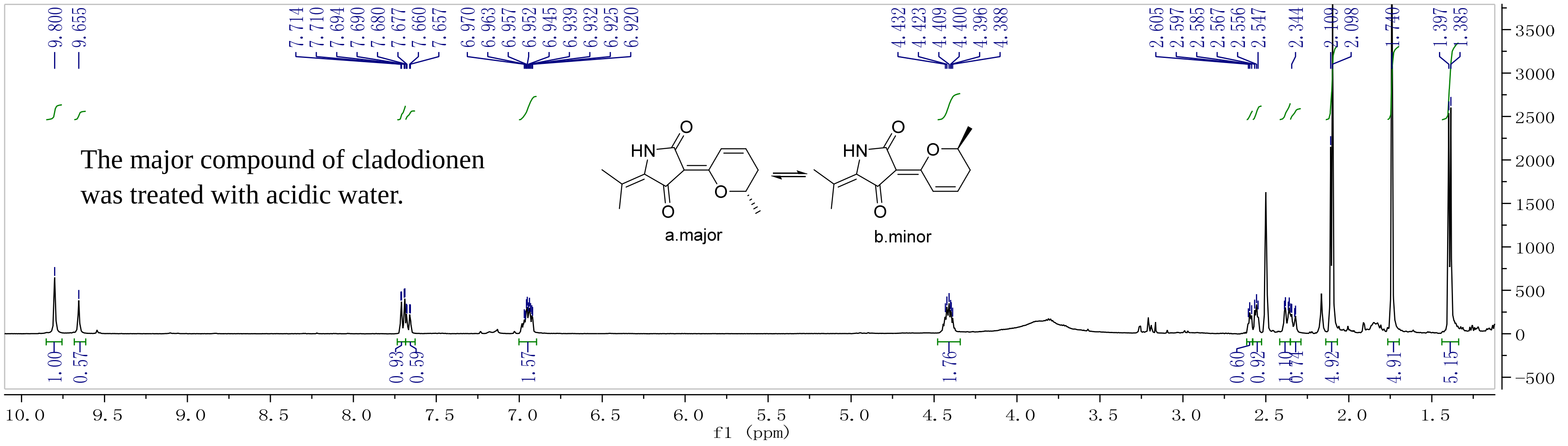

Figure S45. The ^{13}C NMR spectrum of cladodionen (**8**) in DMSO-d_6

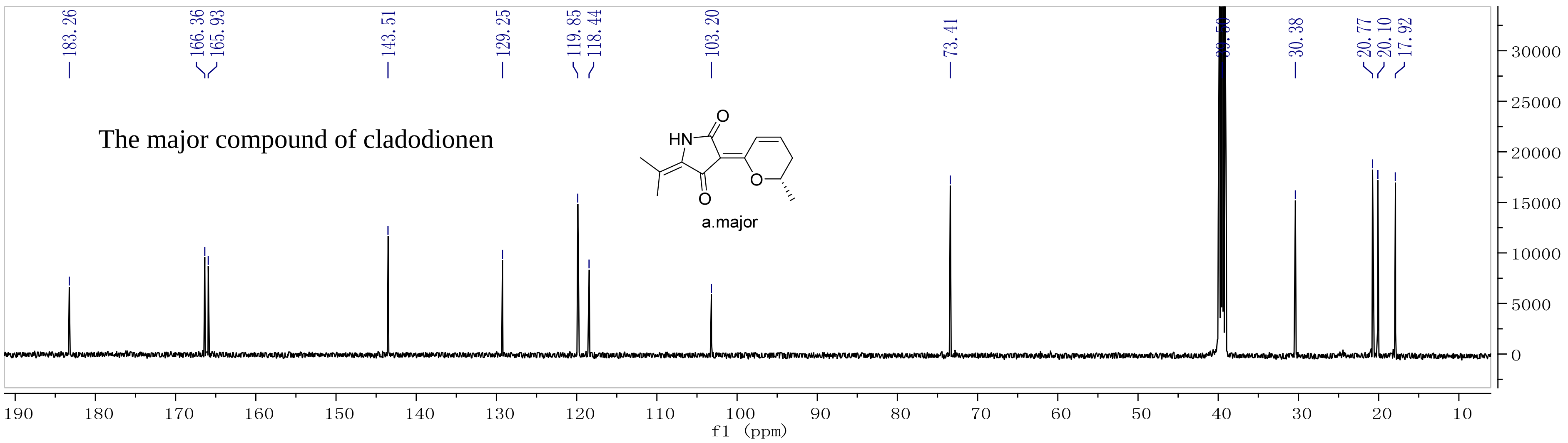

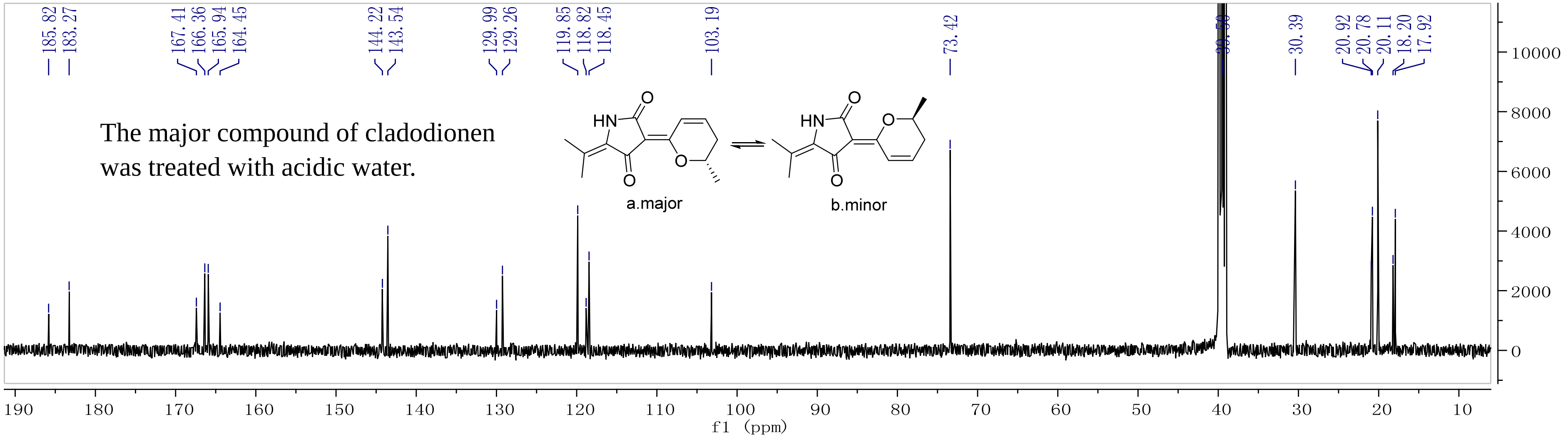

Figure S46. The IR spectrum of cladodionen (**8**)

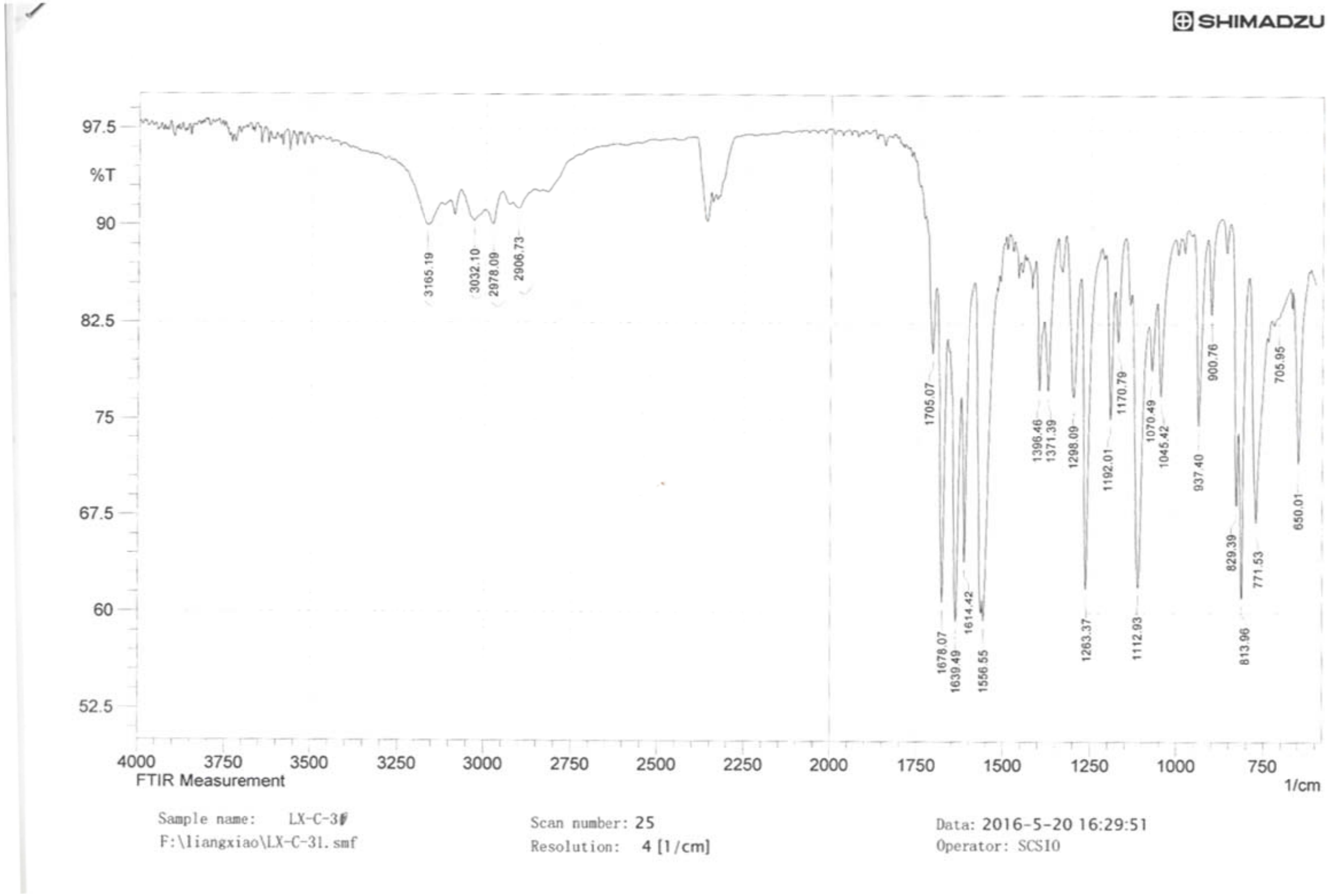

Figure S47. The (+)-HRESIMS spectrum of cladodionen (**8**)

Mass Spectrum SmartFormula Report

Analysis Info

Analysis Name	D:\Data\MS\data\201604\liangxiao_LX-C-2_pos_22_01_1835.d	Acquisition Date	5/17/2016 5:43:18 PM
Method	LC_Direct Infusion_pos_100-1000mz.m	Operator	SCSIO
Sample Name	liangxiao_LX-C-2_pos	Instrument / Ser#	maXis 29
Comment			

Acquisition Parameter

Source Type	ESI	Ion Polarity	Positive	Set Nebulizer	0.4 Bar
Focus	Active	Set Capillary	4500 V	Set Dry Heater	180 °C
Scan Begin	100 m/z	Set End Plate Offset	-500 V	Set Dry Gas	4.0 l/min
Scan End	2000 m/z	Set Collision Cell RF	800.0 Vpp	Set Divert Valve	Waste

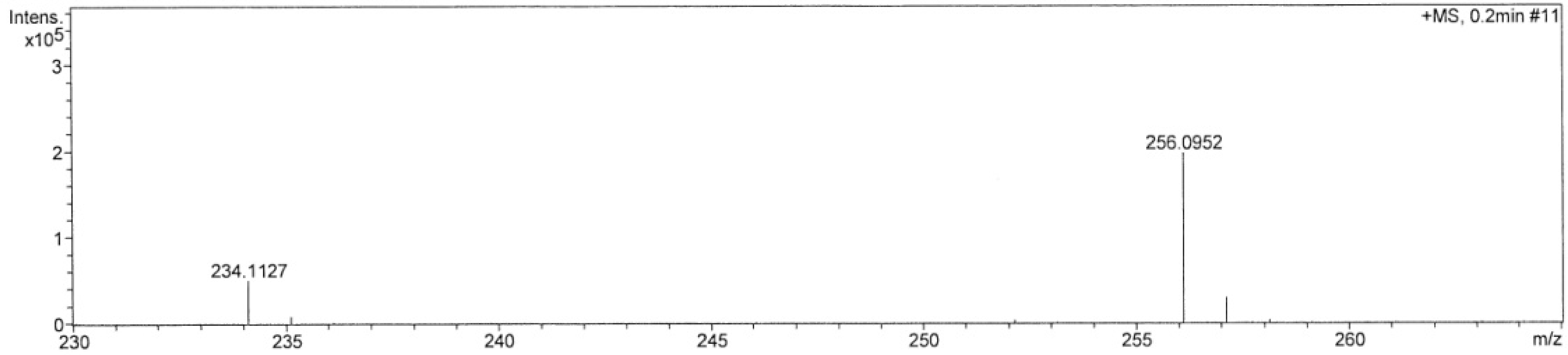

Meas. m/z	#	Formula	Score	m/z	err [ppm]	err [mDa]	mSigma	rdb	e¯ Conf	N-Rule
234.1127	1	C 13 H 16 N O 3	100.00	234.1125	-1.0	-0.2	9.1	6.5	even	ok
256.0952	1	C 13 H 15 N Na O 3	100.00	256.0944	-3.2	-0.8	1.7	6.5	even	ok
489.2006	1	C 26 H 30 N 2 Na O 6	42.98	489.1996	-2.1	-1.0	48.3	12.5	even	ok

Figure S48. HPLC analysis of FDAA derivates of acidic hydrolysates of cladosporiumin L (**4**) (Column: YMC-Pack ODS-A column, 250×4.6 mmI.D. , S-5 μm, 12 nm).

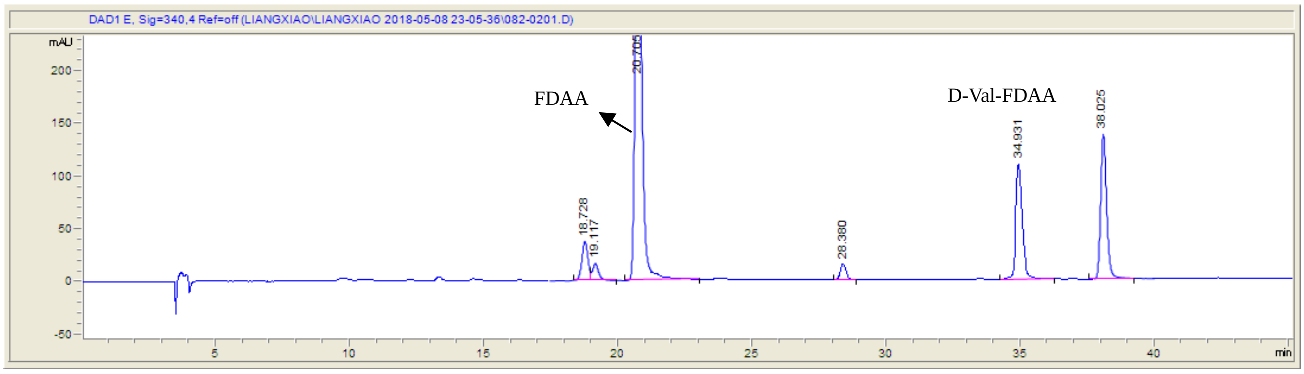

Figure S49. HPLC analysis of FDAA derivates of D-Val and L-Val (Column: YMC-Pack ODS-A column, 250×4.6 mmI.D. , S-5 μm, 12 nm).

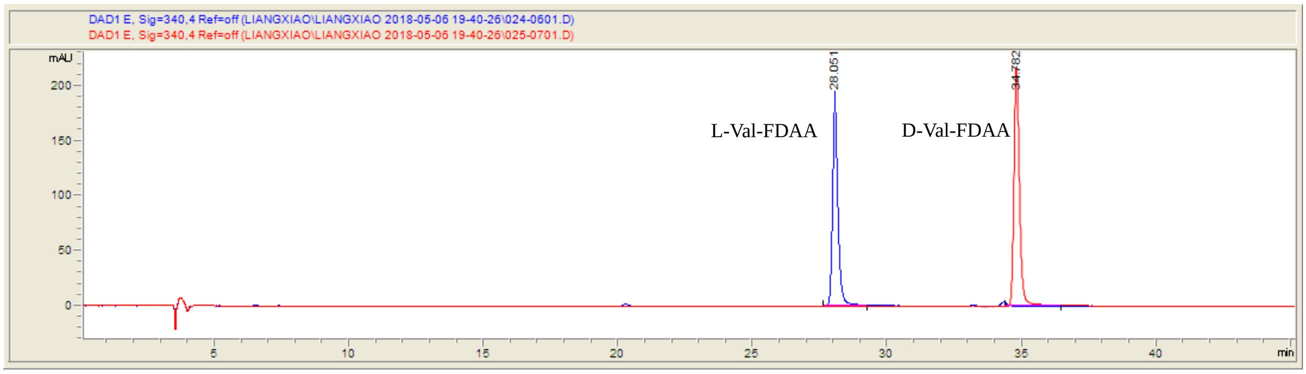

Figure S50. HPLC chromatograms of compound cladosporiumin L (**4**) before and after acid treatment

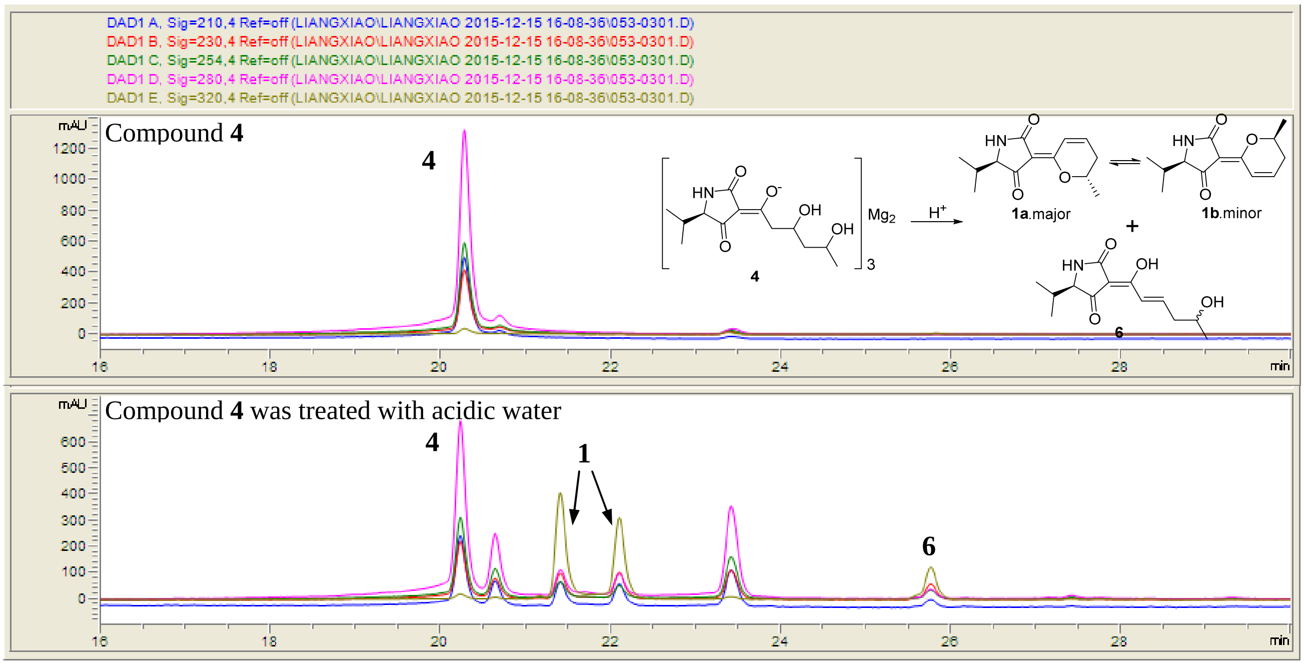

Figure S51. The ICP-AES analysis report of magnesium content in compound cladosporiumin L (**4**)

中 山 大 学 测 试 中 心

分 析 报 告

来 样 单 位：中国科学院南海海洋研究所　　来样日期：2018-05-03

样品原标识：LX-C-4B、LX-C-4B（空白）　　样品外观：无色液体

分 析 项 目：Mg　　检测日期：2018-05-04

检测仪器： 全谱直读等离子体原子发射光谱仪，Optima 8300（美国 PerkinElmer）
检测条件： 入射功率：1300W　等离子气流量：15L/min　雾化气流量：0.6 L/min
检测依据： JY/T 015-1996 （国家教委"感耦等离子体原子发射光谱方法通则"）

检测结果(mg/L) → The concentration of Mg

编号/元素	Mg	
LX-C-4B（空白）	0.073	→ Blank control
LX-C-4B	6.79	→ Compound **4**

常规检测　以下空白

注：1、本分析报告仅对送检样品负责。如对报告内容有疑问，请在报告发出之日起 15 个工作日内（邮寄邮戳或邮寄日期为准）向本中心业务办公室提出书面申请，同时附上本报告原件及复印件，过期恕不受理。
2、常规检测数据和结果仅用于科研、教学、企业内部质量控制等，不具有对社会的证明作用。
3、本报告附图 0 页。分析编号：20180504A.2。

报告编号：1805B0022　　检测人：[signature]　　校核人：[signature]

Figure S52. The (+)-HRESIMS spectrum of cladosporiumin E

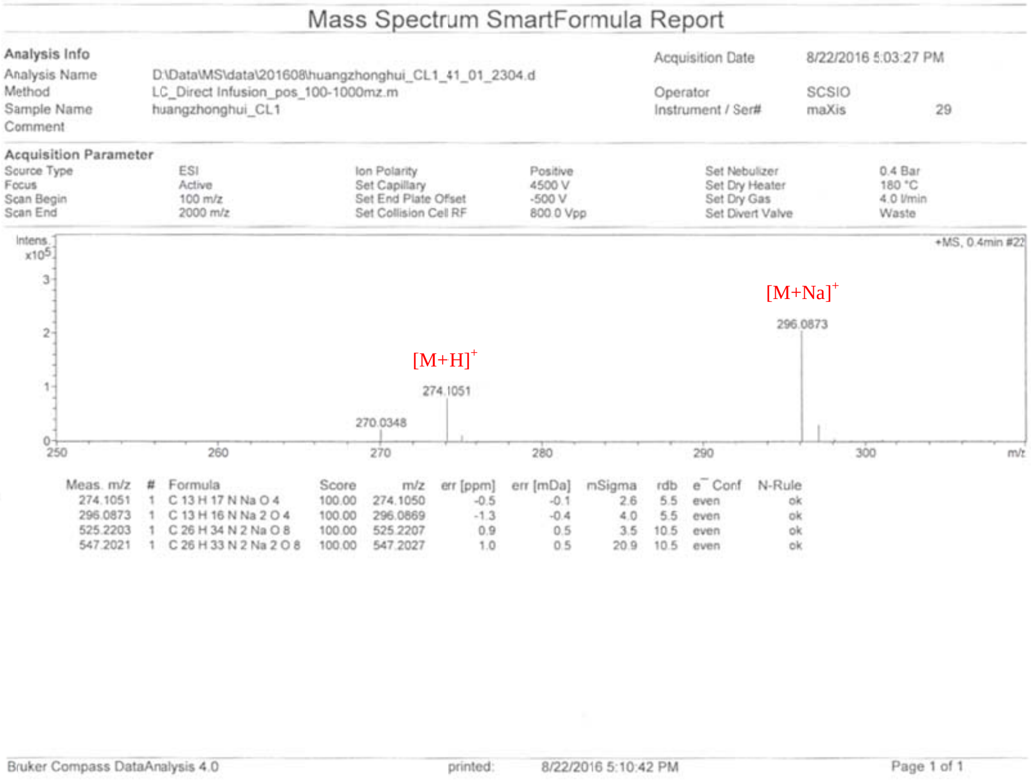

Mass Spectrum SmartFormula Report

Analysis Info

Analysis Name	D:\Data\MS\data\201608\huangzhonghui_CL1_41_01_2304.d	Acquisition Date	8/22/2016 5:03:27 PM
Method	LC_Direct Infusion_pos_100-1000mz.m	Operator	SCSIO
Sample Name	huangzhonghui_CL1	Instrument / Ser#	maXis 29
Comment			

Acquisition Parameter

Source Type	ESI	Ion Polarity	Positive	Set Nebulizer	0.4 Bar
Focus	Active	Set Capillary	4500 V	Set Dry Heater	180 °C
Scan Begin	100 m/z	Set End Plate Offset	-500 V	Set Dry Gas	4.0 l/min
Scan End	2000 m/z	Set Collision Cell RF	800.0 Vpp	Set Divert Valve	Waste

Meas. m/z	#	Formula	Score	m/z	err [ppm]	err [mDa]	mSigma	rdb	e⁻ Conf	N-Rule
274.1051	1	C 13 H 17 N Na O 4	100.00	274.1050	-0.5	-0.1	2.6	5.5	even	ok
296.0873	1	C 13 H 16 N Na 2 O 4	100.00	296.0869	-1.3	-0.4	4.0	5.5	even	ok
525.2203	1	C 26 H 34 N 2 Na O 8	100.00	525.2207	0.9	0.5	3.5	10.5	even	ok
547.2021	1	C 26 H 33 N 2 Na 2 O 8	100.00	547.2027	1.0	0.5	20.9	10.5	even	ok

Bruker Compass DataAnalysis 4.0 printed: 8/22/2016 5:10:42 PM Page 1 of 1

Figure S53. The (+)-HRESIMS spectrum of cladosporiumin G

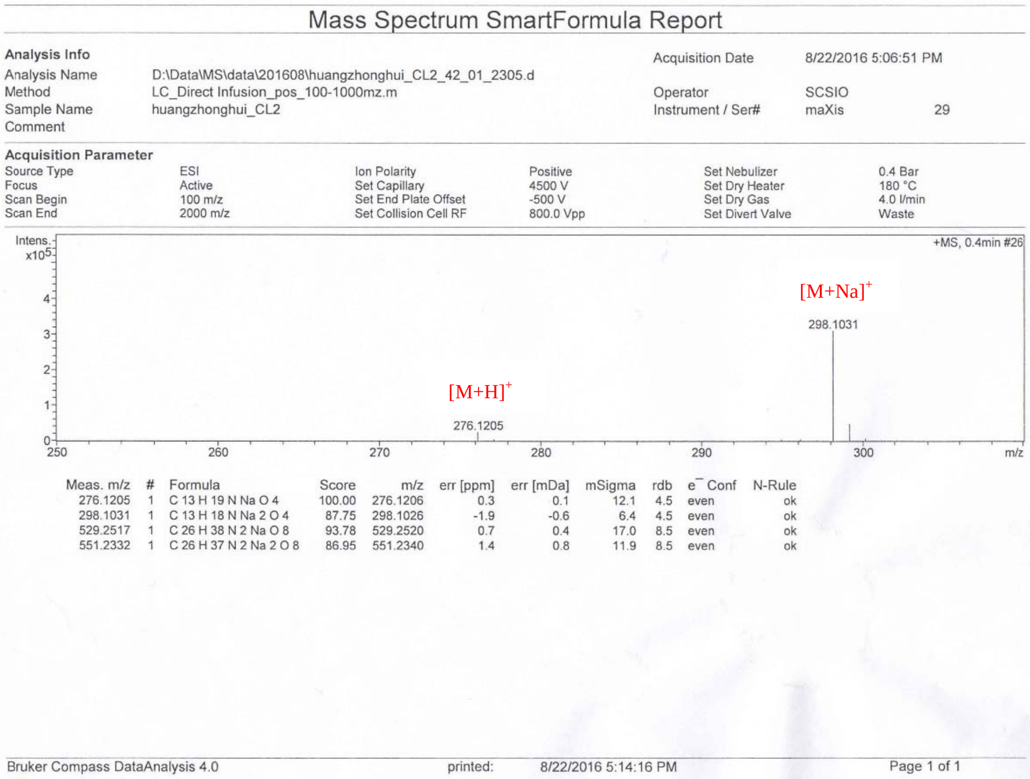

Mass Spectrum SmartFormula Report

Analysis Info

Analysis Name	D:\Data\MS\data\201608\huangzhonghui_CL2_42_01_2305.d	Acquisition Date	8/22/2016 5:06:51 PM
Method	LC_Direct Infusion_pos_100-1000mz.m	Operator	SCSIO
Sample Name	huangzhonghui_CL2	Instrument / Ser#	maXis 29
Comment			

Acquisition Parameter

Source Type	ESI	Ion Polarity	Positive	Set Nebulizer	0.4 Bar
Focus	Active	Set Capillary	4500 V	Set Dry Heater	180 °C
Scan Begin	100 m/z	Set End Plate Offset	-500 V	Set Dry Gas	4.0 l/min
Scan End	2000 m/z	Set Collision Cell RF	800.0 Vpp	Set Divert Valve	Waste

Meas. m/z	#	Formula	Score	m/z	err [ppm]	err [mDa]	mSigma	rdb	e⁻ Conf	N-Rule
276.1205	1	C 13 H 19 N Na O 4	100.00	276.1206	0.3	0.1	12.1	4.5	even	ok
298.1031	1	C 13 H 18 N Na 2 O 4	87.75	298.1026	-1.9	-0.6	6.4	4.5	even	ok
529.2517	1	C 26 H 38 N 2 Na O 8	93.78	529.2520	0.7	0.4	17.0	8.5	even	ok
551.2332	1	C 26 H 37 N 2 Na 2 O 8	86.95	551.2340	1.4	0.8	11.9	8.5	even	ok

Bruker Compass DataAnalysis 4.0 printed: 8/22/2016 5:14:16 PM Page 1 of 1

Figure S54. The (+)-HRESIMS spectrum of cladosporiumin F

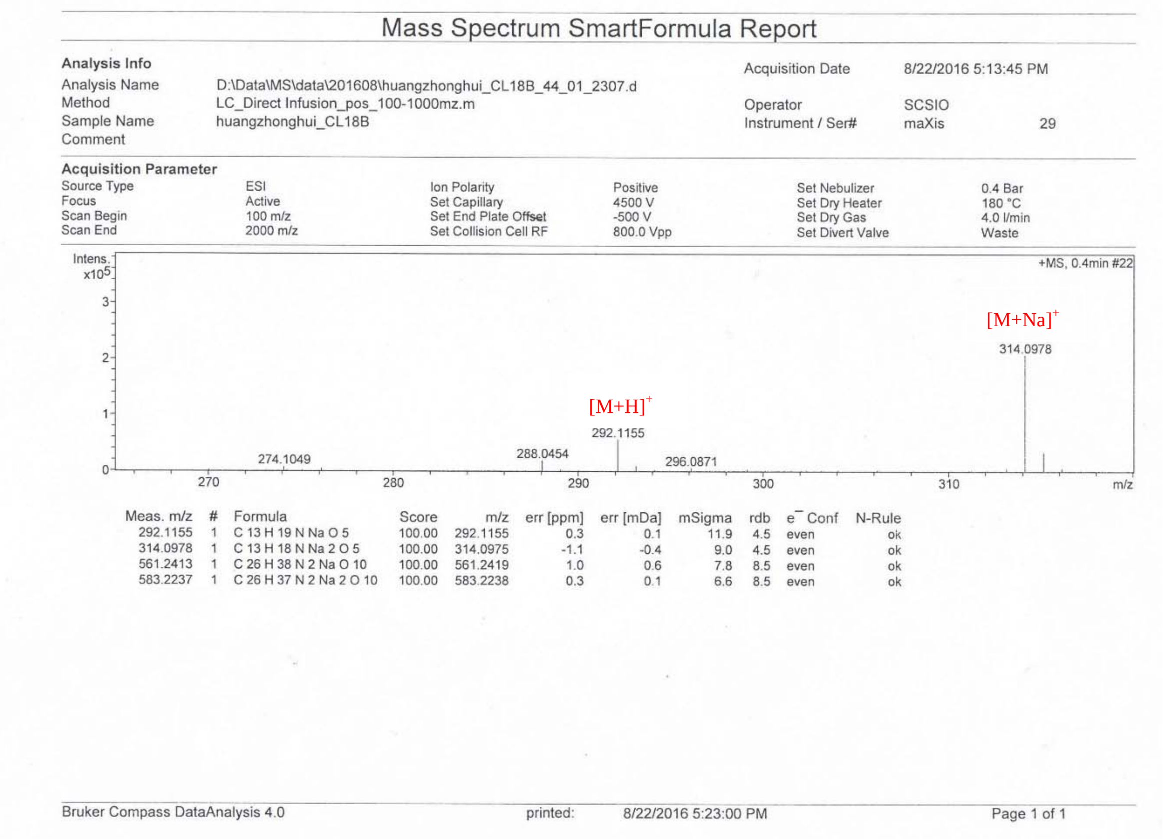

Mass Spectrum SmartFormula Report

Analysis Info

Analysis Name	D:\Data\MS\data\201608\huangzhonghui_CL18B_44_01_2307.d	Acquisition Date	8/22/2016 5:13:45 PM
Method	LC_Direct Infusion_pos_100-1000mz.m	Operator	SCSIO
Sample Name	huangzhonghui_CL18B	Instrument / Ser#	maXis 29
Comment			

Acquisition Parameter

Source Type	ESI	Ion Polarity	Positive	Set Nebulizer	0.4 Bar
Focus	Active	Set Capillary	4500 V	Set Dry Heater	180 °C
Scan Begin	100 m/z	Set End Plate Offset	-500 V	Set Dry Gas	4.0 l/min
Scan End	2000 m/z	Set Collision Cell RF	800.0 Vpp	Set Divert Valve	Waste

Meas. m/z	#	Formula	Score	m/z	err [ppm]	err [mDa]	mSigma	rdb	e⁻ Conf	N-Rule
292.1155	1	C 13 H 19 N Na O 5	100.00	292.1155	0.3	0.1	11.9	4.5	even	ok
314.0978	1	C 13 H 18 N Na 2 O 5	100.00	314.0975	-1.1	-0.4	9.0	4.5	even	ok
561.2413	1	C 26 H 38 N 2 Na O 10	100.00	561.2419	1.0	0.6	7.8	8.5	even	ok
583.2237	1	C 26 H 37 N 2 Na 2 O 10	100.00	583.2238	0.3	0.1	6.6	8.5	even	ok

Bruker Compass DataAnalysis 4.0 printed: 8/22/2016 5:23:00 PM Page 1 of 1

Figure S55. The (+)-HRESIMS spectrum of cladosporiumin H

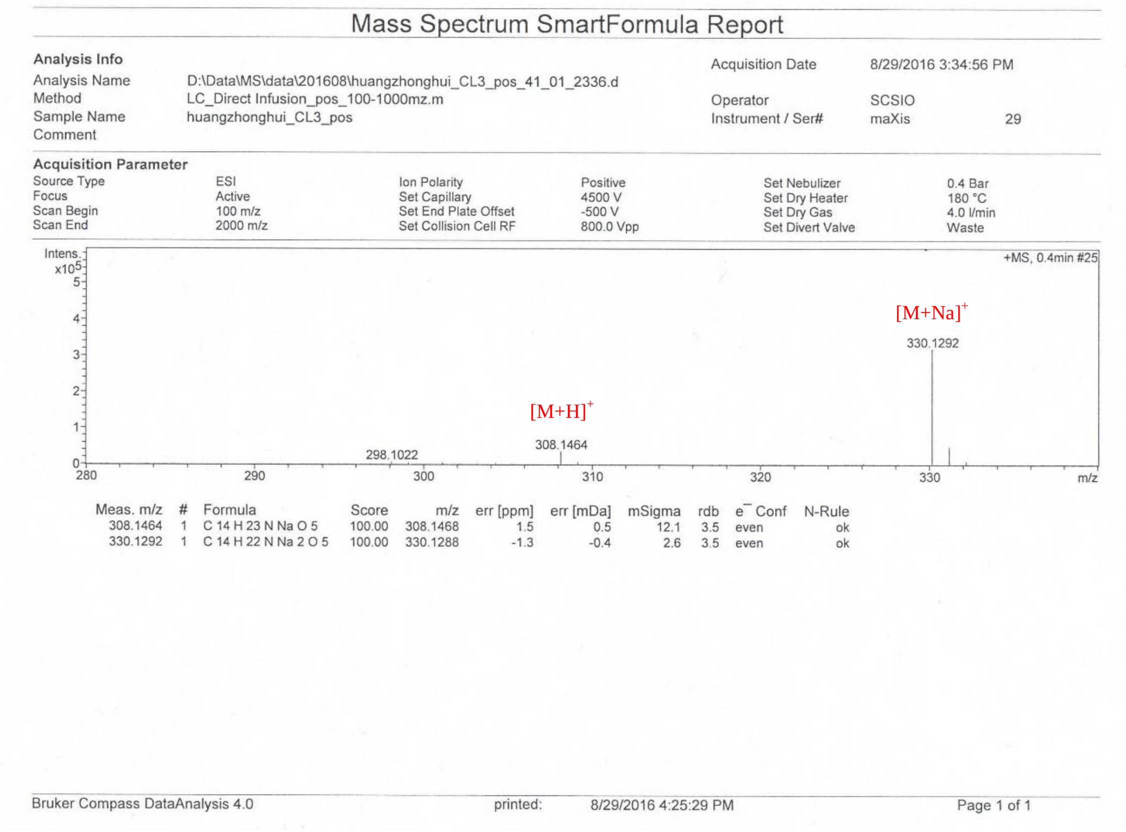

Mass Spectrum SmartFormula Report

Analysis Info

Analysis Name	D:\Data\MS\data\201608\huangzhonghui_CL3_pos_41_01_2336.d	Acquisition Date	8/29/2016 3:34:56 PM
Method	LC_Direct Infusion_pos_100-1000mz.m	Operator	SCSIO
Sample Name	huangzhonghui_CL3_pos	Instrument / Ser#	maXis 29
Comment			

Acquisition Parameter

Source Type	ESI	Ion Polarity	Positive	Set Nebulizer	0.4 Bar
Focus	Active	Set Capillary	4500 V	Set Dry Heater	180 °C
Scan Begin	100 m/z	Set End Plate Offset	-500 V	Set Dry Gas	4.0 l/min
Scan End	2000 m/z	Set Collision Cell RF	800.0 Vpp	Set Divert Valve	Waste

Meas. m/z	#	Formula	Score	m/z	err [ppm]	err [mDa]	mSigma	rdb	e⁻ Conf	N-Rule
308.1464	1	C 14 H 23 N Na O 5	100.00	308.1468	1.5	0.5	12.1	3.5	even	ok
330.1292	1	C 14 H 22 N Na 2 O 5	100.00	330.1288	-1.3	-0.4	2.6	3.5	even	ok

Bruker Compass DataAnalysis 4.0 printed: 8/29/2016 4:25:29 PM Page 1 of 1

Figure S56. Pictures of inhibition zones in the disc diffusion test

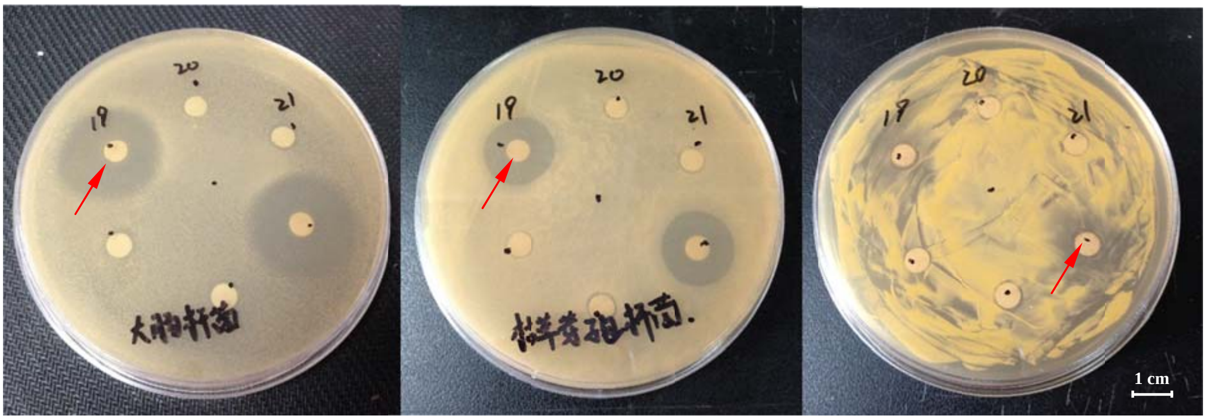

Escherichia coli ***Bacillus subtilis*** ***Micrococcus luteus***

The samples indicated by the arrow are EtOAc extracts of *Cladosporium sphaerospermum* EIODSF 008 obtained from the preliminary experiment. (100 μg/disc)

Table S1. Free energies (*G*) and equilibrium populations (P) of stable conformers of **1a** and **1b** with *R* configuration at C-10 in CH_3OH

Conformer	Conformation	*G* (kcal/mol)	P (%)		Conformer	Conformation	*G* (kcal/mol)	P (%)
(5*R*, 10*R*)-**1a-1**	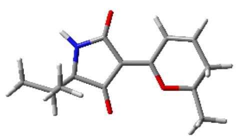	-493102.1267	42.89		(5*R*, 10*R*)-**1b-1**	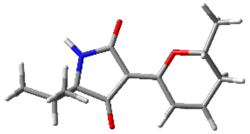	-493101.8931	28.91
(5*R*, 10*R*)-**1a-2**	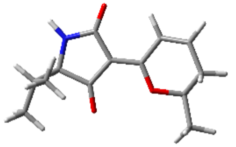	-493101.1117	7.73		(5*R*, 10*R*)-**1b-2**	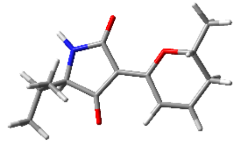	-493100.9144	5.54
(5*R*, 10*R*)-**1a-3**	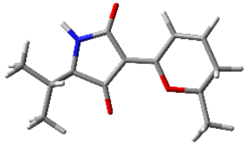	-493100.5243	2.86		(5*R*, 10*R*)-**1b-3**	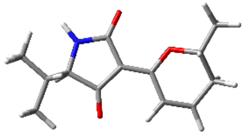	-493100.3143	2.01
(5*R*, 10*R*)-**1a-4**	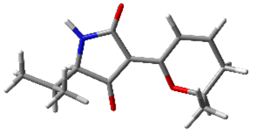	-493100.8912	5.32		(5*R*, 10*R*)-**1b-4**	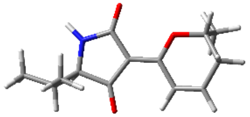	-493100.5250	2.87
(5*R*, 10*R*)-**1a-5**	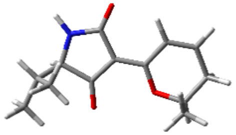	-493099.8114	0.86		(5*R*, 10*R*)-**1b-5**	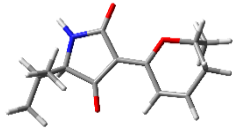	-493099.4899	0.50
(5*R*, 10*R*)-**1a-6**	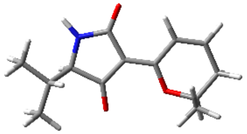	-493099.2133	0.31		(5*R*, 10*R*)-**1b-6**	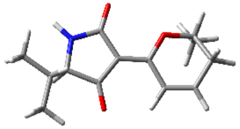	-493098.9320	0.19
			Total: 59.98					Total: 40.02

Table S2. Free energies (*G*) and equilibrium populations (P) of stable conformers of **1a** and **1b** with *S* configuration at C-10 in CH_3OH

Conformer	Conformation	*G* (kcal/mol)	P (%)		Conformer	Conformation	*G* (kcal/mol)	P (%)
(5*R*, 10*S*)-**1a-1**	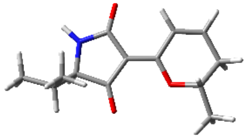	-493102.1865	47.77		(5*R*, 10*S*)-**1b-1**	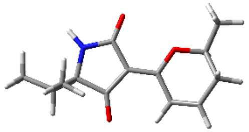	-493101.8078	25.19
(5*R*, 10*S*)-**1a-2**	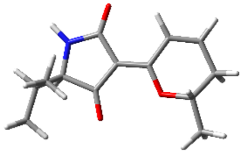	-493101.1329	8.06		(5*R*, 10*S*)-**1b-2**	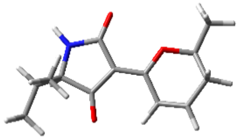	-493100.7791	4.43
(5*R*, 10*S*)-**1a-3**	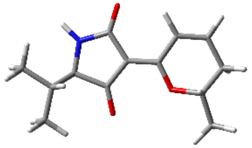	-493100.5576	3.05		(5*R*, 10*S*)-**1b-3**	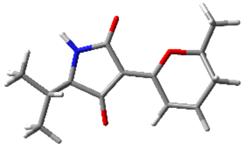	-493100.1712	1.59
(5*R*, 10*S*)-**1a-4**	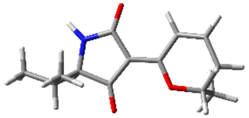	-493100.8302	4.83		(5*R*, 10*S*)-**1b-4**	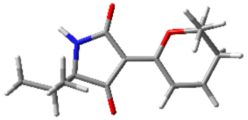	-493100.5579	3.05
(5*R*, 10*S*)-**1a-5**	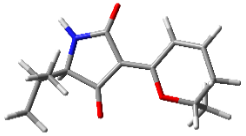	-493099.7721	0.81		(5*R*, 10*S*)-**1b-5**	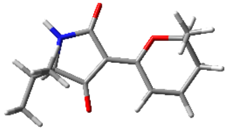	-493099.6409	0.65
(5*R*, 10*S*)-**1a-6**	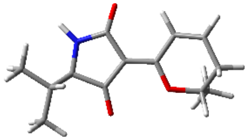	-493099.2377	0.33		(5*R*, 10*S*)-**1b-6**	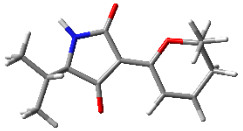	-493099.0377	0.23
			Total: 64.85					Total: 35.15

Table S3. Free energies (*G*) and equilibrium populations (P) of stable conformers of **2** with *R* configuration at C-8 in CH_3OH

Conformer	Conformation	*G* (kcal/mol)	P (%)		Conformer	Conformation	*G* (kcal/mol)	P (%)
**2-1**	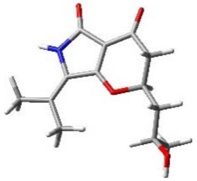	-540314.3871	19.71		2-6	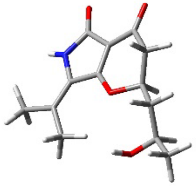	-540313.4774	4.24
**2-2**	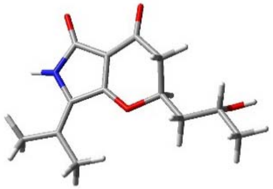	-540314.3796	19.46		**2-7**	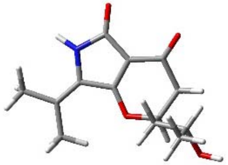	-540313.1813	2.57
**2-**3	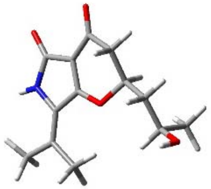	-540314.1949	14.25		**2-8**	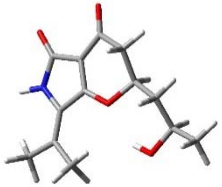	-540313.1316	2.36
**2-4**	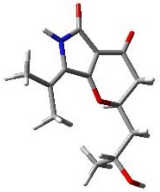	-540314.1882	14.08		**2-**9	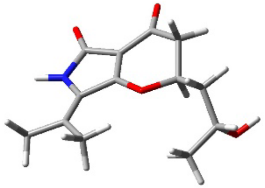	-540313.0808	2.17
**2-**5	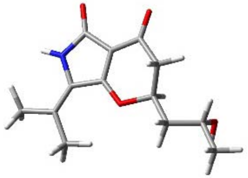	-540314.1594	13.42		**2-**10	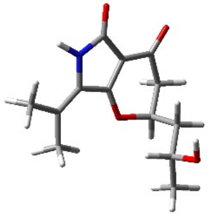	-540313.0489	2.06

**2-11**	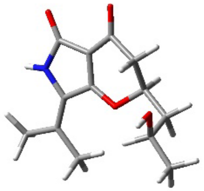	-540313.0194	1.96		**2-3**	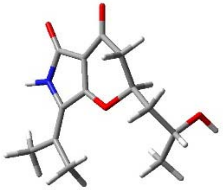	-540312.9645	1.78
**2-12**	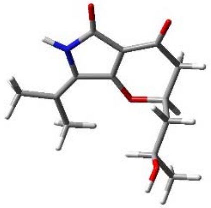	-540313.0174	1.95					
